# Inhibitory KIRs decrease HLA class II-mediated protection in Type 1 Diabetes

**DOI:** 10.1371/journal.pgen.1011456

**Published:** 2024-12-26

**Authors:** Laura Mora-Bitria, Bisrat J. Debebe, Kelly L. Miners, Kristin Ladell, Charandeep Kaur, James A. Traherne, Wei Jiang, David A. Price, Linda Hadcocks, Nicholas A. R. McQuibban, John Trowsdale, F Susan Wong, Nikolas Pontikos, Christoph Niederalt, Becca Asquith

**Affiliations:** 1 Department of Infectious Disease, Faculty of Medicine, Imperial College London, London, United Kingdom; 2 Systems Pharmacology and Medicine, Bayer AG, Leverkusen, Germany; 3 Division of Infection and Immunity, Cardiff University School of Medicine, University Hospital of Wales, Cardiff, United Kingdom; 4 Immunology Division, Department of Pathology, University of Cambridge, Cambridge, United Kingdom; 5 Systems Immunity Research Institute, Cardiff University School of Medicine, University Hospital of Wales, Cardiff, United Kingdom; 6 Institute for Infection and Immunity, St George’s, University of London, London, United Kingdom; 7 Centre for Integrative Systems Biology and Bioinformatics (CISBIO), Department of Life Sciences, Imperial College London, London, United Kingdom; 8 UCL Institute of Ophthalmology, University College London, London, United Kingdom; Yale School of Medicine, UNITED STATES OF AMERICA

## Abstract

Inhibitory killer cell immunoglobulin-like receptors (iKIRs) are a family of inhibitory receptors that are expressed by natural killer (NK) cells and late-stage differentiated T cells. There is accumulating evidence that iKIRs regulate T cell-mediated immunity. Recently, we reported that T cell-mediated control was enhanced by iKIRs in chronic viral infections. We hypothesized that in the context of autoimmunity, where an enhanced T cell response might be considered detrimental, iKIRs would have an opposite effect. We studied Type 1 diabetes (T1D) as a paradigmatic example of autoimmunity. In T1D, variation in the Human Leucocyte Antigen (HLA) genes explains up to 50% of the genetic risk, indicating that T cells have a major role in T1D etiopathogenesis. To investigate if iKIRs affect this T cell response, we asked whether HLA associations were modified by iKIR genes. We conducted an immunogenetic analysis of a case-control T1D dataset (N = 11,961) and found that iKIR genes, in the presence of genes encoding their ligands, have a consistent and significant effect on protective HLA class II genetic associations. Our results were validated in an independent data set. We conclude that iKIRs significantly decrease HLA class II protective associations and suggest that iKIRs regulate CD4^+^ T cell responses in T1D.

## Author summary

Killer immunoglobulin-like receptors (KIRs) are key regulators of the innate immune response but there is evidence that KIRs also affect adaptive immunity. We have recently demonstrated that KIRs significantly enhance CD8^+^ T cell survival and CD8^+^ T cell-mediated control of viral infections. We hypothesise that KIRs also enhance CD4^+^ T cell survival and risk of autoimmunity. We find that KIRs have a profound impact on the risk of a prototypical autoimmune disease: type 1 diabetes (T1D). The significance of this work is two-fold: first, the association we identify is one of the largest reported for T1D in recent decades. Second, it is evidence for a fundamental pathway in which innate receptors impact on CD4^+^ T cells and ultimately affect human health.

## Introduction

Type 1 Diabetes (T1D) is a common autoimmune disease characterized by insulin-deficiency due to the destruction of insulin-producing islet β-cells. The exact aetiology of T1D remains elusive, but environmental triggers are thought to initiate the break in peripheral tolerance in genetically susceptible individuals. The largest genetic contributors to susceptibility to T1D are the human leucocyte antigen (HLA) genes [1,2]. Within the HLA region, the closely linked classical class II *HLA-DRB1*, *HLA-DQB1* and *HLA-DQA1* genes display the strongest associations indicating that CD4^+^ T cells have a major role in T1D etiopathogenesis. In particular, DRB1*04:01/02/04/05-DQA1*03:01-DQB1*03:*02* and DRB1*03:01-DQA1*05:01-DQB1*02:*01* haplotypes are associated with the highest T1D susceptibility whereas DRB1*15:01-DQA1*01:02-DQB1*06:*02* is associated with dominant protection [3].

Here we study a family of inhibitory receptors called inhibitory killer-cell immunoglobulin-like receptors (iKIRs). iKIRs are expressed predominantly on natural killer (NK) cells and, at a lower frequency, on late stage differentiated T cells. The ligands of iKIRs are HLA class I molecules which they bind in broad allele groups, e.g. KIR3DL1 binds HLA-B alleles with a Bw4 motif at positions 77–83 [4]. The iKIR genes and the genes encoding their HLA class I ligands are located on different chromosomes and so are inherited independently. Consequently, it is common for individuals to have one or more iKIRs without the corresponding ligand; if an individual is positive for a given iKIR as well as the matching ligand we refer to that iKIR as “functional”. iKIRs play a major role in regulating innate NK cell-mediated responses but there is increasing evidence that iKIRs also modulate adaptive T cell responses [5–7]. In particular, iKIRs have been reported to increase activated T cell survival and to dampen effector function. The two main mechanisms of increased T cell survival are inhibition of activation-induced cell death (attributed to iKIRs expressed on T cells) and inhibition of NK cell-mediated killing of activated T cells (attributed to iKIRs expressed on NK cells) [8–11]. We have previously found that iKIRs together with their ligands significantly enhance CD8^+^ T cell survival in humans [7]. Furthermore, iKIRs with their ligands also enhance protective and detrimental HLA class I associations and have a significant impact on the clinical outcome of three different chronic viral infections [10,12]. We distinguish this modulation of protective and detrimental HLA associations by functional iKIR, which we suggest is due to a modulation of T cell responses by iKIR, from a main effect of functional iKIR, which we suggest is more likely to be NK cell-mediated [10,12]. Evidence for a main effect of iKIR or functional iKIR in T1D is weak. Since 2003, 15 studies have reported KIR gene associations with T1D risk, but none has been consistently reproduced and, in a recent meta-analysis, no associations survived correction for multiple comparisons [13]. A few studies have explored functional iKIR associations i.e. associations between iKIR-HLA ligand gene pairs and T1D [14–16] but again, results are not consistent across the studies. The impact of functional iKIR on HLA associations has not been studied.

We postulated that, given their impact on adaptive immune responses in chronic viral infections, iKIRs might play an analogous role in autoimmunity. Associations between HLA class II genes and T1D are clear evidence that CD4^+^ T cells play a role in T1D. We hypothesized that if iKIRs modulate CD4^+^ T cell responses then this should be manifest as an iKIR modulation of HLA class II genetic associations. We sought to test this hypothesis by investigating the impact of iKIRs on HLA class II disease associations in T1D using a large (N = 11,961) case-control dataset from the UK Genetics Resource Investigating Diabetes (UK-GRID). We identified a consistent and significant functional iKIR modification of HLA class II protective associations. The size of this effect was striking, for instance the odds ratio of iKIR in DQA1*01:02-DQB1*06:*02+* individuals is 6.12; one of the largest genetic effects reported in T1D in recent decades and is replicated across all protective class II genotypes. These findings are reproduced in a smaller independent dataset consisting of 342 US multiplex T1D families from the Human Biological Data Interchange (HBDI). Our immunogenetic analyses show that genes encoding iKIRs with their ligands decrease protective class II genetic associations, consistent with a picture in which iKIRs modulate T cell-mediated regulation of autoimmunity.

## Results

We previously reported that functional iKIR genes (gene pairs encoding both the iKIR and its HLA class I ligand) enhanced protective and detrimental HLA disease associations in three chronic viral infections [10,12]. Here, we asked whether in the context of autoimmunity, where an enhanced T cell response might be considered detrimental, functional iKIRs had the opposite effect, i.e. that HLA associations were not enhanced but weakened by functional iKIR genes.

We chose T1D as a paradigmatic example to study the effect of iKIRs in autoimmunity. Our primary cohort was a T1D case-control cohort which consisted of (after removal due to missingness) of 6,219 cases and 5,742 controls (see Materials and methods). We first studied the impact of iKIRs on detrimental HLA class II disease associations. We found that most detrimental class II genotypes were not impacted by iKIRs (**S2 Fig**) and that the two detrimental genotypes which were modified by iKIRs were modified in opposite directions. Overall, there was no evidence of consistent iKIR modification (P = 0.46). We next studied HLA protective class II associations starting with the best documented class II protective genotype: DRB1*15:01-DQA1*01:02-DQB1*06:*02*.

### iKIR score modifies the DRB1*15:01-DQA1*01:02-DQB1*06:*02* protective association

The DRB1*15:01-DQA1*01:02-DQB1*06:*02* compound genotype has repeatedly been described to confer protection from T1D [17] and this protective effect is reproduced in our cohort (ln[OR] = -3.75, P = 1.05x10^-157^). There is some evidence that the protection associated with the DRB1*15:01-DQA1*01:02-DQB1*06:*02* genotype maps to DQA1*01:02-DQB1*06:*02* (henceforth *DQ6*), which encodes the DQ6 molecule, rather than DRB1*15:01-DQB1*06:*02* (henceforth *DR15*) [18,19]. In our cohort, virtually everyone who carries *DQ6* also carries *DR15* (99.2% of *DQ6* positive individuals are *DR15* positive) so it is difficult to fine map the protective genotype. When both *DQ6* and *DR15* are included simultaneously in a regression analysis, *DQ6* retains significance (ln[OR] = -2.86, P = 6.1x10^-3^) whereas *DR15* becomes non-significant (ln[OR] = -0.9, P = 0.4) in line with the literature [18,19]. We therefore focused our analysis on *DQ6*. However, results focusing instead on *DR15* (either as a phased haplotype or compound genotype) are virtually identical (see **S1 Results**).

For each individual we calculated their “iKIR score”, a value equal to the count of iKIR-ligand gene pairs in that individual weighted by the strength of the iKIR-HLA interaction (**S1 Materials and methods** and [10]) so that a large iKIR score reflects someone with a large number of strong iKIR-ligand interactions. iKIR score is protective in our cohort (ln[OR] = -0.22, P = 2.8x10^-25^). However, in a model including iKIR ligands (Bw4, C1 and C2) as covariates, the iKIR score association is lost (ln[OR] = -0.008, P = 0.85) and Bw4 is strongly protective (ln[OR] = -0.45, P = 9.1x10^-16^). Therefore, the iKIR score association is probably driven by KIR ligands alone; for example, by the protective or detrimental effect of B*57:*01* (Bw4-80I motif) and A*24:*02* (Bw4-80I motif) alleles respectively [20]. We conclude that iKIR score as a main effect does not contribute to T1D risk and move to investigate the impact of iKIR score on the HLA class II *DQ6* association.

**Fig 1:**
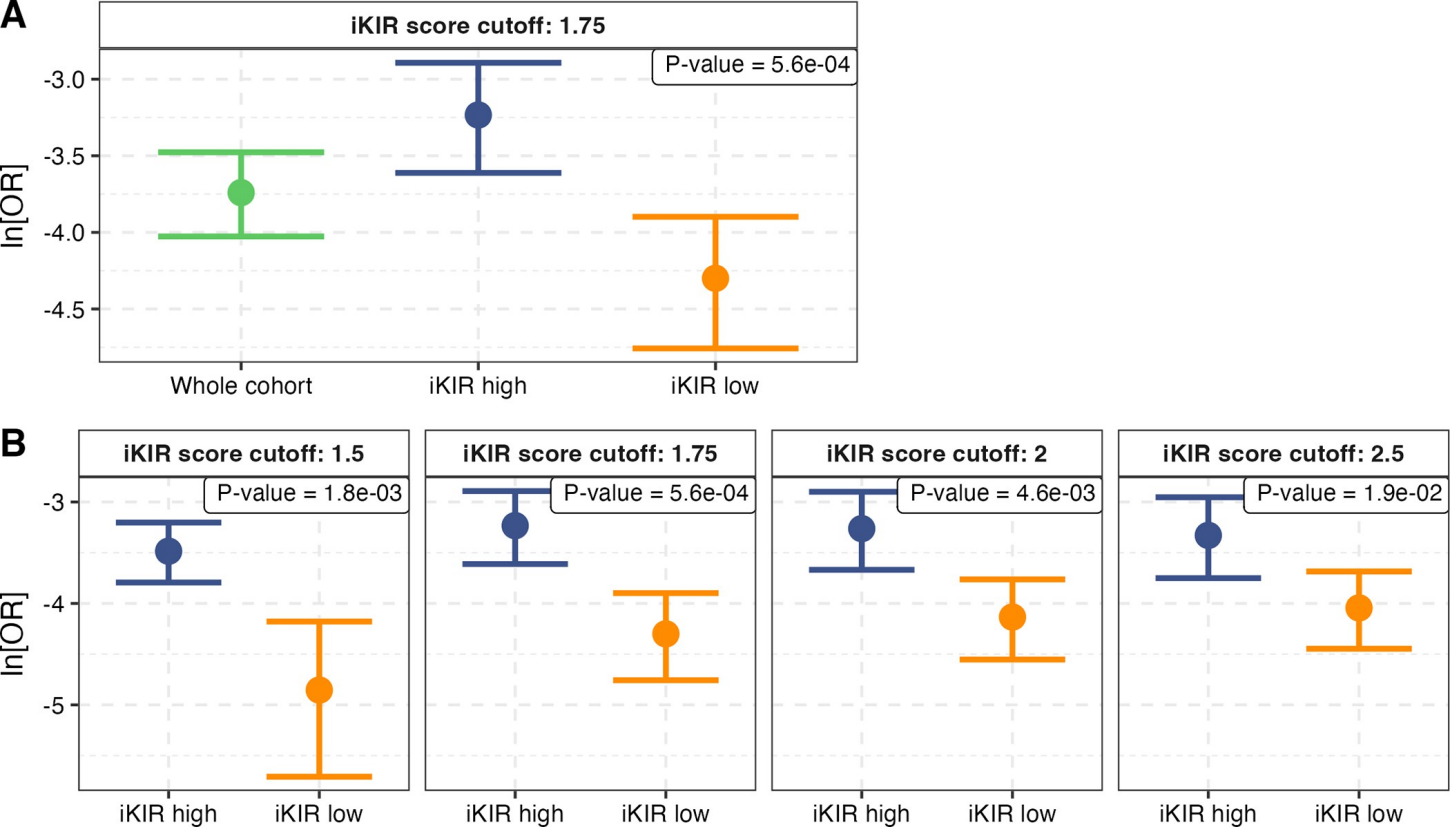
*DQ6*-associated protection is enhanced amongst individuals with low number of functional iKIR. **A** The protective effect of *DQ6* is enhanced in the group of individuals with an iKIR score equal to 1.75 or lower (iKIR score threshold **=** 1.75). The number in the top right box corresponds to the odds of seeing this enhancement by chance (Permutation test, see **Materials and methods**). **B** The same analysis was repeated using different iKIR score thresholds to define “high” and “low” (1.5, 1.75, 2.0 and 2.5, indicated in grey box above the relevant plot). The observed effect is robust to the exact choice of threshold. In all cases the dot is the natural log of the odds ratio, ln[OR], and the bars the 95% confidence intervals obtained from the logistic regression; green: whole cohort, blue high iKIR score strata, yellow: low iKIR score strata. ln[OR]<0 indicates protection, with greater protection associated with a lower ln[OR]. Coefficients, p-values and group sizes are reported in **S1 Table**.

On stratifying the cohort into individuals with a high iKIR score (>1.75) and individuals with a low iKIR score (≤1.75) we found that the protective effect of *DQ6* varied significantly between the strata as we had seen for class I effects in the context of virus infection, but as expected the effect was reversed with the strongest class II protection seen in individuals with a low iKIR score (**Fig 1A**). The odds of seeing this by chance were found, by permutation test, to be P = 5.6×10^−4^. The iKIR score threshold selected to categorise someone as having a “high” iKIR score (threshold = 1.75) was chosen to give a balanced stratification. We investigated whether our result, that the protective effect of *DQ6* was stronger amongst people with a low iKIR score than amongst people with a high iKIR score, was dependent on the choice of threshold. The small number of cases having this protective genotype makes exploration of more extreme thresholds problematic as the number of individuals in one stratum may be quite sparse. Nevertheless, we explored 4 different thresholds: 1.5, 1.75, 2.0 and 2.5. In every case the result was replicated (**Fig 1B and S1 Table**). These observations are not independent, but they do demonstrate that our result is robust to the choice of threshold.

### *DQ6* protection decreases as a function of iKIR score

If iKIR score is a meaningful measure, we might expect a “dose effect” i.e. the impact on the protective class II effect depends on the value of the iKIR score. To investigate this, rather than stratifying the cohort, we modelled the whole cohort and included iKIR score in the model as a continuous variable interacting with *DQ6 (i.e.* OUTCOME∼DQ6×iKIR_score+GENDER). The interaction term was significant (P = 6.57×10^−7^, model AIC = 14271); this is stronger than if we included stratified iKIR score as an interaction term (P = 1.93×10^−4^, model AIC = 14264) consistent with a dose effect.

To confirm that there was a dose effect we stratified the cohort into individuals with a low, intermediate, and high iKIR score and in each of the three strata calculated the protective effect of *DQ6*. Again, this analysis is problematic as the strong protective effect of *DQ6* is such that, although our cohort is very large, there are only 54 cases with the protective genotype. Due to the inevitable low numbers per strata, results would be expected to be noisy and subject to exact choice of stratification. Therefore, we considered all possible strata choices yielding 12 or more cases in each stratum. For each stratification we found the same picture, the ln[OR] decreased in a dose-dependent manner as the iKIR score decreased (**S3A Fig**). In short, with both regression by interaction and by stratification we reached the same conclusions: *DQ6* protection decreases as a function of iKIR score.

We also assessed whether iKIR score was a better predictor of impact than iKIR count (**S1 Text**). The iKIR score has a marginally stronger effect than the iKIR count on the protective effect of *DQ6* (P = 6.57×10^−7^ for iKIR score, P = 1.1×10^−6^ for iKIR count); in backwards stepwise regression (starting from a full model with both interaction terms) iKIR count is removed from the model and the model with iKIR Count has a higher AIC than a model with iKIR score (AIC difference = 10). However, both terms are very similar and the difference in coefficient (for standardised variables) is very small (0.69 for the score 0.68 for the count) making it difficult to reach firm conclusions but on balance the effect seems to be better predicted by the iKIR score.

Additional analysis concluded that (1) the observed modification of *DQ6* cannot be explained by the HLA class I genes alone (2) all iKIR genes contribute to the *DQ6* modification (3) KIR modification of *DQ6* was most likely to be explained by inhibitory KIR rather than activating KIR (though the two are closely correlated) and (4) iKIR modification of *DQ6* is independent of the detrimental genotypes DRB1*03:01-DQB1*02:*01* and DRB1*04:01/02/04/05-DQB1*03:*02* (**S1 Results**).

### Other protective HLA class II *DRB1-DQB1* haplotypes are also modulated by iKIRs

Having established that a low iKIR score is associated with a significant increase in the protection conferred by the prototypical protective HLA class II genotype *DQ6*, we hypothesized that other significantly protective haplotypes or genotypes in our cohort would also be iKIR score modified. The strongest genetic associations reported in the literature have been with *DRB1-DQB1* haplotypes, so we initially focused on phased *DRB1*-*DQB1* haplotypes.

In the whole cohort we found 17 *DRB1-DQB1* haplotypes that were significantly protective. For every case, with the exception of one haplotype (DRB1*07:01-DQB1*03:*03*), we found the same effect i.e. the protection conferred by HLA class II haplotypes was weakened in the presence of a high iKIR score and strengthened in the presence of a low iKIR score (**S4A Fig and S3 Table**). Several haplotypes are present at low frequency in our cohort so some results may have arisen by chance. The overall probability of our observation i.e. that iKIR score modified the protection of the 17 *DRB1-DQB1* protective haplotypes was assessed by a permutation test (see **Materials and methods**). The test statistic was the weighted mean of the iKIR effect (ln[OR] in KIR high–ln[OR] KIR low weighted by the haplotype frequency). We found that out of 3×10^7^ permutations there was never a case where the test statistic of the permuted dataset was as extreme as the observed value (**S4B Fig**), so we conclude that the effect of iKIR on protective haplotypes is unlikely to have arisen by chance (odds of seeing the effect across protective haplotypes P<3×10^−7^).

Although the finding that the protective *DRB1-DQB1* haplotypes were significantly modified by functional iKIRs is interesting we were aware of two potential caveats. First, the *DRB1-DQB1* haplotypes may not be the causal drivers of protection, they could just be neutral passengers marking class II genotypes that are more closely associated with protection (i.e. we are focusing on the wrong target). Second, linkage disequilibrium between the class II haplotypes and protective or detrimental HLA class I alleles (which are also iKIR ligands) could be mistaken for iKIR modification of the class II protective effect. We therefore investigated both these possibilities.

### Which alleles are best associated with protection?

We suspected that some of the *DRB1-DQB1* haplotypes may not be protective themselves but that they were neutral passengers marking the true, causal driver genotypes. Therefore, we aimed to understand whether the *DRB1* and *DQB1* genes of the protective haplotype themselves or other class I or class II alleles best marked the protective effect (and if the latter whether these protective alleles were also modulated by iKIR score).

**Fig 2:**
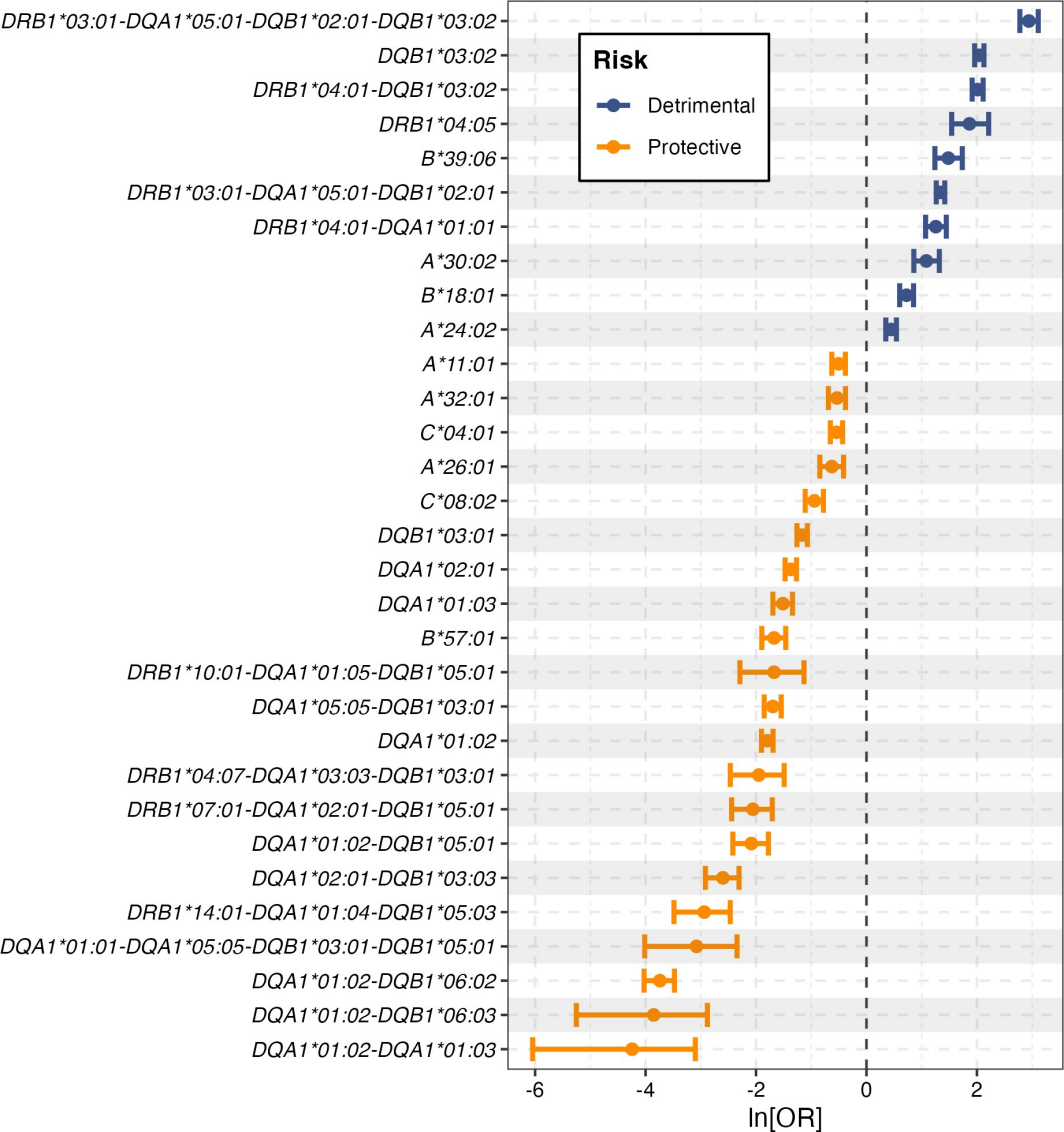
Forest plot with all HLA drivers associated with T1D. We show all class I and class II driver genotypes identified (see **S1 Materials and methods**). The regression coefficients plotted, i.e. the ln[OR], are for an analysis in the UK-GRID cohort in which the only other covariate was gender. The coefficients will depend both on the genetic risk of the whole cohort (i.e. the background that the risk/protection is measured relative to) and the other genotypes correlated with the genotype of interest. Note that although DRB1*03:01-DQA1*05:01-DQB1*02:01-DQB1*03:*02* appears to be a compound of the detrimental genotype DRB1*03:01-DQA1*05:01-DQB1*02:*01* and the detrimental genotype DQB1*03:*02* it was retained in the list of independent drivers as it retained direction and significance of effect (albeit considerably weakened) in multiple regression when both DRB1*03:01-DQA1*05:01-DQB1*02:*01* and DQB1*03:*02* were included simultaneously with it. We cannot rule out the possibility that these genotypes mark unsequenced variants that are even more closely identified with outcome, but we can say they are the class I and class II alleles most closely associated with outcome. Coefficients, p-values and number of cases and controls are provided in **S4 Table**.

We investigated all HLA class I and HLA class II alleles as well as two and three allele genotypes at *DRB1*, *DQA1* and *DQB1* (considered in cis and in trans as both trans-acting and cis-acting associations have been documented [3]). We considered all pairs of genotypes in a regression model (a total of 235,347,360 pairwise combinations) and defined “drivers” to be genotypes that never lost significance nor swapped direction in the presence of another genotype. Of course, we cannot rule out the possibility that these genotypes mark unsequenced variants that are even more significantly associated with outcome, but we can say they are the most significantly associated of the HLA class I and class II alleles. We identified 10 HLA class I alleles and 21 class II genotypes (single alleles, pairs or trios) in our cohort significantly associated with T1D independently of all other genotypes in the cohort (**Fig 2 and S4 Table**). Establishing a list of the class II driver genotypes is difficult due to the strong linkage disequilibrium across the HLA region and in the case of colinear or close to colinear genotypes, simplifying assumptions had to be made (see **S1 Materials and methods**). We adjusted for multiple comparisons using the effective number of tests (M_eff_ = 3,692, calculated from the correlation structure of the original 21,696 genotypes considered, **S1 Materials and methods**) and applied the Bonferroni correction (0.05/M_eff_); this gave a cutoff for significance of P_adjusted_ = 1.35×10^−5^. All 31 driver genotypes remained significant. This remained true even when assuming that all genotypes tested are independent (m = 21,696; P_adjusted_ = 0.05/m = 2.3×10^−6^) or when using the typical significance level in GWAS studies (P = 5×10^−8^).

As anticipated, none of the 17 protective *DRB1-DQB1* haplotypes studied above were the drivers of protection. Instead, we identified 15 protective HLA class II genotypes which were better associated with outcome. Henceforth we focus on these 15 protective genotypes.

### Impact of HLA class I drivers

Our second concern was that correlations between the protective class II genotypes of interest and the driver class I genotypes (some of which are also iKIR ligands) could be mistaken for iKIR modification. For example, HLA-B*57:*01* is a class I allele which is significantly associated with protection. It also encodes the ligand for KIR3DL1 and as such will be enriched in the iKIR-high strata. If a protective class II genotype is associated with B*57:*01* then individuals with the class II genotype and B*57:*01* will be more likely to appear in the high iKIR strata; any additive effects of protection from the class II genotype and B*57:*01* would then risk either being misinterpreted as iKIR modification of the class II genotype or risk masking an iKIR modification (depending on the direction of the correlations). To remove this possibility, we removed all individuals who were positive for any of the class I driver alleles from the cohort, leaving a reduced cohort of size N = 5,420. Simply removing class I drivers is the cleanest approach to dealing with the problem. In many immunogenetics analyses this is not an option due to the reduction in cohort size; in this case we are fortunate in starting with a very large cohort which allows removal of the class I drivers.

### Protective effects of HLA class II genotypes are significantly modified by iKIR

**Fig 3:**
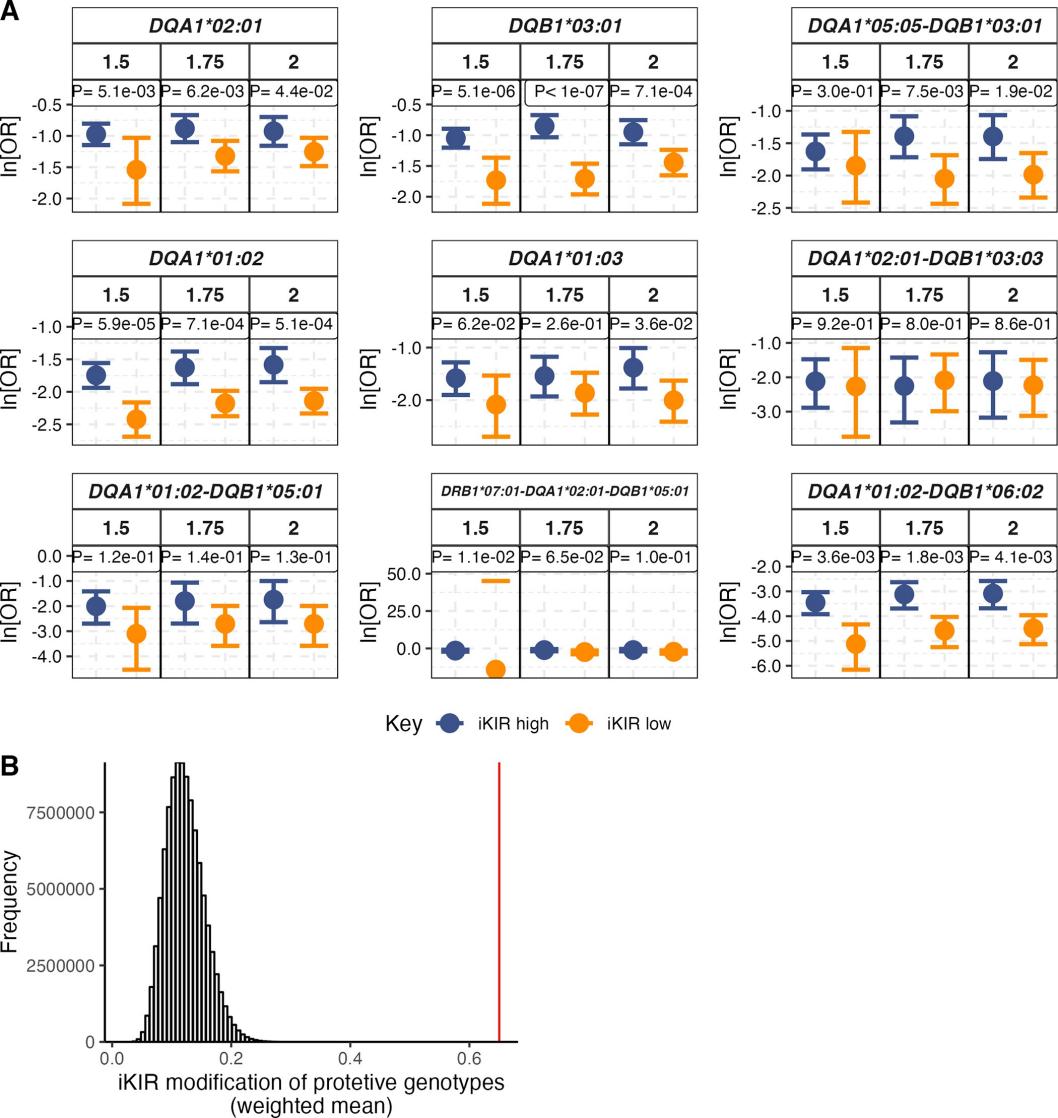
iKIR score negatively impacts the protective effect of 9 driver HLA class II genotypes in T1D. **A** The 9 protective class II genotypes investigated were all modified by iKIR at the three thresholds (definition of iKIR high and low) considered. The only two genotypes for which the effect was not strong (DQA1*02:01-DQB1*03:*03* and DQA1*01:02-DQB1*05:*01*) were very infrequent (N **=** 12 and N **=** 14 cases respectively). **B** The observed value of our test statistic (weighted mean of the difference in ln[OR] between the KIR high and the KIR low strata at threshold **=** 1.75), indicated by the red line, lies far above the distribution (grey histogram) of the same test statistic under the null hypothesis that the iKIR score has no impact on the protective genotypes (generated by permuting the iKIR score of individuals in the cohort). Indicating that the probability of obtaining our observation by chance is extremely low (P **=** 2×10^−8^).

We stratified this reduced cohort into individuals with high iKIR score and individuals with a low iKIR score and analyzed the effect of the protective class II drivers within each stratum; class II drivers which were carried by fewer than 10 cases were not studied as numbers were too low to permit stratification. The nine remaining protective genotypes were all modified in the same direction at all three thresholds considered (**Fig 3A and Table 1**). The only genotypes for which the iKIR effect was not extremely clear (DQA1*0201-DQB1*03:*03* and DQA1*01:02-DQB1*05:*01*) were very infrequent (N = 12 and N = 14 cases respectively) so the number of carriers in each strata would be very low possibly explaining the lack of a clear modification. Overall, the odds of observing this iKIR modification across the 9 genotypes was very low, P = 2×10^−8^ (**Fig 3B**). Furthermore, we also modelled the whole cohort and included iKIR score as an interaction term with a given protective genotype. The iKIR interaction term was significant in the 4 out of 5 most frequent genotypes and in the expected direction in all genotypes (**Table 2**). We conclude that iKIR negatively impacts the effects of all protective class II genotypes in T1D, that this effect is attributable to iKIR-HLA receptor ligand interactions and unlikely to be observed by chance.

**Table 1:** iKIR score decreases protection associated with protective class II genotypes in T1D. The UK-GRID cohort without carriers of HLA class I drivers (N **=** 5,420) was stratified into individuals with high or low iKIR score at a 1.75 cutoff (we also tested cutoffs 1.5 and 2.0, see **S5 Table**). The protective effect of each genotype was evaluated independently in each stratum using multivariate logistic regression with gender as covariate. HLA class II protection is enhanced in the iKIR low strata for all genotypes but for the very infrequent protective genotype DQA1*02:01-DQB1*03:*03*. Regression coefficients, permutation p-values and cohort sizes are reported for the different strata. P-value for the whole cohort (unstratified analysis) calculated using the Wald-test; p-values for the stratification analysis are calculated using the permutation test.

Genotype	Group	ln[OR]	2.50%	97.50%	P-value	N Genotype +		N Genotype -	
						Cases	Controls	Cases	Controls
DQA1*01:02-DQB1*06:*02*	Whole cohort	-3.91	-4.33	-3.53	1.76×10^−83^	26	729	2984	1681
	iKIR high	-3.12	-3.69	-2.63	1.8×10^−3^	15	279	1072	882
	iKIR low	-4.58	-5.25	-4.03		11	450	1912	799
DQA1*02:01-DQB1*03:*03*	Whole cohort	-2.21	-2.87	-1.65	9.05×10^−13^	12	85	2998	2325
	iKIR high	-2.25	-3.31	-1.43	0.8	5	49	1082	1112
	iKIR low	-2.09	-2.99	-1.34		7	36	1916	1213
DQA1*01:02-DQB1*05:*01*	Whole cohort	-2.32	-2.92	-1.80	4.14×10^−16^	14	109	2996	2301
	iKIR high	-1.80	-2.69	-1.06	0.14	7	44	1080	1117
	iKIR low	-2.71	-3.58	-1.99		7	65	1916	1184
DRB1*07:01-DQA1*02:01-DQB1*05:*01*	Whole cohort	-1.69	-2.37	-1.10	1.40×10^−7^	12	52	2998	2358
	iKIR high	-1.13	-1.94	-0.41	0.065	9	29	1078	1132
	iKIR low	-2.45	-3.89	-1.39		3	23	1920	1226
DQA1*01:*02*	Whole cohort	-1.91	-2.07	-1.76	8.14×10^−133^	252	922	2758	1488
	iKIR high	-1.63	-1.88	-1.38	7.1×10^−4^	88	359	999	802
	iKIR low	-2.18	-2.38	-1.98		164	563	1759	686
DQA1*05:05-DQB1*03:*01*	Whole cohort	-1.74	-1.99	-1.51	6.015×10^−46^	88	355	2922	2055
	iKIR high	-1.39	-1.72	-1.08	7.5×10^−3^	53	198	1034	963
	iKIR low	-2.05	-2.44	-1.69		35	157	1888	1092
DQA1*01:*03*	Whole cohort	-1.75	-2.03	-1.49	2.12×10^−36^	67	279	2943	2131
	iKIR high	-1.54	-1.93	-1.18	0.25	35	156	1052	1005
	iKIR low	-1.86	-2.27	-1.48		32	123	1891	1126
DQA1*02:*01*	Whole cohort	-1.15	-1.31	-0.99	2.02×10^−45^	256	548	2754	1862
	iKIR high	-0.88	-1.10	-0.67	6.2×10^−3^	149	322	938	839
	iKIR low	-1.32	-1.57	-1.08		107	226	1816	1023
DQB1*03:*01*	Whole cohort	-1.25	-1.39	-1.11	5.59×10^−69^	361	777	2649	1633
	iKIR high	-0.85	-1.03	-0.67	<1×10^−8^	269	505	818	656
	iKIR low	-1.71	-1.96	-1.46		92	272	1831	977

**Table 2:** iKIR score interaction terms with protective HLA class II drivers in a subcohort without HLA class I drivers. After removal of class I drivers from the cohort (remaining cohort: N **=** 5,420), we modelled risk of T1D for each protective genotype and included iKIR score in the model as a continuous variable interacting with the HLA class II genotype (OUTCOME∼HLAclassIIgenotype×iKIR_score+GENDER). The coefficient for the interaction term was in the expected direction for all genotypes and significant for 4 frequent genotypes.

Protective genotype	Coefficient of interaction	P-value of interaction	N cases	N controls	N total
DQA1*01:02-DQB1*06:*02*	0.94	1.14×10^−5^	26	729	755
DQB1*03:*01*	0.35	1.97×10^−5^	361	777	1138
DQA1*01:*02*	0.33	8.35×10^−5^	252	922	1174
DQA1*05:05-DQB1*03:*01*	0.30	3.53×10^−2^	88	355	443
DQA1*01:02-DQB1*05:*01*	0.65	5.54×10^−2^	256	548	804
DQA1*02:*01*	0.18	7.35×10^−2^	14	109	123
DQA1*01:*03*	0.24	1.19×10^−1^	67	279	346
DRB1*07:01-DQA1*02:01-DQB1*05:*01*	0.60	2.27×10^−1^	12	52	64
DQA1*02:01-DQB1*03:*03*	0.11	7.31×10^−1^	12	85	97

### iKIR score modification is not explained by cryptic relatedness in the UK-GRID cohort

The UK-GRID cohort consists of individuals who are self-reported as white UK, population stratification is therefore unlikely to be a major confounder. Nevertheless to investigate the impact of potential population stratification we used a generalized linear mixed model (GLMM) including iKIR score as an interaction term with a given protective genotype as fixed effect and a random effect reflecting the genetic relatedness between each pair of individuals (**S1 Materials and methods**); an approach which has shown better performance compared to a more traditional principal components approach [21]. With this approach we correct for various possible degrees of relatedness in our cohort while retaining maximum power. Performing a stratification analysis as in **Fig 3A** (or **Table 1**) was intractable with a GLMM; fitting one GLMM model with the relatedness matrix for one protective genotype took more than 72h, so obtaining a permutation p-value was not possible. Therefore, regression by interaction was used to check the influence of relatedness on iKIR score modification.

**Fig 4:**
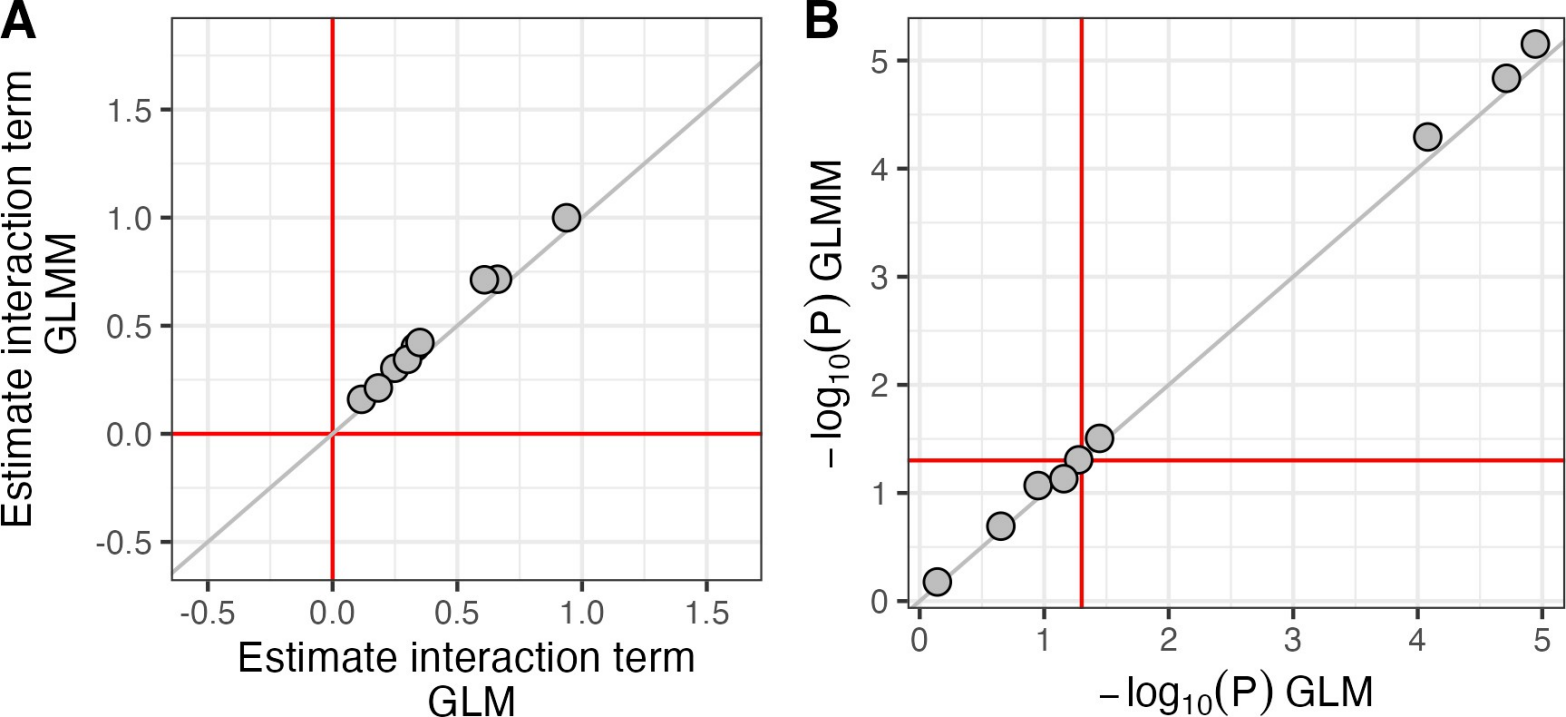
Comparison of iKIR score interaction terms for the 9 protective genotypes in a generalized linear model (GLM) vs a generalized linear mixed model (GLMM) with a relatedness matrix. **A** Estimates of the interaction term (iKIR_score:genotype) in a glm model of the form (OUTCOME∼HLAclassIIgenotype×iKIR_score) vs those obtained in a GLMM model of the form ($OUTCOME∼HLA\;class\;II\;genotype\times iKIR_{score}+(1|ID)$), where 1|ID is the random effect that maps to a relatedness matrix (see **S1 Materials and methods**). Note that the interaction coefficients positively correlate and all of them increase T1D risk (above 0 in log scale). Red lines denote value of no association (in log scale). Gray line indicates identity. **B** As for A but showing -log_10_ P-values of the interaction coefficients. Red lines now denote the significance level (α = 0.05) and the grey line is again the line of identity. That correction for relatedness has very little impact on results is to be expected given that all subjects are self-reported as white UK.

Results are summarised in **S6 Table**. By comparison with the results without correction for relatedness (**Table 2**) it can be seen that the agreement is excellent and adjusting for relatedness had very little impact on either the coefficient or the p-value for all 9 protective genotypes (**Fig 4**). We conclude that the iKIR score effect is not driven by cryptic relatedness between UK-GRID cohort individuals –adjusting for relatedness strengthens our previous observations.

### iKIR score modification is replicated in an independent cohort

To validate our findings, we studied an independent dataset consisting of 342 US multiplex families from the HBDI consortium. Prior to analyzing this cohort, we conducted a power analysis (**S1 Materials and methods**) to assess whether we had sufficient statistical power to detect a significant iKIR score effect in this much smaller cohort. We found that, assuming the effect size was the same as in the UK-GRID cohort, the family cohort would not be sufficiently powered to detect a significant iKIR score modification of individual protective class II genotype (**S5A Fig**). However, we estimated that there was sufficient power to detect an iKIR score modification if we considered several protective genotypes simultaneously i.e. DQB1*03:*01*, DQA1*02:*01*, DQA1*01:*02* and DQA1*01:02-DQB1*06:*02* (**S5B Fig**) and analyzed the family dataset on this basis. We stratified the family cohort into trios with a high iKIR score (threshold>1.75) and trios with a low iKIR score (threshold <1.75) based on the iKIR score of the affected child. For each genotype, we calculated the difference between the ratio of transmissions and non-transmissions in each strata (see **S1 Materials and methods**). The odds of observing an equal or greater difference between the ratios in each stratum under the null hypothesis was statistically significant (P = 1×10^−5^, Permutation test). These conclusions remained unchanged when using different iKIR score thresholds (**S7 Table**). We conclude that our finding that iKIR score impacts HLA class II-mediated protection was replicated in an independent dataset.

### Fraction of cases prevented by a low number of functional iKIR

**Fig 5:**
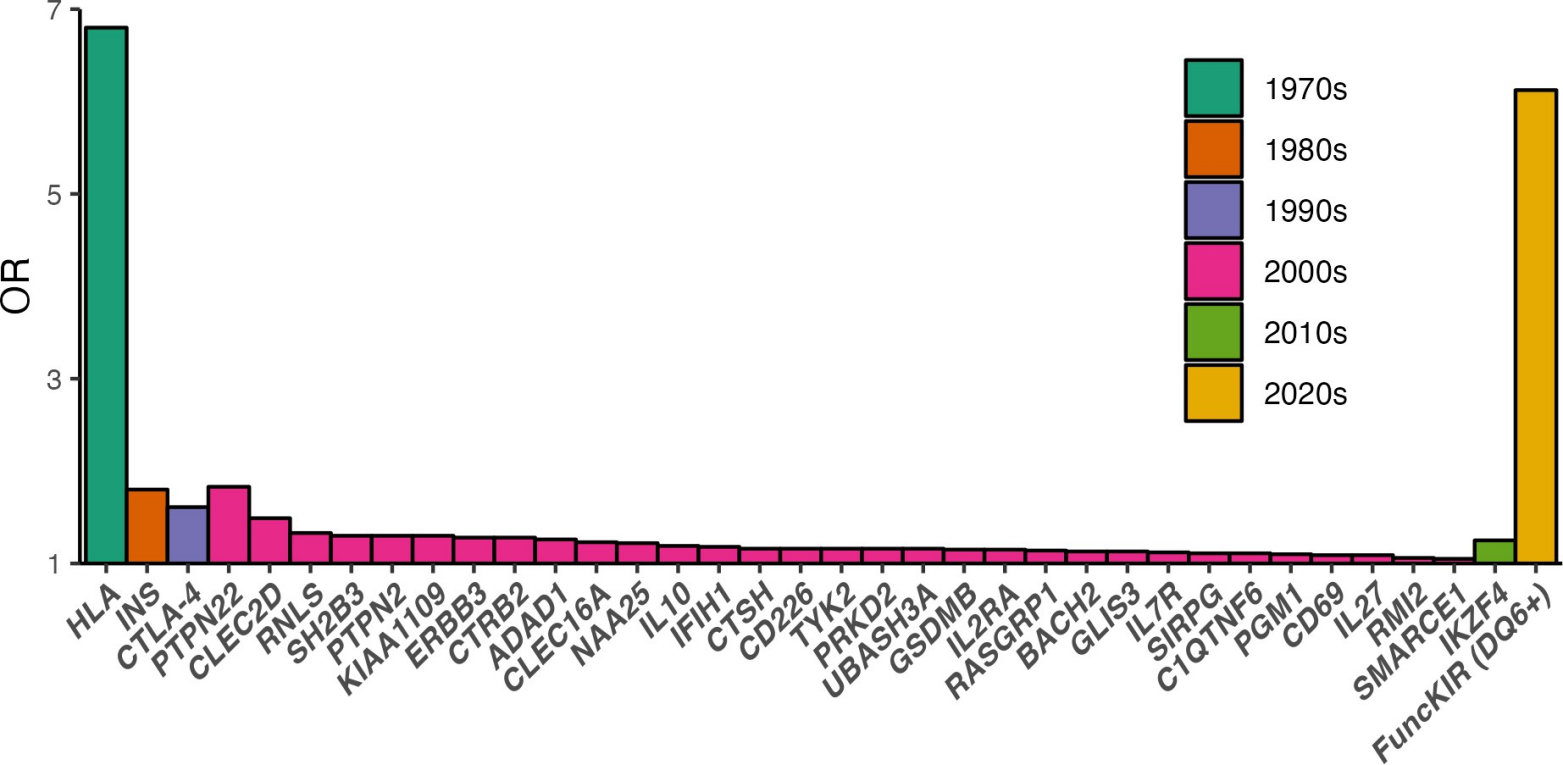
Loci that affect the risk of T1D. The OR for functional iKIR score in a *DQ6*+ cohort is shown (orange) alongside the OR for variants in other loci as reported in the literature (**S1 Materials and Methods**). Associations are grouped (and colour-coded) by decade when the association was first reported (not necessarily fine-mapped) and then within each decade, associations are ranked by size of OR. Gene names refer to most likely candidate in the region. Design of the figure is after a figure in Rich *et*. *al*.[58].

To quantify the impact of functional iKIR on disease prevalence we estimated the fraction of cases of T1D prevented by the iKIR interaction with the most protective genotype (*DQ6*) using data from the UK-GRID cohort and the prevalence of T1D in Europe [22]. If the population all carried a high number of functional iKIR (i.e. iKIR score > 1.75) then we estimate that the cases prevented by *DQ6* would be 21.6% but if the population all had a low number of functional iKIR (i.e. iKIR score ≤ 1.75) then 31.6% of cases would be prevented, an increase of more than 45%. To put this iKIR effect in context of other T1D-associated SNPs we normalized the iKIR score so it was on a scale of [0,1] (in line with presence/absence of a variant SNP) and then considered it as a main effect in a *DQ6+* cohort. The OR for iKIR score was 6.12 with a 95% confidence interval (CI) of 1.96 to 19.69 (and OR = 7.55, 95%CI 1.61–37.21 in the smaller cohort with individuals carrying HLA class I drivers removed). The more conservative value is plotted alongside the OR of other variants which have previously been associated with T1D for comparison (**Fig 5**).

### Mathematical model of the β-cell autoimmune destruction can recapitulate the iKIR score effect in the presence of saturation

**Fig 6:**
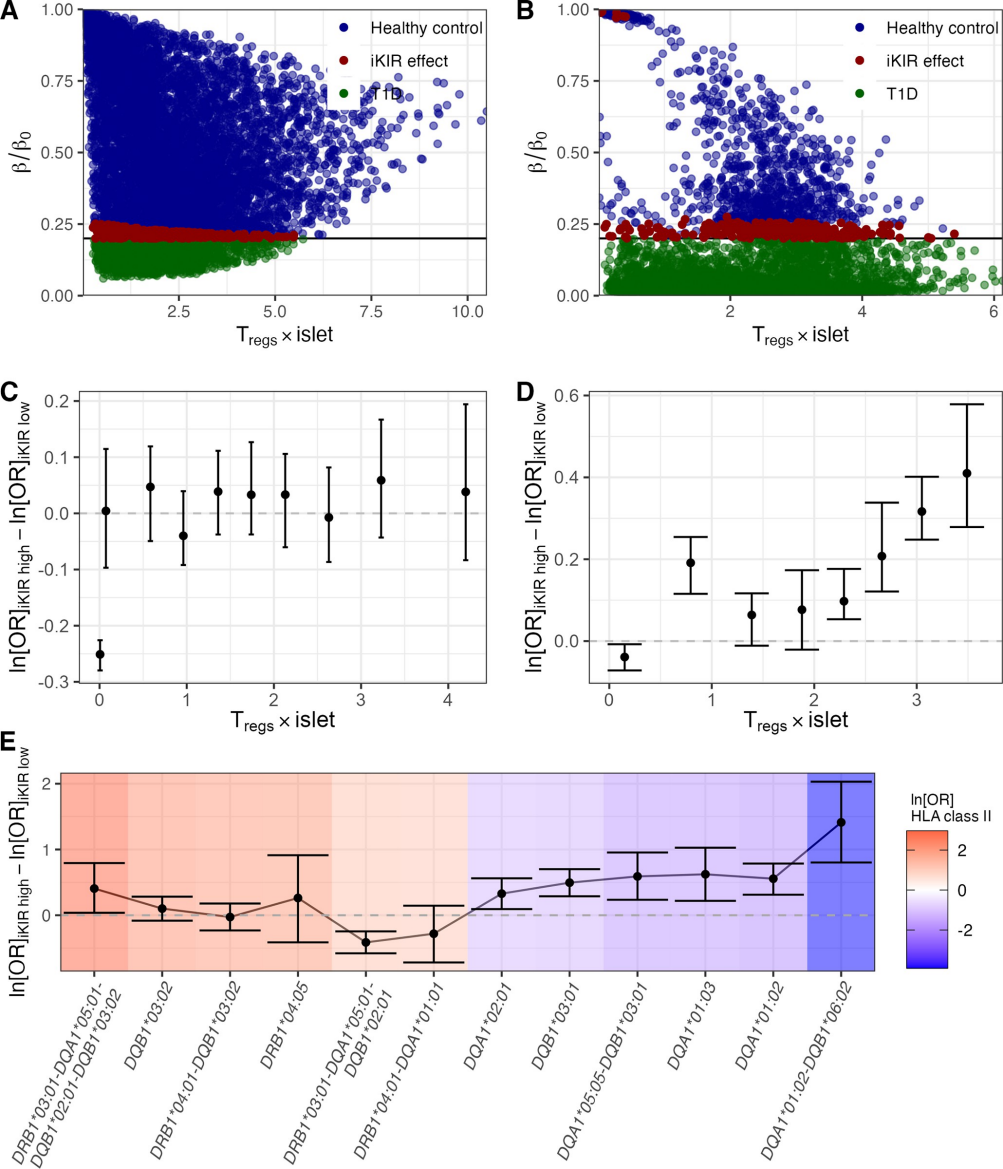
Mathematical model. Simulations of iKIR effect on the T cell response against insulin-producing β-cells. **A** and **B** Fraction of β-cell mass remaining as a function of the number of Tregs during the immune response is shown for each in silico individual in the simulated cohort with model 1 (**A**) and model 2 (**B**). Color code indicates outcome of the immune response: individuals that remain healthy after the immune response are shown in blue, those that transition to T1D are shown in green and individuals that have a different outcome depending on their functional iKIR gene count are shown in red. Note that the outcome depends on the threshold of disease onset. **C** and **D** Difference of OR for a cohort with high number vs a cohort with low number of functional iKIR genes as a function of number of Tregs during the immune response simulated with model 1 (**C**) and model 2 (**D**). **E** Difference between the ln[OR] of HLA class II genotypes in the group of UK-GRID individuals with a high iKIR score and the group with a low iKIR score. Color code according to the ln[OR] of the HLA class II genotype (risk).

If iKIRs increase CD4^+^ T cell lifespan (either directly via iKIR expression on the affected T cell or indirectly via iKIR expression on NK cells), as we have reported for CD8^+^ T cells [7], one might hypothesize that an iKIR-mediated enhancement of autoreactive T cell survival is detrimental, which agrees with our immunogenetic findings on protective HLA genotypes. However, by this reasoning, one would expect that the risk conferred by detrimental HLA genotypes like DRB1*04:01-DQB1*03:*02* would also be modulated by iKIR score, i.e., higher risk in individuals carrying detrimental HLA genotypes and high number of iKIR genes. To investigate this apparent discrepancy in the immunogenetic results we use mathematical modelling. Briefly, we implemented an ordinary differential equation system that reflects the interactions between the T cells and insulin producing β-cells in the pancreatic islet based on an existing model of the human antitumor T cell response [23]. We implemented two versions of this model, with and without density-dependent T cell production (see **S1 Materials and methods**) and generated a cohort of 10,000 in silico individuals, each one carrying a parameter combination randomly sampled from parameter ranges obtained from the literature (**S15 Table**). For each of the two models and for each parameter set, we run the simulation twice to describe the positive effect of iKIR on T cell survival [10,24]; the first simulation reflected individuals with high iKIR (i.e. they have lower T cell death rates) whilst the second reflected individuals with low iKIR (higher T cell death rates). In both models, increasing T cell survival resulted in progression to T1D in a small fraction of in silico individuals who would otherwise have been healthy but for the majority of simulations the outcome (health or development of T1D) was independent of iKIR (**Fig 6A and 6B**). We then asked whether an increased T cell survival is associated with higher T1D risk in carriers of protective but not neutral nor detrimental HLA genotypes. We assumed that HLA class II-protected in silico individuals have high number of islet-specific regulatory T cells (Tregs), as reported recently in a study on healthy individuals [25]. We classified individuals into groups on the basis of mean Treg numbers during the simulation and then computed the difference in ln[OR]s between the iKIR high and iKIR low in silico cohorts in each group. In model 1 (without density dependent T cell production), the difference of ln[OR]s remained constant for different groups with different numbers of Tregs (**Fig 6C**). However, in model 2 (with density dependent T cell production), the difference in ln[OR]s increases as the number of Tregs per islet increases (**Fig 6D**), which recapitulates the trend observed in the actual data (**Fig 6E)** and provides a possible explanation to our seemingly contradictory immunogenetic results. When Treg levels are saturated and reach carrying capacity –i.e., in carriers of protective HLA class II genotypes– the increase in T cell survival results in an increase of conventional T cell (Tconv) but not Treg population size. Consequently, in this scenario, there is an effective increase of β-cell destruction that cannot be compensated by Treg suppression of Tconvs. In unsaturated conditions though –i.e., in carriers of neutral or detrimental HLA genotypes –iKIR-mediated increase of both Treg and Tconv survival results in a zero net effect on β-cell destruction. These results are consistent with our observations.

### KIR^+^ T cell frequency is not increased in T1D patients

**Fig 7:**
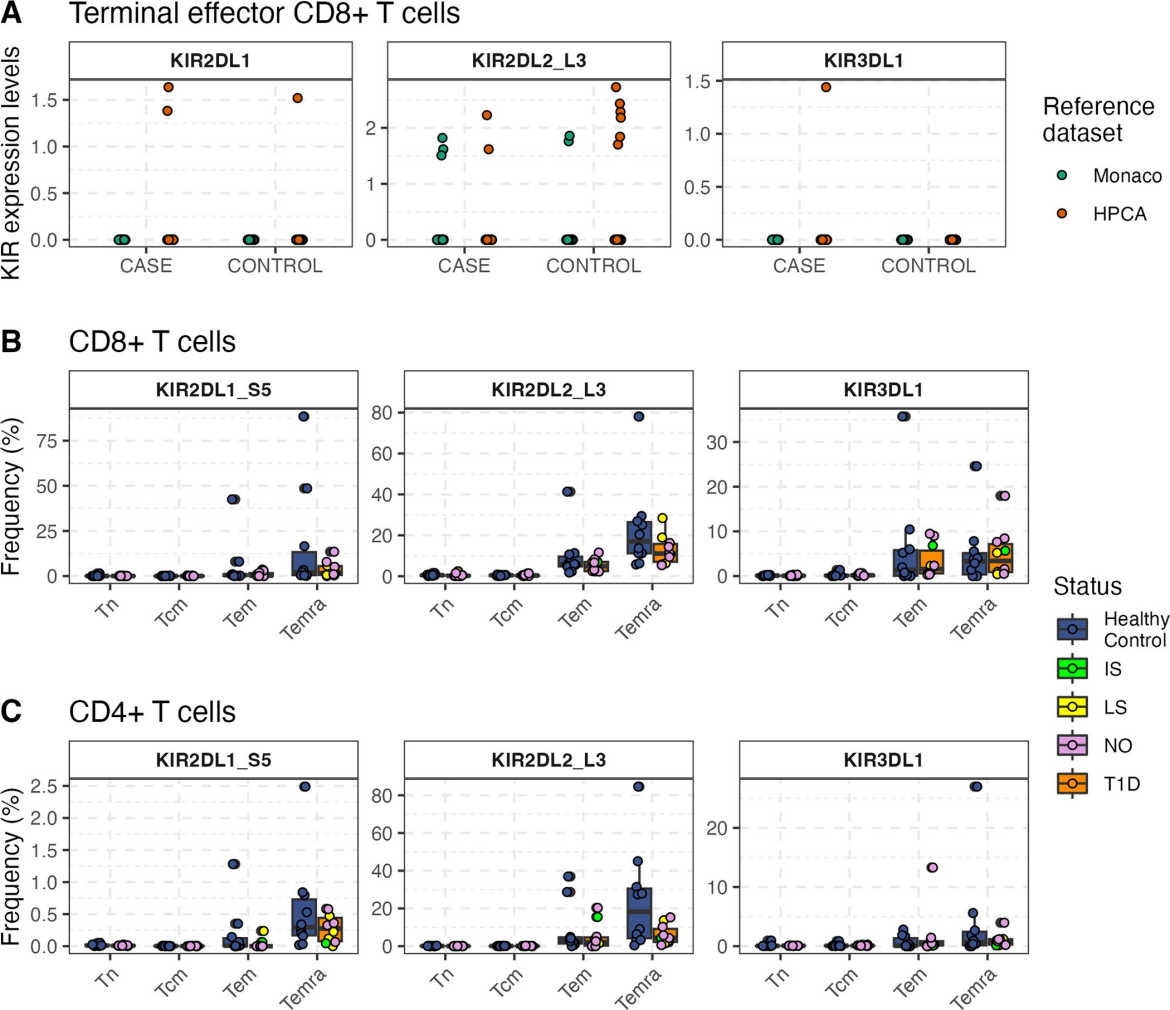
Frequency of terminal effector CD8^+^ T expressing KIR transcripts is similar between seropositive children and healthy controls. **A** Few CD8^+^ terminal effector memory cells express KIR transcripts. Cells labelled as *Terminal effector CD8 T cells* (Monaco reference, green) or as *T cell*:*CD8+ effector memory RA* (HPCA reference, orange) are shown split by disease status (case, control) and by KIR gene (*KIR2DL1*, *KIR2DL2/L3* and *KIR3DL1*). **B** The percentage of cells in each CD8^+^ T cell population (Tnaive, Tem, Tcm, Temra) expressing iKIRs quantified by flow cytometry. **C** The percentage of cells in each CD4^+^ T cell population (Tnaive, Tem, Tcm, Temra) expressing iKIRs quantified by flow cytometry. **B, C** Dots represent frequencies for each individual in the cohort. T1D samples are colour coded according to disease duration at time of collection (NO = new onset, IS = intermediate standing disease, LS = long standing disease). Boxes show medians and interquartile ranges within T1D individuals (N = 10, orange, irrespective of disease duration) and healthy individuals (N = 10, blue).

There is experimental evidence showing that iKIRs impact T cell responses via two main pathways. Directly, the ligation of iKIRs enhances T cell survival in vitro [8,10]. Indirectly, NK cells also modulate T cell lifespan by regulating activated T cell numbers. Recent work supports the latter pathway as being the most relevant in healthy and virus-infected individuals [7]. We wanted to investigate whether this was also the case in T1D. We hypothesized that if the functional iKIR effect on HLA class II genotypes is caused by the expression of iKIRs on T cells (direct pathway), we would expect to see differential expression of iKIRs on T cells between T1D patients and healthy controls. To test this hypothesis, we analysed scRNAseq data from PBMCs samples of 4 children with islet auto-antibodies (two of them developed T1D by 36 months of age) and 4 matched controls (see **Materials and methods**). Only two barcodes labelled as terminal effector CD4^+^ T cells were positive for *KIR* transcripts and none of the cells labelled as Tregs expressed KIRs. As expected, a greater proportion of terminally differentiated CD8^+^ T cells expressed KIRs (**Fig 7A**). Nevertheless, we detected *KIR* transcripts in CD8^+^ T cells from both seropositive individuals and healthy controls, suggesting that KIR expression in blood is not altered in disease. To validate those findings, we recruited 10 T1D patients (including new onset T1D patients and individuals with long standing disease) and 10 matched healthy controls and performed KIR immunophenotyping of CD4^+^, CD8^+^ and NK cell subsets by flow cytometry (see **Materials and methods**). We found that KIR protein expression was higher in late stage differentiated T cells and for KIR2DL2/L3 compared to KIR2DL1, in agreement with previous findings [7]. As in the scRNAseq analysis, we did not observe differences in KIR expression between T1D cases and controls within naïve or memory subsets (**Fig 7B**). As expected, KIR^+^ CD4^+^ T cells were rare, and frequencies were again comparable between cases and controls (**Fig 7C**). In summary, the frequency of KIR^+^ T cells is not altered through T1D disease stages, which argues against a direct effect of KIRs on T cells as the underlying mechanism of the iKIR gene modulation on HLA associations, consistent with our previous findings. We suggest, in line with the evidence for CD8^+^ T cells [7], that iKIRs enhance CD4^+^ T cell survival via the indirect pathway.

## Discussion

Our aim was to conduct an immunogenetic analysis to study the role of iKIRs in autoimmunity. Specifically, we wanted to investigate whether functional iKIR genes have a significant impact on HLA associations in T1D. This interaction is clinically significant in other contexts; we previously reported that functional iKIR genes enhanced protective and detrimental HLA disease associations in chronic viral infections. We postulated that a similar effect might be relevant in autoimmunity. We have analyzed a large (N = 11,961) case-control T1D dataset to investigate the effect of functional iKIR genes on HLA class II genetic associations. We found that a low number of functional iKIR genes (iKIR genes together with the genes encoding their corresponding HLA class I ligands), enhanced the dominant protection conferred by DQA1*01:02-DQB1*06:*02*. The effect was driven by iKIR-ligand pairs rather than HLA ligands alone, which are independently associated with T1D. The same results were observed for the other 9 protective HLA class II genotypes in our cohort; for all but two infrequent genotypes, the results were statistically significant and the effect was observed at all iKIR stratifications considered. The odds of observing this effect across all protective genotypes by chance is low (P = 2×10^−8^). Moving onto an independent replication cohort, an identical result was observed with protective class II genotypes being more protective in individuals with a low number of functional iKIR genes. Again, the probability of observing this result by chance was low (P = 1×10^−5^). In striking contrast, functional iKIR had no consistent impact on detrimental class II associations. Most detrimental genotypes showed no iKIR modification at all. Two detrimental genotypes which were iKIR modified were modified in opposite directions and on closer examination this was found to be attributable to linkage disequilibrium with a class I genotype in one case and inverse correlation with a protective class II genotype in the other case. In short, whilst the functional iKIR modification of protective HLA class II associations is very clear and highly statistically significant (UK-GRID cohort: P = 2×10^−8^, replication cohort: P = 1×10^−5^), the absence of an iKIR modification of detrimental genotypes is equally clear (P = 0.46).

We studied the underlying mechanism using single-cell RNA sequencing data, protein expression data and mathematical modelling. Evidence from ours and others studies indicate that iKIR ligation increases T cell lifespan. Recently we have shown that, for CD8^+^ T cells, iKIR expression by a third cell (other than the CD8^+^ T cell whose lifespan is extended) is necessary and that iKIR expression by the CD8^+^ T cell of interest is not relevant. If iKIR expression on T cells did explain our observations in T1D then iKIR gene expression on T cells might be expected to differ between T1D patients and healthy individuals. We did not find major differences in the size of KIR^+^ immune populations between cases and controls, suggesting, as hypothesized, that the direct ligation of iKIRs on T cells is not the main underlying mechanism of the functional iKIR gene effect. This is consistent with our recent work which also effectively rules out direct expression of iKIRs on CD8^+^ T cells as a mechanism for those T cells with increased survival [7]. Finally, we used a simple mathematical model of the immune destruction of β-cells to generate plausible hypotheses about the iKIR modulation of protective but not neutral nor detrimental HLA associations. We predicted that an increase of T cell survival–driven by a high number of functional iKIR genes–would have a detrimental effect only when regulatory T cells are present at saturation levels, corresponding to individuals with a protective HLA class II genotype. When T cell numbers are far from the T cell population carrying capacity, a longer T cell lifespan has a zero net effect on β-cell destruction; a change in conventional T cell numbers is compensated by a change in regulatory T cell numbers.

Although HLA class II associations with T1D are very well studied, to the best of our knowledge there are no reports of how these associations are modified by functional iKIR genes. There are several studies investigating iKIR genes and/or functional iKIR genes in T1D [13–15,26–28] and a very large number of studies investigating HLA class II associations (e.g. [1,3,29]) but none reporting the interaction. Although the absence of evidence for an interaction between functional iKIR genes and HLA class II genes in candidate gene studies can easily be explained by the argument that no one was motivated to study this particular three-way genetic association, it might be wondered why it was not picked up in one of the very large “catch-all” genome wide association studies (GWAS) performed in T1D [29–31]. The reason is that three gene associations of the type we report (KIR-ligand-class II) are never studied in GWAS as the explosion in the number of multiple comparisons for comparing all triplets of variants is prohibitive and so it is to be expected that the functional iKIR modification of HLA class II associations which we report would not be found by GWAS. Whilst we are not aware of studies of functional iKIR modifications of HLA associations in T1D, we have previously reported functional iKIR modification of protective and detrimental HLA class I associations in three chronic viral infections: human immunodeficiency virus type 1 (HIV-1), hepatitis C virus and human T cell leukemia virus type 1 (HTLV-1) [10,12].

There are interesting parallels between these previous studies in chronic viral infection and this current study in T1D in that all show highly significant functional iKIR modification of protective HLA associations that cannot be explained just by KIR or just by class I ligands. However, in both HIV-1 and HTLV-1 infection, detrimental HLA class I genotypes (HLA-B*35 and HLA-B*54 respectively) were significantly modified [10] whereas in T1D there was no evidence for functional iKIR modification of detrimental genotypes. Using mathematical modelling, we found a possible explanation for the preferential iKIR modification of protective HLA associations in T1D. If we assume that the most detrimental compound genotype DRB1*03:01-DQA1*05:01-DQB1*02:01-DQB1*03:*02* fails to produce the necessary islet-specific Tregs whereas the dominant protective genotype DQA1*01:02-DQB1*06:*02* drives Treg levels close to carrying capacity, then the iKIR effect is only manifest in the highly protective end of the spectrum of HLA class II associations. In summary, even when assuming that protective and detrimental HLA class II associations in T1D affect a common pathway, Treg numbers, we show that there is a possible mechanism that can recapitulate our observations in the data.

Broadly speaking there are two (non-exclusive) ways in which iKIR could affect class II-restricted CD4^+^ T cells: either by iKIR expression directly on CD4^+^ T cells or indirectly by iKIR expression on another population (that interacts with APCs expressing class II or CD4^+^ T cells restricted by class II). Here we discuss these two possibilities in turn starting with the “direct” hypothesis. Ligation of iKIRs expressed on the surface of T cells leads to phosphorylation of ITIMs in their cytoplasmic tail which recruits phosphatases including SHP1 leading to inhibition of TCR signalling which in turn can decrease effector function including cytokine production [32] and regulation [33] or modulate differentiation [34]; iKIRs on T cells have also been shown in vitro and in murine models to prolong CD8^+^ and CD4^+^ T cell lifetime [8,10,24,35,36]. Qin et al have reported that KIR3DL1 expression on Tregs negatively regulates Treg function in the NOD mouse and promotes T1D [33] which could be a plausible mechanism underlying our immunogenetic findings. Furthermore, in ankylosing spondylitis, cumulative evidence indicates ligation of KIR3DL2 on Th17 CD4^+^ T cells may promote their accumulation and survival [9,37,38]. Finally, there are interesting parallels with other inhibitors of T cell signalling including lymphoid protein tyrosine phosphatase and PD-1. Lymphoid protein tyrosine phosphatase (LYP) is a phosphatase which, like the iKIR, negatively regulates T cell receptor signalling. A SNP (1858 C->T) in *PTPN22*, which encodes LYP, is significantly associated with T1D. The disease associated variant is associated with stronger T cell inhibition [39,40] and Tregs from donors homozygous for the variant have decreased ability to regulate other T cells compared to Tregs from a donor homozygous for the major allele [41]. Analogously, we find greatest disease risk amongst individuals with a high number of functional iKIR genes. Similarly, PD-1 is an inhibitory receptor expressed by T cells with a similar downstream signalling pathway to iKIR, and is known to play a role in peripheral tolerance and regulation of autoimmunity [42]. Perhaps the strongest argument against a direct effect of iKIRs on CD4^+^ T cells is the extremely low numbers of CD4^+^ T cells expressing iKIRs. In healthy homeostasis only about 0.1–1% of memory CD4^+^ T cells express iKIR. It is hard to see how such a small population could have such a large biological effect. Furthermore, we found that iKIR expression on CD4^+^ cells was not increased in patients with T1D. This finding is in contrast with other autoimmune diseases. For example, iKIR expression by CD4^+^ T cells is increased in ankylosing spondylitis (1–6% of all CD4^+^ cells are KIR3DL2+, rising to 10–60% of Th17 CD4^+^ cells [9]) and other autoimmune diseases such as lupus show similar increases in iKIR expression [43]. A more recent study reports increased KIR^+^ T cell frequencies not only in lupus patients but also in individuals with multiple sclerosis and coeliac disease [44]. In T1D, we found that both KIR gene expression and protein expression is rather similar between cases and controls. This is true for both individuals with long standing and recent onset disease as well as for individuals without clinical diagnosis but positive for islet auto-antibodies.

The alternative, “indirect” hypothesis posits that iKIRs on another cell population indirectly modulate CD4^+^ T cells restricted by protective class II molecules. Probably the most likely contender for such a population is NK cells as they express high levels of iKIR and are known to interact with APCs and T cells both of which could lead to a modulation of class II-restricted CD4^+^ cell responses. NK cells kill autologous, activated but not resting CD4^+^ and CD8^+^ T cells [45,46]. *In vitro* experiments show that activated CD4^+^ T cells upregulate HLA-E, the ligand for the inhibitory receptor CD94/NKG2A to protect themselves from NK cell killing [47,48]. The same inhibitory receptor plays a role in the experimental autoimmune encephalomyelitis (EAE) mouse model for multiple sclerosis [49]. In this model, prevention of engagement of CD94/NKG2A, either by antibody-blockade or by knockin of a mutated ligand, resulted in elimination of autoreactive T cells and improvement of EAE. Furthermore, in studies of a murine T1D model, NOD mice immunized with Complete Freund’s adjuvant, NK cells decreased the numbers of autoreactive CD8^+^ T cells preventing T1D [50]. Similarly, NK cells can suppress CD8^+^ T cell-mediated hyperglycemia in a mouse model characterized by the transgenic expression of lymphocytic choriomeningitis virus on β-cells [51]. Perhaps, NK cells and Treg cells act in synergy to suppress autoreactive T cell responses and prevent autoimmunity. How functional iKIR modify HLA associations in T1D and whether similar modifications are seen in other autoimmune disease are exciting and important areas that merit further research.

One limitation of this work is that the iKIR-ligand binding groups which we use in our definition of “functional iKIR” are simplistic. Incomplete knowledge of how different KIR and HLA alleles and different (HLA bound) peptides affect KIR-HLA binding and signalling precludes a more sophisticated definition. Nevertheless, these simple groupings have proved very powerful in other studies [52–57]. With this definition of inhibitory score, we saw clear and reproducible results both in the T1D cohorts and in the HIV-1, HCV and HTLV-1 cohorts. This suggests that the inhibitory score is a meaningful metric. It is worth noting that in the majority of the analyses, the score is only used to split the cohorts in half, so second order changes to the calculation of the score will not necessarily change the results (because a person’s score can change considerably, and they will still remain in the same strata).

The importance of this work is twofold. First, we have identified a family of genes that significantly impact T1D risk. We estimate that, even if we only consider a single protective class II genotype, a population with a low number of functional iKIR would see more than a 45% increase in the fraction of cases prevented compared to a population with a high iKIR score. This constitutes one of the largest genetic risk factors for T1D reported in recent decades. Second, we find evidence that iKIRs have an impact on the T cell response in vivo. A number of in vitro and murine studies indicate that iKIRs can modulate T cell responses in autoimmunity; however, determining whether this has any relevance for human health has not been possible. Our results demonstrate that functional iKIRs have a biologically significant impact on class II-associated protection, most likely via an impact on class II-restricted protective T cell responses, which manifest as a clinically significant difference in the risk of developing T1D.

## Materials and methods

### Ethical approval

The immunogenetics component of this study was approved by the Imperial College Joint Research Compliance Office (Ref: 20IC6312). Written informed consent was obtained at the original study sites from all individuals and subjects from both UK-GRID and HBDI cohorts consented to the use of their data in future research studies into the genetic risks of diabetes. For the flow cytometry component (**Cohort for flow cytometry analysis**) all study procedures were conducted according to the principles of the Declaration of Helsinki and all participants gave written informed consent following protocols approved by the relevant ethics committee (NRES London 13/LO/0022, ICREC 21IC7146). Human samples used in this research project were obtained from the Imperial College Healthcare Tissue and Biobank (ICHTB). ICHTB is supported by the National Institute for Health Research (NIHR) Biomedical Research Centre based at Imperial College Healthcare NHS Trust and Imperial College London. ICHTB is approved by Wales REC3 to release human material for research (22/WA/0214).

### Immunogenetic cohorts

The UK-GRID cohort (N = 13,452) contains white European individuals from a UK-based case control study. The HBDI collection (Families = 342) is a multiplex family dataset comprising families with affected children. The cohorts and the individuals selected for downstream analysis are described in **S1 Materials and Methods**.

### Statistical analysis

The impact of genotype on disease status was assessed by multiple logistic regression to adjust for covariates. The effect of iKIR score on HLA associations was assessed by stratification and the associated p-values obtained by permutation test. Additional regression approaches and the family-based association analysis are described in **S1 Materials and Methods**.

### KIR expression analysis

#### KIR gene expression

A published single-cell RNA sequencing case-control dataset of children progressing to T1D was used to investigate KIR expression variation between cases and controls in NK and T cell populations in blood (EGA study accession: EGAS00001004070). Details on the scRNA-seq analysis can be found in **S1 Materials and Methods**.

#### KIR protein expression

To validate the findings obtained with the scRNA-seq dataset, 20 individuals were recruited (10 T1D cases and 10 controls) for KIR immunophenotyping by flow cytometry (**S1 Materials and Methods**).

## Supporting information

S1 Materials and methods Contains materials and methods used to perform immunogenetic analysis in the UK-GRID and HBDI cohorts, gene and protein expression analysis and mathematical modelling. (DOCX)

S1 Results Contains additional analysis on the iKIR score effect on *DQ6* protective association. (DOCX)

S1 Text Definition of functional iKIR and inhibitory score. (DOCX)

S1 Data Source data file. (XLSX)

S2 Data Supporting data file. (XLSX)

S1 Fig HLA and KIR imputation accuracy. **A** Imputation accuracy measures at each HLA loci. Prediction accuracy is computed as the number of correctly imputed alleles divided by the number of experimentally typed chromosomes. **B** Sensitivity and specificity values at *KIR3DL1*/*S1* locus. *KIR3DL1* CN was measured using two assay methods, one targeting *KIR3DL1* exon 4 (KIR3DL1ex4) and the other assay targeting exon 9 (KIR3DL1ex9).
(TIFF)

S2 Fig Functional iKIR do not consistently modify detrimental genotypes. **A** In the whole cohort, for most detrimental genotypes, iKIR have no impact on the detrimental effect (the ln[OR] of the detrimental genotype is very similar in the strata with a high iKIR score (blue) and the strata with a low iKIR score (orange) and the 95% confidence intervals are overlapping). Two genotypes (DRB1*03:01-DQA1*05:01-DQB1*02:*01* and DRB1*03:01-DQA1*05:01-DQB1*02:01-DQB1*03:*02*) show some evidence of iKIR modification but the modifications are in opposite directions. **B** The observed value of our test statistic (weighted mean of the difference in ln[OR] between the KIR high and the KIR low strata at threshold = 1.75), indicated by the red line, is entirely consistent with the distribution (grey histogram) of the same test statistic under the null hypothesis that the iKIR score has no impact on the detrimental genotypes (generated by permuting the iKIR score of individuals in the cohort). This indicates that the probability of obtaining our observation by chance is high (P = 0.46) and that there is no evidence to reject the null hypothesis of no iKIR modification. **C** Similarly in the cohort with HLA class I drivers removed. Most genotypes are not modified and where there is modification then results are in opposite directions. **D** In the cohort without HLA class I drivers the observed value of our test statistic (indicated by the red line), whilst still overlapping, is more of an outlier from the distribution (grey histogram) of the same test statistic under the null hypothesis that the iKIR score has no impact on the detrimental genotypes. This decrease in the test statistic is driven entirely by one genotype which is strongly iKIR modified (DRB1*03:01-DQA1*05:01-DQB1*02:*01*). Overall, there is some evidence for iKIR modification in this cohort (P **=** 0.04) but it is far from convincing. Examining the apparent iKIR modification of DRB1*03:01-DQA1*05:01-DQB1*02:*01* we found that it was explained by the negative correlation of DRB1*03:01-DQA1*05:01-DQB1*02:*01* with the protective genotype DQA1*01:02-DQB1*06:*02*, which is modified by functional iKIR score since, upon exclusion of DQB1*0602 carriers, DRB1*03:01-DQA1*05:01-DQB1*02:*01* was no longer iKIR modified (the converse was not the case i.e. DQA1*01:02-DQB1*06:*02* was still significantly modified by functional iKIR when DRB1*03:01-DQA1*05:01-DQB1*02:*01* carriers were excluded from the cohort*)*. In the corresponding permutation test the p-value becomes even less significant (P **=** 0.19). We conclude that there is no evidence that detrimental class II drivers are modified by functional iKIR.
(TIFF)

S3 Fig *DQ6* protection as a function of iKIR score. **A** *DQ6* protection increases (i.e. ln[OR] becomes more negative) as the iKIR score decreases. **B** This was true for all strata choices (definitions of high, intermediate, low) considered as shown in the table. Subjects were categorized as having low, intermediate (int) and high iKIR score using all category cutoffs that ensured more than 12 individuals in each group. Coefficients, p-values and group sizes are reported in **S2 Table**.
(TIFF)

S4 Fig Impact of iKIR score on 17 protective *DRB1-DQB1* haplotypes. **A** The protective effect of class II *DRB1-DQB1* haplotypes is enhanced in the group of individuals with an iKIR score equal to 1.75 or lower (iKIR score threshold = 1.75) with the exception of DRB1*07:01-DQB1*03:*03*. The number in the top right box corresponds to the odds of seeing this difference by chance (3×10^7^ permutations). The dot is the ln[OR] and the bars the 95% confidence intervals obtained from the regression, blue iKIR high strata, orange: iKIR low strata. **B** The observed value of our test statistic (weighted mean), indicated by the red arrow, lies far above the distribution (grey histogram) of the same test statistic under the null hypothesis that the iKIR score has no impact on the protective haplotypes (generated by permuting the iKIR score of individuals in the cohort, P<3×10^−7^).
(TIFF)

S5 Fig Simulation-based power analysis in the UK-GRID cohort. **A** For each sample size *s* (Number of cases = Number of controls = s/2), we generated 1000 random subcohorts by resampling with replacement individuals from the UK-GRID cohort. The sample size of the family dataset is 700 trios (indicated by a black line). For each protective genotype and each subcohort we run a logistic regression model with stratified iKIR score (threshold = 1.75) as an interaction term with the protective genotype. Power is estimated as the number of subcohorts where the coefficient of interaction was significant (P<0.05). The dashed red line indicates the power standard of 0.8. Given the sample size of the family dataset, the power to detect a significant iKIR score interaction on individual protective genotypes falls considerably below the recommendation of 0.8. **B** For each sample size, we estimated the power to detect significant difference between iKIR high and iKIR low strata (assessed by permutation test) across several protective genotypes simultaneously. Power is calculated as the proportion of cohorts with a significant permutation test. Given the sample size of the family dataset, the power of detecting an iKIR difference across 4 frequent protective genotypes i.e., DQA1*01:*02*, DQB1*03:*01*, DQA1*02:*01* and DQA1*01:02-DQB1*06:*02* is higher than the recommended standard of 0.8. Sample size family dataset (vertical black line), recommended power (horizontal dashed red line).
(TIFF)

S6 FigDRB1*15:01-DQB1*06:*02* associated protection is enhanced amongst individuals with low number of functional iKIR.Stratification analysis was repeated for different iKIR thresholds but this time including ligands as covariates in the model (OUTCOME∼DRB1*15:01−DQB1*06:02+GENDER+Bw4+C1+C2). The results are remarkably similar to our previous analysis on *DQ6* (see main text Fig 1). Estimates, p-values and cohort sizes are reported in **S8 Table**.(TIFF)

S7 Fig DRB1*15:01-DQB1*06:*02* associated protection as a function of iKIR score. **A** The ln[OR] of DRB1*15:01-DQB1*06:*02* decreases (i.e. becomes more protective) as the iKIR score decreases. **B** This was true for all strata choices (i.e. definitions of high, intermediate, low) considered as shown in the table (see also S9 Table). Subjects were categorized as having low, intermediate (int) and high iKIR score using different thresholds that ensured enough number of individuals in each group (at least N = 12). Here, ligands (Bw4, C1 and C2) were included in the model as covariates. These results for DRB1*15:01-DQB1*06:*02* are very similar to the results for DQA1*01:02-DQB1*06:*02* **(see S3 Fig).**
(TIFF)

S8 Fig iKIRs other than KIR3DL1 contribute to the iKIR score effect on *DQ6*. In a subcohort in which all individuals carry functional KIR3DL1 gene we still observe an enhanced protection of *DQ6* in individuals with low iKIR score. The number on the top right box corresponds to the odds of seeing this difference by chance (10^8^ permutations).
(TIFF)

S9 Fig Gating strategy for enumeration of iKIR^+^ CD8^+^ T cell subsets. Analysis of PBMCs for a healthy donor (LD1). Each subplot shows events in the parent population, where strip names indicate parent population (root = all events). **A** From left to right, serial gating is used to identify (1) lymphocytes and discard debris (root, all events), (2) single cells in the nonDebris gate, (3) CD3^+^ and Dump^−^cells in the singlet gate (excluding unwanted lineages like CD14, CD19 and also necrotic cells), (4) CD8^+^ T cells in the Dump^–^CD3^+^ gate and (5) naive (Tnaive), central memory (Tcm), effector memory (Tem) and effector memory RA^+^ (Temra) populations within the CD8^+^ gate using CD45RA and CD28 staining. **B** Each of the 4 subsets defined in the CD8^+^ gate (naive and memory subsets) was gated to determine events positive for KIR2DL1 (left), KIR2DL2/L3 (middle) and KIR3DL1 (left). In this case, only KIR gates within the Tcm population are shown but the same strategy is followed for Tem, Temra and Tnaive subsets. All boundaries are determined with the 1D mindensity function (except for singlets) using collapsed data across all individuals.
(PNG)

S10 Fig Gating strategy for enumeration of iKIR^+^ CD4^+^ T cell subsets. Analysis of PBMCs for a healthy donor (LD1). Each subplot shows events in the parent population, where strip names indicate parent population (root = all events). **A** From left to right, serial gating is used to identify (1) lymphocytes and discard debris (root, all events), (2) single cells in the nonDebris gate, (3) CD3^+^ and Dump^−^cells in the singlet gate (excluding unwanted lineages like CD14, CD19 and also necrotic cells), (4) CD4^+^ T cells in the Dump^–^CD3^+^ gate and (5) naive (Tnaive), central memory (Tcm), effector memory (Tem) and effector memory RA^+^ (Temra) populations within the CD4^+^ gate using CD45RA and CD28 staining. **B** Each of the 4 subsets defined in the CD4^+^ gate (naive and memory subsets) was gated to determine events positive for KIR2DL1 (left), KIR2DL2/L3 (middle) and KIR3DL1 (left). In this case, only KIR gates within the Tcm population are shown but the same strategy is followed for Tem, Temra and Tnaive subsets. All boundaries are determined with the 1D mindensity function (except for singlets) using collapsed data across all individuals.
(PNG)

S11 Fig Gating strategy to enumerate of iKIR^+^ NK cell subsets. Analysis of PBMCs for a healthy donor (LD1). Each subplot shows events in the parent population, where strip names indicate parent population (root = all events). **A** From left to right, serial gating is used to identify (1) lymphocytes, (2) single cells, (3) CD3- cells (CD3-Dump^–^), (4) NK cells using CD56 staining and (5) CD56^dim^CD16^+^ and CD56^bright^CD16^–^ populations using CD56 and CD16 staining. **B** CD56^dim^CD16^+^ and CD56^bright^CD16^–^ populations were gated to determine events positive for KIR2DL1 (left), KIR2DL2/L3 (middle) and KIR3DL1 (left). In this case, only KIR gates within the CD56^dim^CD16^+^ population are shown. All boundaries are determined with the 1D mindensity function (except for singlets) using collapsed data across all individuals.
(PNG)

S12 Fig KIR^+^ NK cells are not increased in T1D. **A** *KIR* gene expression in NK cells split by disease status (CASE = seropositive individuals, CONTROL = matched healthy individuals) and by reference dataset used for cell annotation (green = Monaco reference, orange = Human Primary Cell Atlas reference). Each dot indicates a single cell barcode. Cells labelled as *Natural killer cells* (Monaco reference) or *NK_cells*, *NK_cell*:*CD56hiCD62L*+, *NK_cell*:*IL2* (Human Primary Cell Atlas reference) are shown. **B** Percentage of NK cells expressing different iKIR in T1D patients and healthy controls. The percentage of KIR^+^ cells in each NK cell population (CD56^dim^CD16^+^ and CD56^bright^CD16^–^) was quantified by flow cytometry. Dots represent cell frequencies from parent population for each individual. T1D samples are colour coded according to disease duration at time of collection (NO = new onset, IS = intermediate standing disease, LS = long standing disease). Boxes show medians and interquartile ranges within T1D individuals (N = 10, orange, irrespective of disease duration) and healthy individuals (N = 10, blue).
(TIFF)

S1 Table *DQ6* protection in T1D is enhanced amongst individuals with a low iKIR score. The cohort was stratified into individuals with high or low iKIR score using different iKIR score thresholds (1.5, 1.75, 2.0 and 2.5). The protective effect of *DQ6* was evaluated independently in each stratum using multivariate logistic regression with gender included as a covariate. *DQ6* is significantly protective (ln[OR] = -3.74, P = 1.05×10^−157^) in the UK-GRID cohort (Group = Whole cohort, unstratified analysis). Regression coefficients, p-values and cohort sizes are reported for the different strata. P-value for the unstratified analysis calculated using Wald-test, all other p-values calculated using a permutation test.
(PDF)

S2 Table iKIR score impacts *DQ6* associated protection in a dose-dependent manner. Individuals were stratified into High, Intermediate and Low iKIR score categories using 6 different definitions of high, intermediate and low (i.e. 6 different strata choices). For all strata choices we observe the same picture: *DQ6* protection increases as iKIR score decreases.
(PDF)

S3 Table Impact of functional iKIR on 17 significantly protective *DRB1-DQB1* haplotypes. iKIR score effect on HLA class II mediated protection was assessed for 17 significantly protective phased haplotypes in our cohort by stratifying the cohort into individuals with a high iKIR score and individuals with a low iKIR score and the protective effect of the haplotype calculated separately in the two strata. Regression coefficients, 95% confidence intervals, p-values and counts in each stratum are reported for each haplotype. The protective effect of class II haplotypes is enhanced in the group of individuals with a low iKIR score (an iKIR score equal to 1.75 or lower) with the exception of DRB1*07:01-DQB1*03:*03*. The odds of seeing this difference by chance were assessed by permutation test for each haplotype (3×10^7^ permutations). Wald test p-values are reported for the unstratified analysis (Group = Whole cohort).
(PDF)

S4 Table The HLA class I and class II genotypes most closely identified with outcome in the UK-GRID cohort. This table shows the “driver” genotypes which were independently associated with outcome (see **Fig 3**). The natural log of the odds ratio (ln[OR]), associated p-value for each genotype and number of cases and controls carrying the genotype is shown. ln[OR] and p-values were obtained by multiple logistic regression in the whole cohort with gender as an additional covariate is reported (i.e. the coefficients and p-values are derived in a model containing one genotype at a time).
(PDF)

S5 Table iKIR score decreases protection associated with protective class II genotypes in T1D. The UK-GRID cohort without carriers of HLA class I drivers (N = 5,420) was stratified into individuals with high or low iKIR score at different cutoffs (1.5, 1.75 and 2.0). The protective effect of each protective genotype was evaluated independently in each stratum using multiple logistic regression with gender as covariate. HLA class II protection is enhanced in the iKIR low strata for all genotypes but for the very infrequent protective genotype DQA1*02:01-DQB1*03:*03*. Regression coefficients, permutation p-values and cohort sizes are reported for the different strata. P-value for the whole cohort (unstratified analysis) calculated using the Wald-test; p-values for the stratification analysis are calculated using the permutation test.
(PDF)

S6 TableiKIR score interaction terms with protective HLA class II drivers adjusted by relatedness in the cohort after exclusion of HLA class I drivers.After removal of class I drivers from the cohort (remaining cohort: N **=** 5,420), we modelled risk of T1D for each protective genotype and included iKIR score in the model as a continuous variable interacting with the HLA class II genotype (OUTCOME∼HLAclassIIgenotype×iKIR_score). To account for relatedness between individuals, we used a generalized mixed model including a genetic relatedness matrix as a random factor. The coefficient for the interaction term was in the expected direction for all genotypes and significant for 5 frequent genotypes. By comparison with **Table 2** (same analysis but without the genetic relatedness matrix) it can be seen that adjusting for cryptic relatedness had little impact (all coefficients and p values very similar, **Fig 4**).(PDF)

S7 Table Odds of observing an iKIR modification across DQA1*01:*02*, DQB1*03:*01*, DQA1*02:*01* and DQA1*01:02-DQB1*06:*02* in an independent cohort. The iKIR effect observed in the case-control cohort was validated in an independent family dataset. Trios were stratified into high (**>**threshold) and low (**≤** threshold) iKIR score according to the iKIR score of the child in each trio. For each threshold and each genotype, we calculated the ratio of transmitted to non-transmitted genes in each stratum. The odds of observing an equal or greater difference between log ratios across 4 frequent protective genotypes (DQA1*01:*02*, DQB1*03:*01*, DQA1*02:*01* and DQA1*01:02-DQB1*06:*02*) were assessed by permutation test.
(PDF)

S8 Table DRB1*15:01-DQB1*06:*02* protection in T1D is enhanced amongst individuals with a low iKIR score. The cohort was stratified into individuals with high or low iKIR score using different iKIR score thresholds (1.5, 1.75, 2.0 and 2.5). The protective effect of DRB1*15:01-DQB1*06:*02* was evaluated independently in each stratum using multivariate logistic regression with gender included as a covariate. DRB1*15:01-DQB1*06:*02* is significantly protective (ln[OR] = –3.9, P = 1.14×10^−165^) in the cohort (Group = Whole cohort, unstratified analysis). Regression coefficients, p-values and cohort sizes are reported for the different strata. P-value for the unstratified analysis calculated using Wald-test; all other p-values calculated using a permutation test.
(PDF)

S9 Table iKIR score impacts DRB1*15:01-DQB1*06:*02* protection in a dose-dependent manner. Individuals were stratified into High, Intermediate and Low iKIR score categories using 6 different definitions of high, intermediate, and low (i.e. 6 different strata choices). For all strata choices we observe the same picture, that DRB1*15:01-DQB1*06:*02* protection increases as iKIR score decreases as we observed for DQA1*01:02-DQB1*06:*02* (see **S3 Fig**).
(PDF)

S10 Table iKIR score effect on DR*15:01-DQ*06:*02* is independent of *DR3* and *DR4* haplotypes. *DR3* and *DR4* haplotypes were included as covariates and standardised iKIR score was included as an interaction term for comparison. We denote the strata *DR3* or *DR4* for ease of reference, but we are considering genes both in cis or in trans and only considering *DRB1* and *DQB1* genes.
(PDF)

S11 Table iKIR interaction remains significant in all HLA class I allele negative subcohorts. Individuals carrying a given HLA class I allele (Allele) are removed from the UK-GRID cohort and then the subcohort is modeled with iKIR as an interaction term with DRB1*15:01-DQB1*06:*02*. Coefficients (ln[OR]) and p-values for the interaction term are reported.
(PDF)

S12 Table iKIR score negatively impacts protection associated with *DQ6* in T1D even when iKIR ligands are included as covariates. The UK-GRID cohort was stratified into individuals with high or low iKIR score using different cutoffs (1.5, 1.75, 2.0 and 2.5). The protective effect of *DQ6* was evaluated independently in each stratum using multivariate logistic regression with gender, Bw4, C1 and C2 ligands included in the model as covariates. Overall conclusions were remarkably similar to our previous analysis (not including the ligands as covariates). Regression coefficients, permutation p-values and cohort sizes are reported for the different strata. P-value for the whole cohort (unstratified analysis) calculated using the Wald-test; p-values for the stratification analysis are calculated using the permutation test.
(PDF)

S13 Table iKIR interaction remains significant in all HLA class I allele negative subcohorts. Individuals carrying a given HLA class I allele (Allele) are removed from the UK-GRID cohort and then the risk of T1D is modeled with iKIR as an interaction term with *DQ6* in the allele-negative subcohort. Coefficients (ln[OR]) and p-values for the interaction term are reported.
(PDF)

S14 Table iKIR score effect on *DQ6* is independent of *DR3* and *DR4* detrimental genotypes. *DR3* (defined here to be DRB1*03:01-DQB1*02:*01 in cis* or *in trans*) and *DR4* (defined to be DRB1*04:01/02/04/05-DQB1*03:*02 in cis* or *in trans*) detrimental genotypes were included as covariates and standardised iKIR score was included as an interaction term for comparison.
(PDF)

S15 Table Parameters used in the mathematical model of β-cell destruction. (PDF)

S16 Table Flow cytometry panel. (PDF)

## References

[1] Noble JA, Valdes AM, Cook M, Klitz W, Thomson G, Erlich HA. The role of HLA class II genes in insulin-dependent diabetes mellitus: molecular analysis of 180 Caucasian, multiplex families. Am J Hum Genet. 1996;59(5):1134–48. 8900244 PMC1914851

[2] Clayton DG. Prediction and interaction in complex disease genetics: experience in type 1 diabetes. PLoS Genet. 2009;5(7):e1000540. doi: 10.1371/journal.pgen.1000540 19584936 PMC2703795

[3] Erlich H, Valdes AM, Noble J, Carlson JA, Varney M, Concannon P, et al. HLA DR-DQ haplotypes and genotypes and type 1 diabetes risk: analysis of the type 1 diabetes genetics consortium families. Diabetes. 2008;57(4):1084–92. doi: 10.2337/db07-1331 18252895 PMC4103420

[4] Wagtmann N, Rajagopalan S, Winter CC, Peruzzi M, Long EO. Killer cell inhibitory receptors specific for HLA-C and HLA-B identified by direct binding and by functional transfer. Immunity. 1995;3(6):801–9. doi: 10.1016/1074-7613(95)90069-1 8777725

[5] Crouse J, Xu HC, Lang PA, Oxenius A. NK cells regulating T cell responses: mechanisms and outcome. Trends Immunol. 2015;36(1):49–58. doi: 10.1016/j.it.2014.11.001 25432489

[6] Mora-Bitria L, Asquith B. Innate receptors modulating adaptive T cell responses: KIR-HLA interactions and T cell-mediated control of chronic viral infections. Immunogenetics. 2023;75(3):269–82. doi: 10.1007/s00251-023-01293-w 36719466 PMC9887252

[7] Zhang Y, Yan AW, Boelen L, Hadcocks L, Salam A, Gispert DP, et al. KIR-HLA interactions extend human CD8+ T cell lifespan in vivo. The Journal of clinical investigation. 2023;133(12). doi: 10.1172/JCI169496 37071474 PMC10266773

[8] Ugolini S, Arpin C, Anfossi N, Walzer T, Cambiaggi A, Forster R, et al. Involvement of inhibitory NKRs in the survival of a subset of memory-phenotype CD8+ T cells. Nature immunology. 2001;2(5):430–5. doi: 10.1038/87740 11323697

[9] Bowness P, Ridley A, Shaw J, Chan AT, Wong-Baeza I, Fleming M, et al. Th17 cells expressing KIR3DL2+ and responsive to HLA-B27 homodimers are increased in ankylosing spondylitis. J Immunol. 2011;186(4):2672–80. doi: 10.4049/jimmunol.1002653 21248258 PMC3210561

[10] Boelen L, Debebe B, Silveira M, Salam A, Makinde J, Roberts CH, et al. Inhibitory killer cell immunoglobulin-like receptors strengthen CD8(+) T cell-mediated control of HIV-1, HCV, and HTLV-1. Sci Immunol. 2018;3(29). doi: 10.1126/sciimmunol.aao2892 30413420 PMC6277004

[11] Mora-Bitria L, Asquith B. Germline natural killer cell receptors modulating the T cell response. Front Immunol. 2024;15:1477991. doi: 10.3389/fimmu.2024.1477991 39559364 PMC11570266

[12] Seich Al Basatena NK, Macnamara A, Vine AM, Thio CL, Astemborski J, Usuku K, et al. KIR2DL2 enhances protective and detrimental HLA class I-mediated immunity in chronic viral infection. PLoS pathogens. 2011;7(10):e1002270. doi: 10.1371/journal.ppat.1002270 22022261 PMC3192839

[13] Liu SL, Zheng AJ, Ding L. Association between KIR gene polymorphisms and type 1 diabetes mellitus (T1DM) susceptibility: A PRISMA-compliant meta-analysis. Medicine (Baltimore). 2017;96(52):e9439. doi: 10.1097/MD.0000000000009439 29384924 PMC6392676

[14] Shastry A, Sedimbi SK, Rajalingam R, Nikitina-Zake L, Rumba I, Wigzell H, et al. Combination of KIR 2DL2 and HLA-C1 (Asn 80) confers susceptibility to type 1 diabetes in Latvians. Int J Immunogenet. 2008;35(6):439–46. doi: 10.1111/j.1744-313X.2008.00804.x 19046302

[15] Middleton D, Halfpenny I, Meenagh A, Williams F, Sivula J, Tuomilehto-Wolf E. Investigation of KIR gene frequencies in type 1 diabetes mellitus. Hum Immunol. 2006;67(12):986–90. doi: 10.1016/j.humimm.2006.08.295 17174747

[16] van der Slik AR, Koeleman BP, Verduijn W, Bruining GJ, Roep BO, Giphart MJ. KIR in type 1 diabetes: disparate distribution of activating and inhibitory natural killer cell receptors in patients versus HLA-matched control subjects. Diabetes. 2003;52(10):2639–42. doi: 10.2337/diabetes.52.10.2639 14514651

[17] Noble JA, Valdes AM. Genetics of the HLA region in the prediction of type 1 diabetes. Current diabetes reports. 2011;11(6):533–42. doi: 10.1007/s11892-011-0223-x 21912932 PMC3233362

[18] Hoover ML, Marta RT. Molecular modelling of HLA-DQ suggests a mechanism of resistance in type 1 diabetes. Scand J Immunol. 1997;45(2):193–202. doi: 10.1046/j.1365-3083.1997.d01-389.x 9042432

[19] Erlich HA, Griffith RL, Bugawan TL, Ziegler R, Alper C, Eisenbarth G. Implication of specific DQB1 alleles in genetic susceptibility and resistance by identification of IDDM siblings with novel HLA-DQB1 allele and unusual DR2 and DR1 haplotypes. Diabetes. 1991;40(4):478–81. doi: 10.2337/diab.40.4.478 2010048

[20] Noble JA, Valdes AM, Varney MD, Carlson JA, Moonsamy P, Fear AL, et al. HLA class I and genetic susceptibility to type 1 diabetes: results from the Type 1 Diabetes Genetics Consortium. Diabetes. 2010;59(11):2972–9. doi: 10.2337/db10-0699 20798335 PMC2963558

[21] Yao Y, Ochoa A. Limitations of principal components in quantitative genetic association models for human studies. eLife. 2023;12. doi: 10.7554/eLife.79238 37140344 PMC10234632

[22] Mobasseri M, Shirmohammadi M, Amiri T, Vahed N, Hosseini Fard H, Ghojazadeh M. Prevalence and incidence of type 1 diabetes in the world: a systematic review and meta-analysis. Health Promot Perspect. 2020;10(2):98–115. doi: 10.34172/hpp.2020.18 32296622 PMC7146037

[23] Sontag ED. A Dynamic Model of Immune Responses to Antigen Presentation Predicts Different Regions of Tumor or Pathogen Elimination. Cell Syst. 2017;4(2):231–41 e11. doi: 10.1016/j.cels.2016.12.003 28131824 PMC5323365

[24] Ugolini S, Vivier E. Regulation of T cell function by NK cell receptors for classical MHC class I molecules. Current opinion in immunology. 2000;12(3):295–300. doi: 10.1016/s0952-7915(00)00090-x 10781402

[25] Wen X, Yang J, James E, Chow IT, Reijonen H, Kwok WW. Increased islet antigen-specific regulatory and effector CD4(+) T cells in healthy individuals with the type 1 diabetes-protective haplotype. Sci Immunol. 2020;5(44). doi: 10.1126/sciimmunol.aax8767 32060144 PMC7566980

[26] Zhi D, Sun C, Sedimbi SK, Luo F, Shen S, Sanjeevi CB. Killer cell immunoglobulin-like receptor along with HLA-C ligand genes are associated with type 1 diabetes in Chinese Han population. Diabetes Metab Res Rev. 2011;27(8):872–7. doi: 10.1002/dmrr.1264 22069276

[27] Sanjeevi S, Sun C, Kanungo A, Sanjeevi CB. Killer immunoglobulin receptor genes and their HLA-C ligand are associated with Type 1 diabetes in an Eastern Indian population. Diabet Med. 2016;33(1):91–6. doi: 10.1111/dme.12815 26031759

[28] Traherne JA, Jiang W, Valdes AM, Hollenbach JA, Jayaraman J, Lane JA, et al. KIR haplotypes are associated with late-onset type 1 diabetes in European-American families. Genes Immun. 2016;17(1):8–12. doi: 10.1038/gene.2015.44 26492518 PMC4746488

[29] Barrett JC, Clayton DG, Concannon P, Akolkar B, Cooper JD, Erlich HA, et al. Genome-wide association study and meta-analysis find that over 40 loci affect risk of type 1 diabetes. Nature genetics. 2009;41(6):703–7. doi: 10.1038/ng.381 19430480 PMC2889014

[30] Hakonarson H, Grant SF, Bradfield JP, Marchand L, Kim CE, Glessner JT, et al. A genome-wide association study identifies KIAA0350 as a type 1 diabetes gene. Nature. 2007;448(7153):591–4. doi: 10.1038/nature06010 17632545

[31] Burton PR, Clayton DG, Cardon LR, Craddock N, Deloukas P, Duncanson A, et al. Genome-wide association study of 14,000 cases of seven common diseases and 3,000 shared controls. Nature. 2007;447(7145):661–78. doi: 10.1038/nature05911 17554300 PMC2719288

[32] Henel G, Singh K, Cui D, Pryshchep S, Lee WW, Weyand CM, et al. Uncoupling of T-cell effector functions by inhibitory killer immunoglobulin-like receptors. Blood. 2006;107(11):4449–57. doi: 10.1182/blood-2005-06-2519 16469873 PMC1895796

[33] Qin H, Wang Z, Du W, Lee WH, Wu X, Riggs AD, et al. Killer cell Ig-like receptor (KIR) 3DL1 down-regulation enhances inhibition of type 1 diabetes by autoantigen-specific regulatory T cells. Proc Natl Acad Sci U S A. 2011;108(5):2016–21. doi: 10.1073/pnas.1019082108 21245333 PMC3033262

[34] Ridley A, Hatano H, Wong-Baeza I, Shaw J, Matthews KK, Al-Mossawi H, et al. Activation-Induced Killer Cell Immunoglobulin-like Receptor 3DL2 Binding to HLA–B27 Licenses Pathogenic T Cell Differentiation in Spondyloarthritis. Arthritis & Rheumatology. 2016;68(4):901–14. doi: 10.1002/art.39515 26841353 PMC4855641

[35] Gati A, Guerra N, Gaudin C, Da Rocha S, Escudier B, Lecluse Y, et al. CD158 receptor controls cytotoxic T-lymphocyte susceptibility to tumor-mediated activation-induced cell death by interfering with Fas signaling. Cancer Res. 2003;63(21):7475–82. 14612548

[36] Wong-Baeza I, Ridley A, Shaw J, Hatano H, Rysnik O, McHugh K, et al. KIR3DL2 binds to HLA-B27 dimers and free H chains more strongly than other HLA class I and promotes the expansion of T cells in ankylosing spondylitis. J Immunol. 2013;190(7):3216–24. doi: 10.4049/jimmunol.1202926 23440420 PMC3736094

[37] Chan AT, Kollnberger SD, Wedderburn LR, Bowness P. Expansion and enhanced survival of natural killer cells expressing the killer immunoglobulin-like receptor KIR3DL2 in spondylarthritis. Arthritis Rheum. 2005;52(11):3586–95. doi: 10.1002/art.21395 16255049

[38] Cauli A, Shaw J, Giles J, Hatano H, Rysnik O, Payeli S, et al. The arthritis-associated HLA-B*27:05 allele forms more cell surface B27 dimer and free heavy chain ligands for KIR3DL2 than HLA-B*27:09. Rheumatology (Oxford). 2013;52(11):1952–62. doi: 10.1093/rheumatology/ket219 23804219 PMC3798713

[39] Bottini N, Musumeci L, Alonso A, Rahmouni S, Nika K, Rostamkhani M, et al. A functional variant of lymphoid tyrosine phosphatase is associated with type I diabetes. Nature genetics. 2004;36(4):337–8. doi: 10.1038/ng1323 15004560

[40] Vang T, Congia M, Macis MD, Musumeci L, Orrú V, Zavattari P, et al. Autoimmune-associated lymphoid tyrosine phosphatase is a gain-of-function variant. Nature genetics. 2005;37(12):1317–9. doi: 10.1038/ng1673 16273109

[41] Vang T, Landskron J, Viken MK, Oberprieler N, Torgersen KM, Mustelin T, et al. The autoimmune-predisposing variant of lymphoid tyrosine phosphatase favors T helper 1 responses. Hum Immunol. 2013;74(5):574–85. doi: 10.1016/j.humimm.2012.12.017 23333624 PMC4011173

[42] Zamani MR, Aslani S, Salmaninejad A, Javan MR, Rezaei N. PD-1/PD-L and autoimmunity: A growing relationship. Cellular Immunology. 2016;310:27–41. doi: 10.1016/j.cellimm.2016.09.009 27660198

[43] Basu D, Liu Y, Wu A, Yarlagadda S, Gorelik GJ, Kaplan MJ, et al. Stimulatory and Inhibitory Killer Ig-Like Receptor Molecules Are Expressed and Functional on Lupus T Cells. The Journal of Immunology. 2009;183(5):3481. doi: 10.4049/jimmunol.0900034 19675166 PMC2730421

[44] Li J, Zaslavsky M, Su YP, Guo J, Sikora MJ, van Unen V, et al. KIR(+)CD8(+) T cells suppress pathogenic T cells and are active in autoimmune diseases and COVID-19. Science. 2022;376(6590):265–+. doi: 10.1126/science.abi9591 35258337 PMC8995031

[45] Waggoner SN, Cornberg M, Selin LK, Welsh RM. Natural killer cells act as rheostats modulating antiviral T cells. Nature. 2011;481(7381):394–8. doi: 10.1038/nature10624 22101430 PMC3539796

[46] Peppa D, Gill US, Reynolds G, Easom NJ, Pallett LJ, Schurich A, et al. Up-regulation of a death receptor renders antiviral T cells susceptible to NK cell-mediated deletion. The Journal of experimental medicine. 2013;210(1):99–114. doi: 10.1084/jem.20121172 23254287 PMC3549717

[47] Takao S, Ishikawa T, Yamashita K, Uchiyama T. The rapid induction of HLA-E is essential for the survival of antigen-activated naive CD4 T cells from attack by NK cells. J Immunol. 2010;185(10):6031–40. doi: 10.4049/jimmunol.1000176 20952676

[48] Nielsen N, Odum N, Urso B, Lanier LL, Spee P. Cytotoxicity of CD56(bright) NK cells towards autologous activated CD4+ T cells is mediated through NKG2D, LFA-1 and TRAIL and dampened via CD94/NKG2A. PLoS One. 2012;7(2):e31959. doi: 10.1371/journal.pone.0031959 22384114 PMC3284517

[49] Lu L, Ikizawa K, Hu D, Werneck MB, Wucherpfennig KW, Cantor H. Regulation of activated CD4+ T cells by NK cells via the Qa-1-NKG2A inhibitory pathway. Immunity. 2007;26(5):593–604. doi: 10.1016/j.immuni.2007.03.017 17509909 PMC3428267

[50] Lee IF, Qin H, Trudeau J, Dutz J, Tan R. Regulation of autoimmune diabetes by complete Freund’s adjuvant is mediated by NK cells. J Immunol. 2004;172(2):937–42. doi: 10.4049/jimmunol.172.2.937 14707066

[51] Lang PA, Crome SQ, Xu HC, Lang KS, Chapatte L, Deenick EK, et al. NK Cells Regulate CD8(+) T Cell Mediated Autoimmunity. Front Cell Infect Microbiol. 2020;10:36. doi: 10.3389/fcimb.2020.00036 32117809 PMC7031256

[52] Vince N, Bashirova AA, Lied A, Gao X, Dorrell L, McLaren PJ, et al. HLA class I and KIR genes do not protect against HIV type 1 infection in highly exposed uninfected individuals with hemophilia A. The Journal of infectious diseases. 2014;210(7):1047–51. doi: 10.1093/infdis/jiu214 24719475 PMC4215081

[53] Martin MP, Gao X, Lee J-H, Nelson GW, Detels R, Goedert JJ, et al. Epistatic interaction between KIR3DS1 and HLA-B delays the progression to AIDS. Nature genetics. 2002;31(4):429–34. doi: 10.1038/ng934 12134147

[54] Khakoo SI, Thio CL, Martin MP, Brooks CR, Gao X, Astemborski J, et al. HLA and NK Cell Inhibitory Receptor Genes in Resolving Hepatitis C Virus Infection. Science. 2004;305(5685):872–4. doi: 10.1126/science.1097670 15297676

[55] Ahlenstiel G, Martin MP, Gao X, Carrington M, Rehermann B. Distinct KIR/HLA compound genotypes affect the kinetics of human antiviral natural killer cell responses. The Journal of clinical investigation. 2008;118(3):1017–26. doi: 10.1172/JCI32400 18246204 PMC2214845

[56] Martin MP, Qi Y, Gao X, Yamada E, Martin JN, Pereyra F, et al. Innate partnership of HLA-B and KIR3DL1 subtypes against HIV-1. Nature genetics. 2007;39(6):733–40. doi: 10.1038/ng2035 17496894 PMC4135476

[57] Nakimuli A, Chazara O, Hiby SE, Farrell L, Tukwasibwe S, Jayaraman J, et al. A KIR B centromeric region present in Africans but not Europeans protects pregnant women from pre-eclampsia. Proc Natl Acad Sci U S A. 2015;112(3):845–50. doi: 10.1073/pnas.1413453112 25561558 PMC4311823

[58] Rich SS. Genetics and its potential to improve type 1 diabetes care. Curr Opin Endocrinol Diabetes Obes. 2017;24(4):279–84. doi: 10.1097/MED.0000000000000347 28509690 PMC6107086

