# Supplementary material for: Inhibitory KIRs decrease HLA class II-mediated protection in Type 1 Diabetes
Source: PLoS Genet. 2024 Dec 26;20(12):e1011456. doi: 10.1371/journal.pgen.1011456 (PMC11741628; doi:10.1371/journal.pgen.1011456)
Supplement: S1 Materials and methods — (DOCX) [file pgen.1011456.s001.docx]

## S1 Materials and methods

### Subjects

**The** **GRID case-control cohort.** Our primary cohort is a white European UK-based cohort of 13,452 individuals, with 6,783 cases from the Genetic Resource Investigating Diabetes (GRID) aged between 6 months and 16 years and 6,669 controls from the British 1958 Birth Cohort. Information on disease status was available for GRID individuals. Subjects were genotyped on the Illumina Infinium high density Immunochip array (Illumina Inc., USA). HLA and KIR type were imputed (see below). Individuals with missing information (either sex or HLA/KIR genotype) were discarded, leaving a total of 11,961 individuals (6,219 cases and 5,742 controls).
**The** **HBDI multiplex family dataset.** Our validation cohort consists of 402 US families from the Human Biological Data Interchange (HBDI), a repository founded for the purposes of diabetes research [1]. All families have at least 2 affected children and parents are not affected. All 1721 individuals in this repository have been previously genotyped at HLA and KIR genes as described in [2]. Pedigree information was available for all individuals. After removal due to missingness a total of 1700 individuals (342 families) were used in the analysis.

**Cohort for flow cytometry analysis**. We obtained KIR immunophenotype data from 10 T1D patients and 10 matched controls (matching factors: age and gender). Healthy controls age ranged between 22-65 with 60% females. T1D patients had long-standing disease (N=4), intermediate-standing disease (within 3 years of diagnosis, N=1) or were recently diagnosed (within 1 year of diagnosis, N=5). The T1D cohort age ranged between 18-58 with 70% females.

### HLA and KIR imputation

HLA alleles at classical HLA loci (*A, B, C, DRB1, DQA1, DQB1* and *DPB1*) were imputed to two field resolution with HIBAG v1.14.0 [3]. To ensure high imputation accuracy, call threshold (CT) was set to 0.5 (imputations with posterior probability below CT were not called and left blank). Call rates for classical HLA loci ranged between 92.75% and 99.67%. Imputed genotypes were compared with high-resolution experimentally typed HLA genotypes available in a subset of GRID samples. Prediction accuracy ranged from 86.68% to 98.06% (**S1A Fig**). For KIR imputation, haplotypes were estimated using SHAPEIT v.r837 [4] (parameters: 500 states, 10 burn-in iterations, 50 main iterations, 10 pruning iterations, genetic map HapMap phase II b37). KIR imputation was then performed using KIR*IMP [5] using 231 SNPs at chromosome 19. We kept imputations at a probability threshold of 0.5 which has been reported to ensure 92% accuracy. KIR copy number accuracy was assessed using copy number data on a subset of individuals directly typed by qPCR at *KIR3DL1/S1* locus [6] (**S1B Fig**).

### Haplotyping/HLA genotype phasing

Haplotypes at *DRB1* and *DQB1* loci were estimated from the imputed genotypes with the R package Haplo.stats [7]. Haplo.stats uses expectation-maximization algorithm with progressive insertion of loci to estimate the most probable pair of haplotypes for a given genotype. Haplotypes were phased separately in cases and controls.

### iKIR score and iKIR count

We define a *functional iKIR gene pair* as the presence of both the iKIR gene and the gene encoding its HLA ligand in the same individual [8]. For each individual in the cohort, we calculate the count of functional iKIR genes. We consider the following iKIRs: KIR2DL1, KIR2DL2/KIR2DL3 and KIR3DL1. We also compute the inhibitory score, this is the iKIR count adjusted to reflect the binding strength of the different iKIRs as reported in [8]; replicated here in the **S1 Text**. We obtain very similar results with both metrics unless stated otherwise in the text. When classifying individuals into “high” or “low” iKIR score strata, the threshold for calling high/low was chosen on purely pragmatic grounds to give balanced numbers in both arms (to minimise problems of sparse data). Multiple thresholds were tested when possible, and conclusions remained unchanged unless stated otherwise.

We also compute an absence of functional iKIR count to investigate whether HLA ligands alone are responsible for the iKIR score effect of HLA associations. This score reflects the number of genes for HLA ligands in the absence of their matching iKIR genes in an individual.

### Statistical analysis

#### Logistic Regression

Multiple logistic regression was used to investigate the effect of iKIRs on the significant HLA associations that we identified in our case-control cohort. We used two regression approaches to assess the impact of the genotype on disease outcome:

**Regression by stratification**. We stratified our cohort into individuals with high and low iKIR score (that is greater than the threshold and less than or equal to the threshold respectively) and then assessed the impact of HLA genotype on outcome using logistic regression within each iKIR stratum (two models). Significance was assessed by a permutation test (see below).

**Regression by interaction.** To explore the role of significant covariates in our cohort (HLA ligands and sex) on the iKIR effect, we modeled the whole cohort and included iKIR score as an interaction term with the HLA genotype of interest in the model $\boldsymbol{OUTCOME \sim HLA genotype\times iKIR\_score + COVARIATES}$.

Significance was given by the p-value of the interaction coefficient *HLAgenotype:iKIRscore*.

#### Permutation test

To assess the significance of the difference in odds ratio (OR) between the models in the two strata (high iKIR and low iKIR) for a single class II genotype, we conducted a permutation test using Monte Carlo methods. We permuted (by resampling without replacement) the iKIR score values across all the individuals, stratified the (permuted) cohort and run the models in the two strata. We repeated this process 10^8^ times. Then we counted the number of permutations where the ln[OR] in the iKIR_high strata was equal or greater than the observed ln[OR] in the iKIR_high strata and the ln[OR] in the iKIR_low strata was equal or less than the observed ln[OR] in the low strata. The P-value was the number of permutations with equal or more extreme values than the observed values divided by the total number of permutations.
Similarly, to obtain a P-value for the observed difference between strata across all protective HLA class II genotypes associated with T1D, we permuted the iKIR score, calculated the ln[OR] in the iKIR_high strata minus the ln[OR] in the iKIR_low strata and then calculated the mean of these absolute differences across the protective genotypes weighted by the number of genotype-positive T1D cases. We repeated the permutation 10^8^ times and counted the number of iterations where the weighted mean was more extreme than the observed weighted mean in our cohort; the P-value reported is this count of extreme values divided by the total number of permutations.

#### Identification of genotypes significantly associated with T1D

To identify genotypes significantly associated with risk of, or protection from, T1D we first identified all alleles at the *HLA-A, -B, -C, -DRB1, -DQA1* and *-DQB1* loci as well as all two and three allele genotypes at *DRB1*, *DQA1* and *DQB1* loci significantly associated with outcome. We considered alleles in cis and in trans as trans-acting effects are well-documented in T1D. At this stage we used a high P-value threshold for significance (P<5x10^-4^) to create an inclusive list. This yielded a list of 21,726 genotypes. Many of these genotypes are highly correlated and neutral genotypes could appear significant because they mark carriage of a causal “driver” genotype. To eliminate these neutral genotypes we asked, for each genotype on the list, whether its protective/detrimental effect was independent of all other genotypes on the list. Specifically, for each genotype we considered it sequentially paired with all other genotypes (a total of 21,696x21,695/2=235,347,360 pairs of genotypes) and assessed whether their protective or detrimental effects were independent of each other by including the two genotypes simultaneously in a multivariate regression and assessing whether i) the direction of their protective/detrimental effect remained unchanged and ii) whether the effect remained significant (P<5x10^-4^). For colinear genotypes we adopted the following rules: if the genotypes were completely colinear (one allele always present with the other) then we retained the longest genotype (as it is impossible to rule out the possibility that the longer genotype was the driving genotype). If the genotypes were weakly colinear we retained the genotype with the lowest P-value in a multiple regression. This yielded a list of 33 alleles or sets of alleles significantly associated with outcome whose effects were independent of all other single alleles or sets of alleles. We then manually curated the list removing two genotypes (*DRB1*07:01-DQA1*01:02* and *DQA1*01:02-DQB1*03:01*) that were compounds of other genotypes on the list and although were independent of each of these genotypes individually were not independent of both of them simultaneously (i.e. in multiple regression with all three genotypes including the eliminated genotype suffered a reversed direction of effect). A third genotype (*DRB1*03:01-DQA1*05:01-DQB1*02:01-DQB1*03:02*) was retained on the list since although it appears to be a compound of the detrimental haplotype *DRB1*03:01-DQA1*05:01-DQB1*02:01* and the detrimental genotype *DQB1*03:02* it retained direction and significance of effect (albeit considerably weakened) in multiple regression when both *DRB1*03:01-DQA1*05:01-DQB1*02:01* and *DQB1*03:02* were included simultaneously with it (presumably due to synergy between the genetic effects). Finally, (having used an inclusive list to make sure alleles were stringently removed due to lack of an independent effect) we then calculated the effective number of tests conducted (for all alleles at the *HLA-A, -B, -C, -DRB1, -DQA1* and *-DQB1* loci as well as all pairs and trios of *DRB1, DQA1* and *DQB1* alleles, taking into account that many of these tests are highly correlated). We considered four methods to estimate the number of effective tests: that of Nyholt, Li and Ji, Gao et al and Galwey [9-12] implemented in the function meff of the poolr package in R. All four methods gave similar values for M_eff_, we took the highest M_eff_ value (obtained with Li & Ji method) to obtain the most stringent cutoff, which via the Bonferroni correction (P=0.05/M_eff_) resulted in a cut off for significance of P=1.35x10^-5^.

#### Population structure adjustment using a generalized linear mixed model

The lme4qtl R package [13] was used to include a random effect or variance component that reflected genetic correlations, either due to ancestry and/or cryptic relatedness [14], between the 11,961 UK-GRID individuals. First, we used the full Immunochip SNP data from UK-GRID individuals to calculate a genetic relationship matrix (GRM) using the snpgdsGRM function from SNPRelate R package [15] with the method GCTA [16]. Before estimating the GRM, SNPs were pruned with snpgdsLDpruning function. All SNPs in chromosomes 6 and 19 were excluded. The relmatGlmer() function from the lme4qtl R package was used to fit a GLMM model with the relatedness information estimated in the GRM included as a random effect. The specified model was ***CASE ~ DQ6*iKIR_score+(1|ID)***, where ID is individual identifier (one for each individual) and is mapped to the GRM through the relmat argument (note that GRM is symmetric, with rownames=colnames=ID).

#### Power analysis

The independent US family dataset was used for validation. This dataset is ten times smaller than the GRID cohort (700 trios vs 6219 cases in the GRID cohort). Therefore, before attempting to validate our results, we used Monte Carlo methods to study the statistical power to detect a significant iKIR score modification given the sample size of our validation set. We assumed that the observed iKIR effect in the GRID cohort was representative of the effect size in the US cohort and used Monte Carlo methods to conduct a simulation-based power analysis. Briefly, we generate a bootstrap subsample (resampling individuals in our cohort with replacement) of the GRID cohort with size equal *s* (N_controls_=s/2, N_cases_=s/2) and we run the regression analysis on this subsample. This process is repeated 1000 times. The estimated power for sample size *s* is the proportion of bootstrap subsamples where we retrieved a significant difference across protective genotypes. In this way we assessed which genotypes or combinations of genotypes had sufficient power to permit study in the family dataset.

#### Family-based analysis

We analyzed each family as trios for each affected sibling. Trios were stratified into “KIR high” or “KIR low” based on the iKIR score of the affected child (KIR high: iKIRscore>threshold, KIR low: ≤threshold, thresholds considered: 1.75 in first instance as it yields most balanced division but 1, 1.5, 2 and 2.5 were also considered). Then for both strata and each driver allele, we counted the number of transmissions of the protective allele from heterozygous parents to affected children and the number of non-transmissions i.e. the transmissions of the protective allele to a non-person. Following Spielman et al and Ott [17, 18] we calculated b and c in the matrix:

|  |  | Not transmitted | |  |
| --- | --- | --- | --- | --- |
|  |  | *M_1_* | *M_2_* |  |
| transmitted | *M_1_* | a | b | a+b |
|  | *M_2_* | c | d | c+d |
|  |  | a+c | b+d | 2n |

Where M is the locus of interest, *M_1_* is the protective “driver” allele and *M_2_* all other alleles at that locus.

Our statistic was the difference of the log of the ratio of transmitted to non-transmitted genes between individuals with a high iKIR score and individuals with a low iKIR score i.e

$$\ln[b/c|_{KIR high}]-\ln[b/c|_{KIR low}]$$

Whilst Spielman and Ott calculated the distribution of the transmission ratio under their null hypotheses of interest analytically, this approach was not available to us as we were interested in several driver alleles whose transmission could not be guaranteed to be independent of each other. We therefore calculated the null distribution using a Monte Carlo approach. Specifically, the distribution of the statistic under the null hypothesis that iKIR score does not modulate HLA class II associations was assessed by permuting the iKIR score values among affected offspring and then calculating the statistic. This was repeated 10^5^ times. The empirical P-value was the proportion of permutations with an estimate equal or more extreme than the observed estimate.

### Fraction of cases prevented

Following [19] we define the fraction of cases prevented by a genotype *G^+^* as *F_P_*

$$F_{P}=\frac{P(D|G^{-})-P(D)}{P(D|G^{-})}$$

which, given the following contingency table

|  | *G^+^* | *G^-^* |
| --- | --- | --- |
| D (Disease) | a | b |
| H (Healthy) | c | d |

can be rewritten as

$$F_{p}=(1-R)\left(1-\frac{d(a+b)}{b(c+d)}\right)$$

where R is the prevalence of disease.

### Other genes associated with T1D

We generated a comprehensive list of T1D-associated loci reported to date in the NHGRI-EBI GWAS catalog. The NHGRI-EBI GWAS catalog is a manually curated collection of published GWASs that is regularly updated. Only SNP associations with P<1x10^-5^ from GWASs with more than 100,000 tagged SNPs (preQC) are included in the database.

T1D GWAS-associated SNPs reported in the GWAS catalog were downloaded on the 22nd March 2021 from https://www.ebi.ac.uk/gwas/efotraits/EFO_0001359. The search term *type 1 diabetes* returned a total of 351 associations from 56 different studies. We only kept SNP associations under the EFO trait *type I diabetes mellitus*. The resulting list contained 196 SNP associations from 16 different studies mapping 67 different genes. We only report protein coding genes with significant SNP associations (within the gene or nearby) that have been reported in at least 2 out of the 16 T1D GWAS publications (either the same SNP or different SNPs mapping to the same gene). The mapped genes and their effect sizes are reported together with date when they were first reported. The date of discovery was manually curated to account for the fact that some loci were first reported in candidate gene studies (before GWASs, e.g. HLA region or INS gene).

### Single-cell RNAseq analysis from peripheral blood mononuclear cell samples

Raw FASTQ files from UMI-based single-cell RNAseq experiment of peripheral blood mononuclear cells were downloaded from the European Genome-Phenome Archive (EGA) with accession number EGAS00001004070. The samples were collected from four children who developed β-cell autoimmunity (i.e. were seropositive for islet auto antibodies) and matched control subjects [20]. Reads were aligned using STARsolo aligner with the option -soloMultiMappers Uniform [21]. After read mapping and count quantification, the 8 count matrices were read into R using the Seurat package for quality control [22]. Briefly, empty barcodes, apoptotic or stressed cells and doublets were discarded leaving a total of 18,519 cells. An automated annotation method, SingleR [23], was then used to classify single cells. Given a reference dataset of single-cell or bulk samples with known labels, SingleR classifies new cells from a test dataset based on spearman correlation between the test and the reference. The Monaco and the human primary cell atlas (HPCA) reference datasets from the Celldex package were used [23].

To make feature counts comparable across cells, we used the NormalizeData() function from Seurat: feature counts are divided by the cell library size (total umi counts of the cell) and multiplied by 10,000. The resulting feature quantities are natural-log transformed using log1p. So KIR expression values are log normalized KIR counts per 10,000 reads.

### KIR protein expression analysis

20 individuals including 10 T1D patients and 10 matched controls were recruited for KIR immunophenotyping flow cytometry analysis. The healthy cohort age ranges between 22-65 with 60% females and the T1D cohort age ranges between 18-58 with 70% females. The multi-colour antibody panel (**S16 Table**) was designed to assess expression of KIR2DL1, KIR2DL2/L3 and KIR3DL1 in the different CD4+ and CD8+ naive and memory populations as well as in NK cell subsets.

**T cell subsets**

CD28 and CD45RA staining was used to gate CD4+ and CD8+ T cells into the following populations (see **Figs S9 and S10** for representative gating strategy):

- Tnaive: CD28+CD45RA+
- Tcm (central memory): CD28+CD45RA-
- Tem (effector memory): CD28-CD45RA-
- Temra (effector memory expressing CD45RA): CD28-CD45RA+

**NK cell subsets**

CD56 and CD16 staining was used to identify NK cells (see **S11 Fig** for representative gating strategy). First CD56 staining was used to identify NK cells in the CD3-Dump- gate. Next, both CD56 and CD16 markers were used to identify CD56dimCD16+ and CD56brightCD16- NK cell populations.

All samples were stained and analysed on a BD FACSAria III (BD Biosciences) in the same experiment. Automated flow cytometry data analysis was performed in R. singletGate method from OpenCyto package was applied to gate out singlets [24]. All remaining gates were based on the mindensity method. Gates were determined using collapsed data across all individuals. Independent manual gating by someone with expertise in flow cytometry gave very similar results.

### Mathematical model of the transition from health to T1D

We model autoimmune response against beta cells in the pancreatic islet with a set of four coupled differential equations describing the interactions between activated islet specific T cells, both conventional CD8+ (C) and regulatory CD4+ (R) T cells, insulin producing β-cells (B) and islet antigen levels (A):

$\frac{dB}{dt}=-\delta_{B}BC$ (1)

$\frac{dA}{dt}=\alpha_{A}BC-\delta_{a}A$ (2)

$\frac{dR}{dt}=\alpha_{R}A-\delta_{T}R$ (3)

$\frac{dC}{dt}=\alpha_{C}A-\delta_{T}C-\delta_{i}RC$ (4)

We assume that the presence of islet antigens (A) activates conventional T cells (C) and regulatory T cells (R). We assume that regulatory cells are CD4+, conventional cells could either be CD4+ or CD8+ T cells with effector function. The activation of C and R has two opposite effects; while C mediates β-cell killing, R reduces activated C levels (which in turn reduces β-cell killing). The coexistence of these two antagonistic pathways triggered by the same input constitutes a type of incoherent feedforward loop (IFFL). This motif has been used to describe immune activation and tumor control [25]. The IFFL is coupled to a positive feedback loop: T cell mediated killing of β-cells results in more islet antigen release that in turn activates T cells (C and R).

We explored additional structural forms of the model. We included density-dependent production of T cells and modelled the negative feedback loop via R inhibition on C proliferation:

$\frac{dB}{dt}=-\delta_{beta}BC$ (5)

$\frac{dA}{dt}=\alpha_{A}BC-\delta_{a}A$ (6)

$\frac{dR}{dt}=\alpha_{R}AR\left( 1-\frac{R}{K_{R}} \right)-\delta_{T}R$ (7)

$\frac{dC}{dt}=\frac{\alpha_{C}AC}{k+R}\left( 1-\frac{C}{K_{C}} \right)-\delta_{T}C$ (8)

We refer to the density-independent and the density-dependent models as model 1 and model 2 respectively. Parameters used for the simulations using each model are listed in **S15** **Table**.

### Simulations

We apply the cellular model described above to a virtual cohort of 10,000 individuals, each one defined by a different set of parameters. T cell parameters define the genetic risk of an individual to develop T1D. HLA genes only explain 50% of T1D risk [26] so not all individuals with a susceptible genotype will develop T1D and other genes and factors such as history of infections, microbiome and lifestyle are thought to contribute to the transition to T1D. To overcome these unknowns, we simulate a cohort of young seropositive individuals. This way we have a homogeneous population of individuals of similar age with an active adaptive response (triggered by an enteroviral infection for example). Even though seropositivity for islet antigens is associated with T1D, not all seropositive individuals progress to overt T1D [27]. Thus, depending on each individual parameters, the immune response will be resolved without significant β-cell loss or alternatively will lead to massive β-cell killing, resulting in T1D onset. We assume similar dynamics (on average) in all the pancreatic islets and that loss of 80% of the beta cells in the islet leads to T1D.

### Software

All calculations were performed using custom scripts in R (v4.0.3). Packages used include stats (v3.6.2), parallel (v4.0.3), data.table (v1.13.2), Haplo.stats (v1.8.6), poolr (v0.8-2) and debug (v1.3.83).

## References

1. Lernmark Å. Human cell lines from families available for diabetes research. Diabetologia. 1991;34(1):61-. doi: 10.1007/BF00404029.

2. Traherne JA, Jiang W, Valdes AM, Hollenbach JA, Jayaraman J, Lane JA, et al. KIR haplotypes are associated with late-onset type 1 diabetes in European-American families. Genes Immun. 2016;17(1):8-12. doi: 10.1038/gene.2015.44.

3. Zheng X, Shen J, Cox C, Wakefield JC, Ehm MG, Nelson MR, et al. HIBAG--HLA genotype imputation with attribute bagging. Pharmacogenomics J. 2014;14(2):192-200. doi: 10.1038/tpj.2013.18.

4. Delaneau O, Zagury JF, Marchini J. Improved whole-chromosome phasing for disease and population genetic studies. Nat Methods. 2013;10(1):5-6. doi: 10.1038/nmeth.2307.

5. Vukcevic D, Traherne JA, Naess S, Ellinghaus E, Kamatani Y, Dilthey A, et al. Imputation of KIR Types from SNP Variation Data. Am J Hum Genet. 2015;97(4):593-607. doi: 10.1016/j.ajhg.2015.09.005.

6. Pontikos N, Smyth DJ, Schuilenburg H, Howson JM, Walker NM, Burren OS, et al. A hybrid qPCR/SNP array approach allows cost efficient assessment of KIR gene copy numbers in large samples. BMC Genomics. 2014;15:274. doi: 10.1186/1471-2164-15-274.

7. Sinnwell JP, Schaid DJ. haplo.stats: Statistical Analysis of Haplotypes with Traits and Covariates when Linkage Phase is Ambiguous. 1.8.5 ed2020. p. Routines for the analysis of indirectly measured haplotypes. The statistical methods as-sume that all subjects are unrelated and that haplotypes are ambiguous (due to unknown link-age phase of the genetic markers). The main func-tions are: haplo.em(), haplo.glm(), haplo.score(), and haplo.power(); all of which have de-tailed examples in the vignette.

8. Boelen L, Debebe B, Silveira M, Salam A, Makinde J, Roberts CH, et al. Inhibitory killer cell immunoglobulin-like receptors strengthen CD8(+) T cell-mediated control of HIV-1, HCV, and HTLV-1. Sci Immunol. 2018;3(29). doi: 10.1126/sciimmunol.aao2892.

9. Nyholt DR. A simple correction for multiple testing for single-nucleotide polymorphisms in linkage disequilibrium with each other. American journal of human genetics. 2004;74(4):765-9. doi: 10.1086/383251.

10. Li J, Ji L. Adjusting multiple testing in multilocus analyses using the eigenvalues of a correlation matrix. Heredity. 2005;95(3):221-7. doi: 10.1038/sj.hdy.6800717.

11. Gao X, Starmer J, Martin ER. A multiple testing correction method for genetic association studies using correlated single nucleotide polymorphisms. Genetic epidemiology. 2008;32(4):361-9. doi: 10.1002/gepi.20310.

12. Galwey NW. A new measure of the effective number of tests, a practical tool for comparing families of non-independent significance tests. Genetic epidemiology. 2009;33(7):559-68. doi: 10.1002/gepi.20408.

13. Ziyatdinov A, Vazquez-Santiago M, Brunel H, Martinez-Perez A, Aschard H, Soria JM. lme4qtl: linear mixed models with flexible covariance structure for genetic studies of related individuals. BMC Bioinformatics. 2018;19(1):68. doi: 10.1186/s12859-018-2057-x.

14. Sul JH, Martin LS, Eskin E. Population structure in genetic studies: Confounding factors and mixed models. PLoS Genet. 2018;14(12):e1007309. doi: 10.1371/journal.pgen.1007309.

15. Zheng X, Levine D, Shen J, Gogarten SM, Laurie C, Weir BS. A high-performance computing toolset for relatedness and principal component analysis of SNP data. Bioinformatics. 2012;28(24):3326-8. doi: 10.1093/bioinformatics/bts606.

16. Yang J, Lee SH, Goddard ME, Visscher PM. GCTA: a tool for genome-wide complex trait analysis. Am J Hum Genet. 2011;88(1):76-82. doi: 10.1016/j.ajhg.2010.11.011.

17. Ott J. Statistical properties of the haplotype relative risk. Genetic epidemiology. 1989;6(1):127-30. doi: 10.1002/gepi.1370060124.

18. Spielman RS, McGinnis RE, Ewens WJ. Transmission test for linkage disequilibrium: the insulin gene region and insulin-dependent diabetes mellitus (IDDM). Am J Hum Genet. 1993;52(3):506-16. doi.

19. Miettinen OS. Proportion of disease caused or prevented by a given exposure, trait or intervention. American journal of epidemiology. 1974;99(5):325-32. doi: 10.1093/oxfordjournals.aje.a121617.

20. Kallionpaa H, Somani J, Tuomela S, Ullah U, de Albuquerque R, Lonnberg T, et al. Early Detection of Peripheral Blood Cell Signature in Children Developing beta-Cell Autoimmunity at a Young Age. Diabetes. 2019;68(10):2024-34. doi: 10.2337/db19-0287.

21. Kaminow B, Yunusov D, Dobin A. 2021. doi: 10.1101/2021.05.05.442755.

22. Hao Y, Hao S, Andersen-Nissen E, Mauck WM, 3rd, Zheng S, Butler A, et al. Integrated analysis of multimodal single-cell data. Cell. 2021;184(13):3573-87 e29. doi: 10.1016/j.cell.2021.04.048.

23. Aran D, Looney AP, Liu L, Wu E, Fong V, Hsu A, et al. Reference-based analysis of lung single-cell sequencing reveals a transitional profibrotic macrophage. Nature immunology. 2019;20(2):163-72. doi: 10.1038/s41590-018-0276-y.

24. Finak G, Frelinger J, Jiang W, Newell EW, Ramey J, Davis MM, et al. OpenCyto: an open source infrastructure for scalable, robust, reproducible, and automated, end-to-end flow cytometry data analysis. PLoS Comput Biol. 2014;10(8):e1003806. doi: 10.1371/journal.pcbi.1003806.

25. Sontag ED. A Dynamic Model of Immune Responses to Antigen Presentation Predicts Different Regions of Tumor or Pathogen Elimination. Cell Syst. 2017;4(2):231-41 e11. doi: 10.1016/j.cels.2016.12.003.

26. Redondo MJ, Jeffrey J, Fain PR, Eisenbarth GS, Orban T. Concordance for islet autoimmunity among monozygotic twins. N Engl J Med. 2008;359(26):2849-50. doi: 10.1056/NEJMc0805398.

27. Ziegler AG, Kick K, Bonifacio E, Haupt F, Hippich M, Dunstheimer D, et al. Yield of a Public Health Screening of Children for Islet Autoantibodies in Bavaria, Germany. JAMA. 2020;323(4):339-51. doi: 10.1001/jama.2019.21565.
