# Supplementary material for: Inhibitory KIRs decrease HLA class II-mediated protection in Type 1 Diabetes
Source: PLoS Genet. 2024 Dec 26;20(12):e1011456. doi: 10.1371/journal.pgen.1011456 (PMC11741628; doi:10.1371/journal.pgen.1011456)
Supplement: S1 Results — (DOCX) [file pgen.1011456.s002.docx]

## S1 Results

### iKIR modification of *DRB1*15:01-DQB1*06:02*

In the main text we focus on whether iKIR modify the *DQA1*01:02-DQB1*06:02* association as there is some evidence in the literature [1, 2] and in our cohort that the protection associated with the *DRB1*15:01-DQA1*01:02-DQB1*06:02* genotype maps to *DQA1*01:02-DQB1*06:02* rather than *DRB1*15:01-DQB1*06:02*. However, fine mapping is difficult, so in case the driver of protection is *DRB1*15:01-DQB1*06:02* we repeated our iKIR analysis focusing on *DRB1*15:01-DQB1*06:02* instead. As expected, (since 99.2% of *DQA1*01:02-DQB1*06:02* positive carriers are also *DRB1*15:01-DQB1*06:02*), the results are virtually identical (**Figs S6 and S7**, **Tables S8-S11**).

### Modification of *DQA1*01:02-DQB1*06:02* is attributable to functional iKIR rather just the HLA class I genes

An individual’s iKIR score will depend on their HLA class I genotype since the iKIR ligands are HLA class I molecules. Some HLA class I alleles are associated with protection from or susceptibility to T1D [3-5]. Therefore, our observation that *DQ6* protection is enhanced in individuals with a low iKIR score could be due to an interaction between HLA class I alleles and *DQ6* and have nothing to do with iKIR genes. To investigate whether our observations were attributable to the class I ligands effects rather than the iKIR genes, we repeated all the analysis with iKIR ligands (Bw4, C1, C2) included as covariates. The results were remarkably similar and iKIR score still significantly modified the *DQ6* protective effect even when iKIR ligands were included as covariates (**Table S12**)**.**

To further investigate whether the observed modification of *DQ6* was attributable to the effects of class I ligands rather than the iKIR genes, we constructed a score to reflect presence of the HLA ligand but absence of the matching iKIR. Although this score in itself was associated with significant protection (ln OR = -0.4 P =0.0009) in line with the published literature that HLA class I molecules are associated with risk of T1D, it had no impact on the protective effect of *DQ6* (P=0.99) when included as an interaction term ($OUTCOME \sim DQ6\times ligand\_no\_iKIR + GENDER$) suggesting that the effect we had observed for iKIR score (functional iKIRs) requires presence of the gene encoding the iKIR not just the HLA ligand. However, we were concerned that there was little variation in the count of ligands in absence of iKIR since most individuals have at least one functional iKIR in our cohort. This could have reduced the power of the test resulting in the non-significant result, so we also developed the “ligand count” measure which quantified the number of HLA ligands. This is a more problematic measure as it is significantly correlated with the iKIR score (corr=0.87, P<2x10^-16^) making it difficult to disentangle the effects. However, we found that iKIR score has a stronger effect than ligand count on *DQ6* (P=5.5x10^-6^ for iKIR score, P=7.6x10^-4^ for ligand count); in backwards stepwise regression (starting from a full model with both interaction terms) ligand count is removed from the model and the model with ligand count has a considerably lower AIC (difference=75). Upon standardising the variables for comparability, we also found iKIR score had a higher coefficient than the ligand count (0.62 for iKIR score 0.45 for ligand count). Additionally, in a model including both interaction terms, iKIR score remained significant (P= 9x10^-4^) whilst HLA ligand count became non-significant (P=0.29).

Finally, we assessed whether the iKIR score effect on *DQ6* was attributable to specific HLA class I drivers in our cohort associated with this haplotype. A detrimental Bw4 or a protective Bw6 allele in linkage disequilibrium with *DQ6* could potentially explain the iKIR score effect on *DQ6* since for example people with the detrimental Bw4 allele would tend to have a higher iKIR score than those without (being in possession of an iKIR ligand) and this detrimental effect could act to reduce the protective effect of *DQ6* in the iKIR low strata*.* Likewise, a protective Bw4 or a detrimental Bw6 allele negatively associated with *DQ6* could also be responsible for the *DQ6*:*iKIR_score* effect. If a particular HLA-B allele was responsible for the iKIR effect, then on removing individuals with this allele the iKIR score effect will disappear. We tested this hypothesis by discarding all individuals with a given allele and then performing regression analysis in the resulting subcohort. We repeated this process for all HLA class I alleles, one allele at a time. For all the HLAclassI_allele*^negative^* subcohorts, the *DQ6*:*iKIR_score* interaction term remained significant (**S13 Table**).

Overall, this set of results indicate that the observed functional iKIR modification of *DQ6* is not driven by HLA class I genes alone and suggests that the effect of functional iKIR on this protective genotype is due to the iKIR-ligand interaction.

### All functional iKIRs contribute to iKIR score modulation of *DQ6*

In a logistic regression model, the *DQ6*:Bw4 interaction term is significant (P=3.67x10^-4^), although iKIR score as an interaction term is more significant (P=2.1x10^-5^). Since 95% of Bw4 carriers carry the corresponding iKIR gene, *KIR3DL1*, we hypothesized that the *DQ6*:Bw4 interaction was reflecting the effect of *KIR3DL1-Bw4* gene pair on *DQ6* and that perhaps the iKIR score effect was only driven by functional *KIR3DL1*. Therefore, we investigated the contribution of the rest of iKIR-ligand pairs to the iKIR score by excluding non-functional *KIR3DL1* individuals from the cohort (so that the cohort was homogeneous for functional *KIR3DL1*) and then repeating the stratification analysis on the resulting subcohort. We obtained similar results despite the much smaller cohort size (**S8 Fig**). Similarly, *DQ6:iKIR_score* interaction term remains significant in this subcohort (ln[OR]=1.2, P=2.53x10^-4^) suggesting that KIR2DL1/KIR2DL2/KIR2DL3 also contribute to the modulatory effect along with KIR3DL1.

### Are activating KIRs modulating HLA associations?

Some studies have reported an association between activating KIRs (aKIRs) and increased risk of T1D [6, 7]. We therefore investigated whether the observed iKIR effect could be explained by activating KIR genes. We computed a measure similar to the iKIR count but for the functional activating KIRs, the aKIR Count, including all activating KIR with known ligands namely KIR2DS1, KIR2DS2, KIR2DS4 and KIR3DS1 [8]; lack of information about the strength of signaling of these ligand-aKIR interactions precluded development of a score for activating KIR. Although the iKIR score is significantly correlated with aKIR count (corr=0.65, P<2.2x10^-16^), in a model including both metrics interacting with *DQ6*, iKIR score remained significant whereas aKIR count did not.

### iKIR modulation is independent of detrimental class II haplotypes

The most significant detrimental genotypes in T1D are *DR3* (*DRB1*03:01-DQB1*02:01*) and *DR4* (*DRB1*04:01/02/04/05-DQB1*03:02*) [9]. We investigated whether the iKIR score effect on *DQ6* varied when these genotypes were included as covariates. We consistently found that, whilst the *DR3* and *DR4* genotypes were highly detrimental they had little or no impact on the interaction between iKIR score and *DQ6*; that is both the coefficient of the interaction (for standardised score) and the P-value were very similar with or without inclusion of the detrimental haplotypes (**S14 Table**). This indicates that the impact of iKIR on *DQ6* is independent of the strong detrimental genes of the *DR3* and *DR4* genotypes.

## Supplementary References

1. Hoover ML, Marta RT. Molecular modelling of HLA-DQ suggests a mechanism of resistance in type 1 diabetes. Scand J Immunol. 1997;45(2):193-202. doi: 10.1046/j.1365-3083.1997.d01-389.x.

2. Erlich HA, Griffith RL, Bugawan TL, Ziegler R, Alper C, Eisenbarth G. Implication of specific DQB1 alleles in genetic susceptibility and resistance by identification of IDDM siblings with novel HLA-DQB1 allele and unusual DR2 and DR1 haplotypes. Diabetes. 1991;40(4):478-81. doi: 10.2337/diab.40.4.478.

3. Nejentsev S, Howson JM, Walker NM, Szeszko J, Field SF, Stevens HE, et al. Localization of type 1 diabetes susceptibility to the MHC class I genes HLA-B and HLA-A. Nature. 2007;450(7171):887-92. doi: 10.1038/nature06406.

4. Noble JA, Valdes AM, Varney MD, Carlson JA, Moonsamy P, Fear AL, et al. HLA class I and genetic susceptibility to type 1 diabetes: results from the Type 1 Diabetes Genetics Consortium. Diabetes. 2010;59(11):2972-9. doi: 10.2337/db10-0699.

5. Howson JM, Stevens H, Smyth DJ, Walker NM, Chandler KA, Bingley PJ, et al. Evidence that HLA class I and II associations with type 1 diabetes, autoantibodies to GAD and autoantibodies to IA-2, are distinct. Diabetes. 2011;60(10):2635-44. doi: 10.2337/db11-0131.

6. Shastry A, Sedimbi SK, Rajalingam R, Nikitina-Zake L, Rumba I, Wigzell H, et al. Combination of KIR 2DL2 and HLA-C1 (Asn 80) confers susceptibility to type 1 diabetes in Latvians. Int J Immunogenet. 2008;35(6):439-46. doi: 10.1111/j.1744-313X.2008.00804.x.

7. Zhi D, Sun C, Sedimbi SK, Luo F, Shen S, Sanjeevi CB. Killer cell immunoglobulin-like receptor along with HLA-C ligand genes are associated with type 1 diabetes in Chinese Han population. Diabetes Metab Res Rev. 2011;27(8):872-7. doi: 10.1002/dmrr.1264.

8. Pende D, Falco M, Vitale M, Cantoni C, Vitale C, Munari E, et al. Killer Ig-Like Receptors (KIRs): Their Role in NK Cell Modulation and Developments Leading to Their Clinical Exploitation. Front Immunol. 2019;10:1179. doi: 10.3389/fimmu.2019.01179.

9. Erlich H, Valdes AM, Noble J, Carlson JA, Varney M, Concannon P, et al. HLA DR-DQ haplotypes and genotypes and type 1 diabetes risk: analysis of the type 1 diabetes genetics consortium families. Diabetes. 2008;57(4):1084-92. doi: 10.2337/db07-1331.
