# Supplementary material for: Inhibitory KIRs decrease HLA class II-mediated protection in Type 1 Diabetes
Source: PLoS Genet. 2024 Dec 26;20(12):e1011456. doi: 10.1371/journal.pgen.1011456 (PMC11741628; doi:10.1371/journal.pgen.1011456)
Supplement: S1 Text — (DOCX) [file pgen.1011456.s003.docx]

## S1 Text

**Definition of Functional iKIR and inhibitory score.**

An individual was defined to be positive for a functional KIR if they carried both the gene for the iKIR and the gene for its HLA ligand giving the following definitions:

| Functional *KIR2DL1* | *KIR2DL1* & *HLA*-*C2* |
| --- | --- |
| Strong Functional *KIR2DL2* | *KIR2DL2* & (*HLA*-*C1* or *B46* or *B73*) |
| Weak Functional *KIR2DL2* | *KIR2DL2* & *HLA-C2* |
| Functional *KIR2DL3* | *KIR2DL3* & (*HLA*-*C1* or *B46* or *B73*) |
| Functional *KIR3DL1* | *KIR3DL1* & *HLA-Bw4* |

Where *HLA-C1* denotes an HLA-C allele carrying a C1 motif (Asparagine at position 80); *HLA-C2* an HLA-C allele with a C2 motif (Lysine at position 80) and *HLA-Bw4* an HLA-B or HLA-A allele with a Bw4 motif (Asparagine at position 77). Despite carrying the Bw4 motif, HLA-A*25 does not bind to KIR3DL1 and so we do not consider *HLA-A*25* alleles as KIR ligands [1-3]. There is also evidence that, despite carrying a Bw4 motif, HLA-B*13 is not a KIR3DL1 ligand [1, 3]. Exclusion of *HLA-B*13* alleles from the KIR ligand definition had no qualitative impact on the results.

Two metrics were considered: count and score. The count is the number of functional iKIR that an individual possessed, and the inhibitory score is the count adjusted for the observation that KIR2DL2 binds C1 more strongly than it binds C2 and that KIR2DL2 binds C1 more strongly than KIR2DL3 binds C1. Functionally diverse alleles at the same locus (2DL2/L3 and 3DL1/S1) were scored differently to reflect different strengths of inhibitory signal.

Inhibitory score= (1 if Func 2DL1) + (1 if Strong Func 2DL2 or 0.5 if weak Func 2DL2) + (0.75 if Func 2DL3) + (1 if Func 3DL1).

When it was necessary to stratify the cohort into individuals with a high count and low count or into individuals with a high inhibitory score and a low inhibitory score, we analysed all thresholds that gave a sufficiently balanced stratification (similar number of people in each stratum).

## References

1. Foley BA, De Santis D, Van Beelen E, Lathbury LJ, Christiansen FT, Witt CS. The reactivity of Bw4(+) HLA-B and HLA-A alleles with KIR3DL1: implications for patient and donor suitability for haploidentical stem cell transplantations. Blood. 2008;112(2):435-43. doi: 10.1182/blood-2008-01-132902.

2. Stern M, Ruggeri L, Capanni M, Mancusi A, Velardi A. Human leukocyte antigens A23, A24, and A32 but not A25 are ligands for KIR3DL1. Blood. 2008;112(3):708-10. doi: 10.1182/blood-2008-02-137521.

3. Saunders PM, MacLachlan BJ, Widjaja J, Wong SC, Oates CVL, Rossjohn J, et al. The Role of the HLA Class I alpha 2 Helix in Determining Ligand Hierarchy for the Killer Cell Ig-like Receptor 3DL1 Philippa. Journal of Immunology. 2021;206(4):849-60. doi: 10.4049/jimmunol.2001109.
