# Supplementary figures and images for: Inhibitory KIRs decrease HLA class II-mediated protection in Type 1 Diabetes
Source: PLoS Genet. 2024 Dec 26;20(12):e1011456. doi: 10.1371/journal.pgen.1011456 (PMC11741628; doi:10.1371/journal.pgen.1011456)

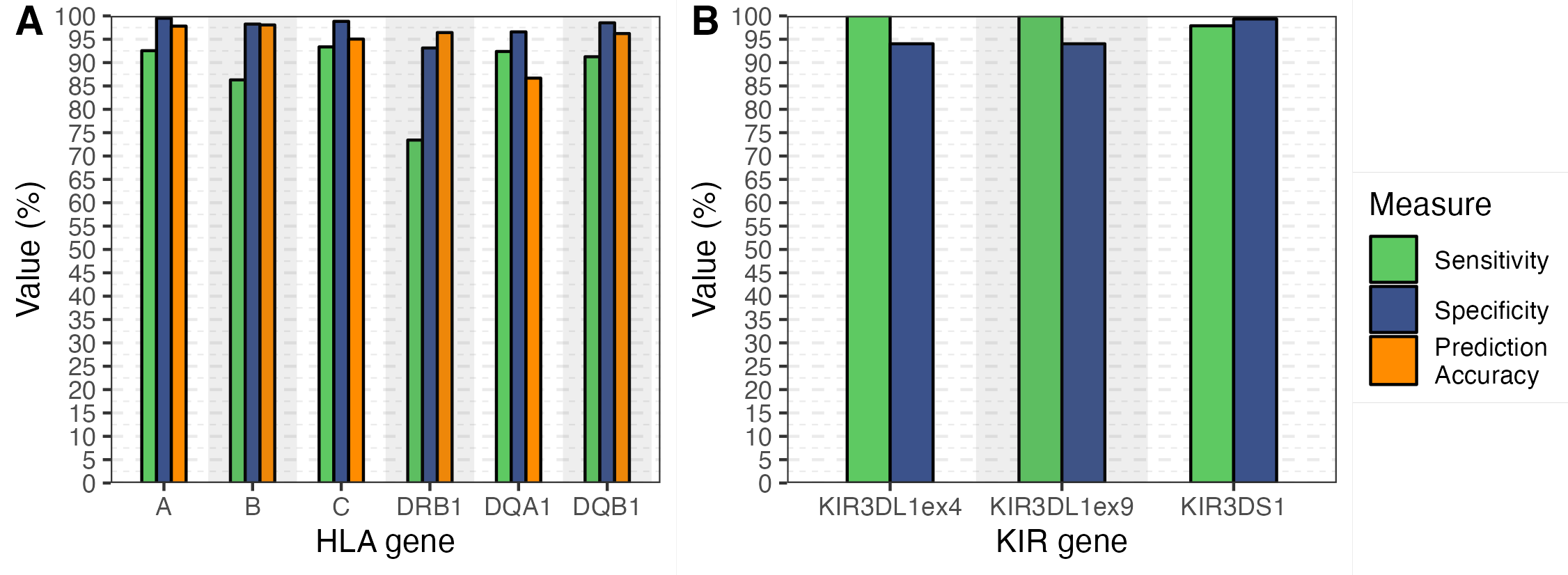

Supplement: S1 Fig — A Imputation accuracy measures at each HLA loci. Prediction accuracy is computed as the number of correctly imputed alleles divided by the number of experimentally typed chromosomes. B Sensitivity and specificity values at KIR3DL1/S1 locus. KIR3DL1 CN was measured using two assay methods, one targeting KIR3DL1 exon 4 (KIR3DL1ex4) and the other assay targeting exon 9 (KIR3DL1ex9). (TIFF) [file pgen.1011456.s006.tiff]

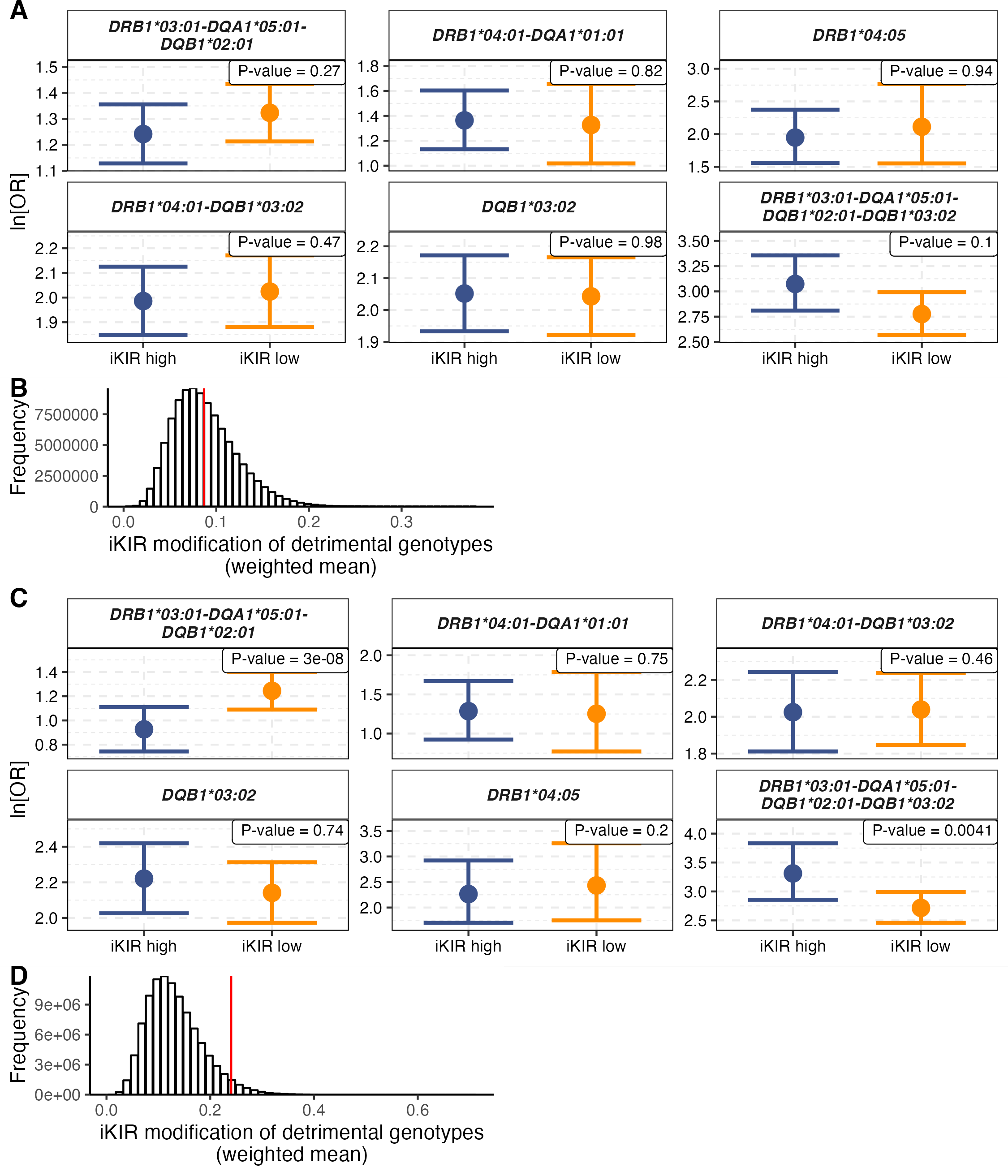

Supplement: S2 Fig — A In the whole cohort, for most detrimental genotypes, iKIR have no impact on the detrimental effect (the ln[OR] of the detrimental genotype is very similar in the strata with a high iKIR score (blue) and the strata with a low iKIR score (orange) and the 95% confidence intervals are overlapping). Two genotypes (DRB1*03:01-DQA1*05:01-DQB1*02:01 and DRB1*03:01-DQA1*05:01-DQB1*02:01-DQB1*03:02) show some evidence of iKIR modification but the modifications are in opposite directions. B The observed value of our test statistic (weighted mean of the difference in ln[OR] between the KIR high and the KIR low strata at threshold = 1.75), indicated by the red line, is entirely consistent with the distribution (grey histogram) of the same test statistic under the null hypothesis that the iKIR score has no impact on the detrimental genotypes (generated by permuting the iKIR score of individuals in the cohort). This indicates that the probability of obtaining our observation by chance is high (P = 0.46) and that there is no evidence to reject the null hypothesis of no iKIR modification. C Similarly in the cohort with HLA class I drivers removed. Most genotypes are not modified and where there is modification then results are in opposite directions. D In the cohort without HLA class I drivers the observed value of our test statistic (indicated by the red line), whilst still overlapping, is more of an outlier from the distribution (grey histogram) of the same test statistic under the null hypothesis that the iKIR score has no impact on the detrimental genotypes. This decrease in the test statistic is driven entirely by one genotype which is strongly iKIR modified (DRB1*03:01-DQA1*05:01-DQB1*02:01). Overall, there is some evidence for iKIR modification in this cohort (P = 0.04) but it is far from convincing. Examining the apparent iKIR modification of DRB1*03:01-DQA1*05:01-DQB1*02:01 we found that it was explained by the negative correlation of DRB1*03:01-DQA1*05:01-DQB1*02:01 with [file pgen.1011456.s007.tiff]

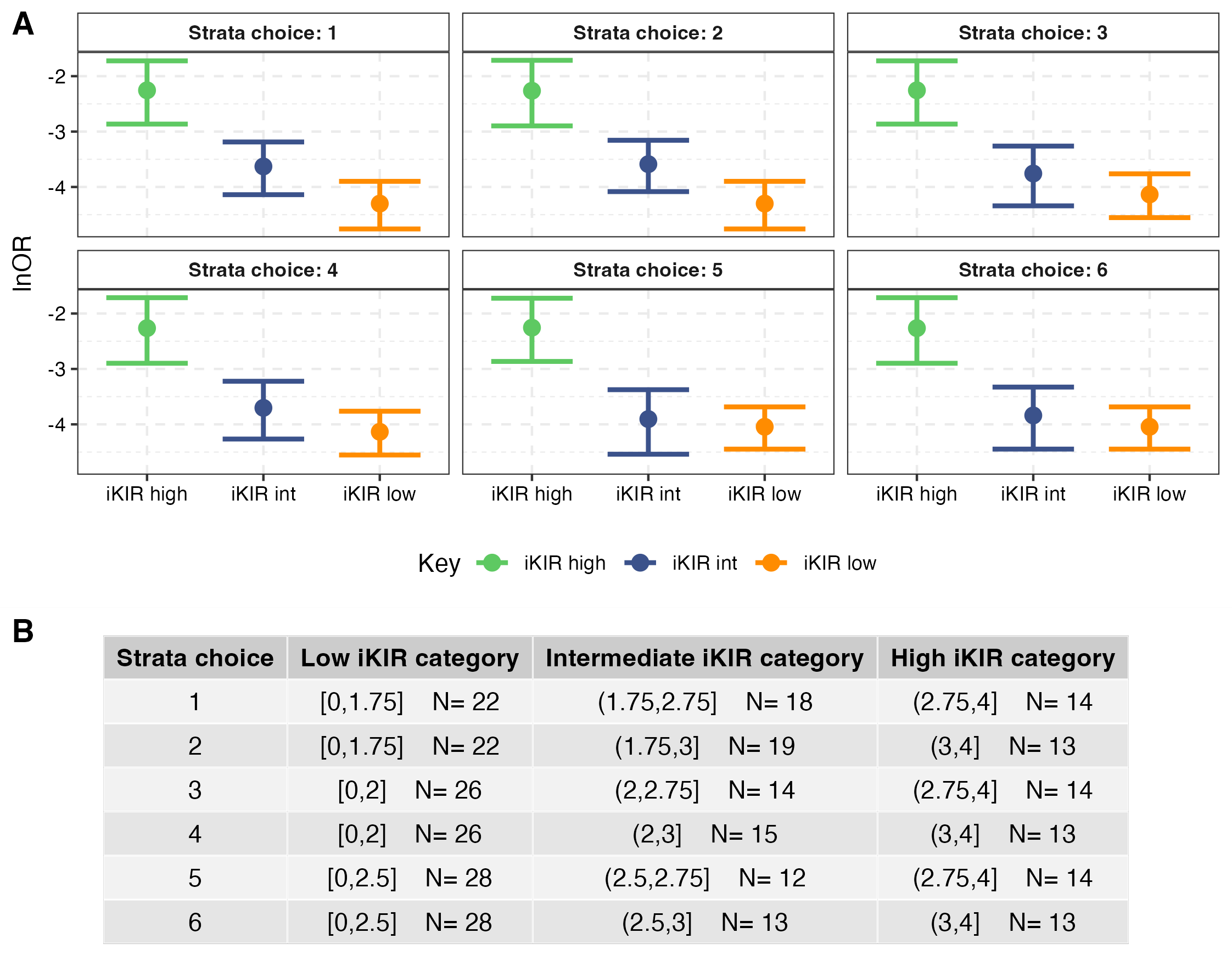

Supplement: S3 Fig — A DQ6 protection increases (i.e. ln[OR] becomes more negative) as the iKIR score decreases. B This was true for all strata choices (definitions of high, intermediate, low) considered as shown in the table. Subjects were categorized as having low, intermediate (int) and high iKIR score using all category cutoffs that ensured more than 12 individuals in each group. Coefficients, p-values and group sizes are reported in S2 Table. (TIFF) [file pgen.1011456.s008.tiff]

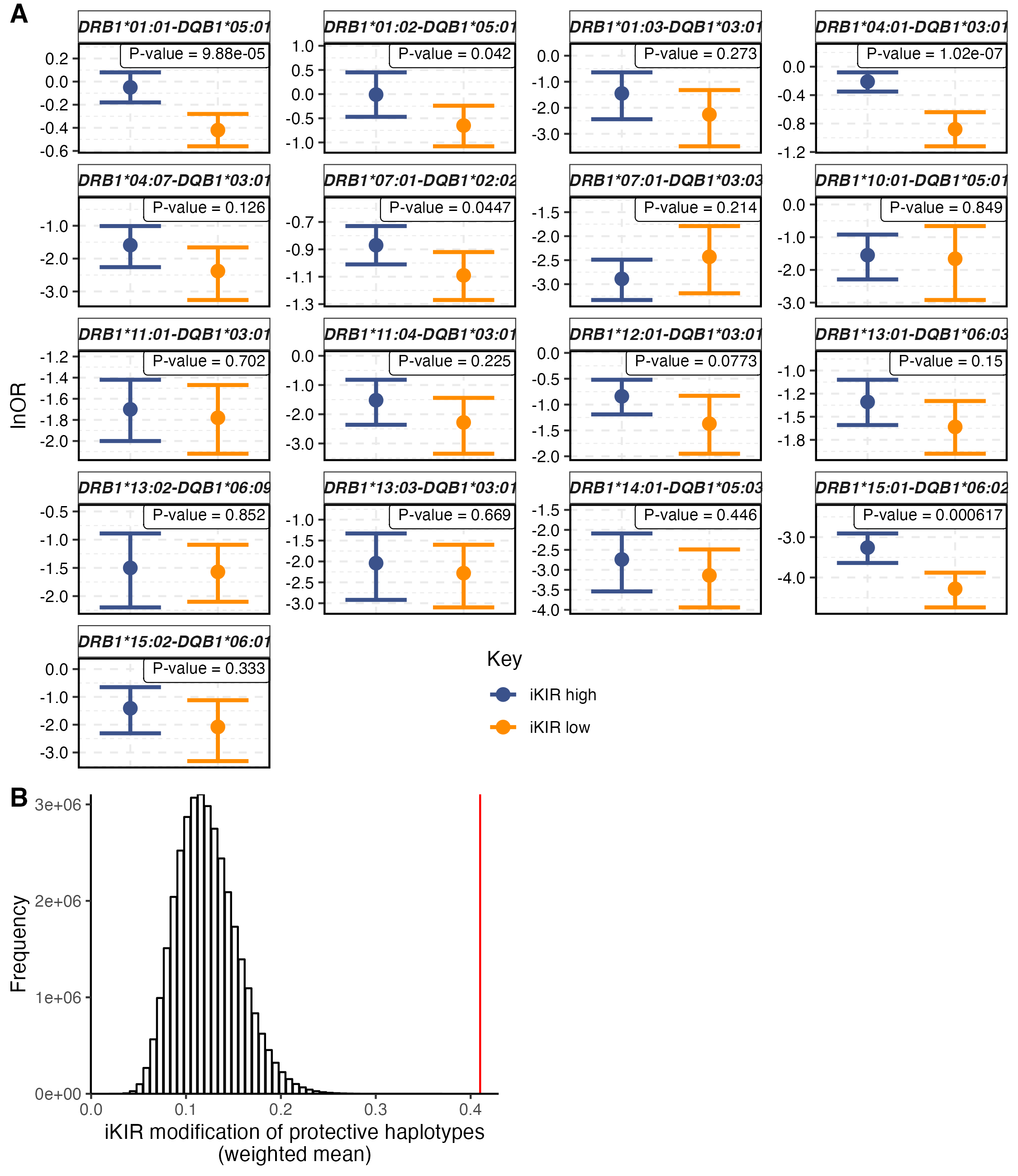

Supplement: S4 Fig — A The protective effect of class II DRB1-DQB1 haplotypes is enhanced in the group of individuals with an iKIR score equal to 1.75 or lower (iKIR score threshold = 1.75) with the exception of DRB1*07:01-DQB1*03:03. The number in the top right box corresponds to the odds of seeing this difference by chance (3×10^7^ permutations). The dot is the ln[OR] and the bars the 95% confidence intervals obtained from the regression, blue iKIR high strata, orange: iKIR low strata. B The observed value of our test statistic (weighted mean), indicated by the red arrow, lies far above the distribution (grey histogram) of the same test statistic under the null hypothesis that the iKIR score has no impact on the protective haplotypes (generated by permuting the iKIR score of individuals in the cohort, P<3×10^−7^). (TIFF) [file pgen.1011456.s009.tiff]

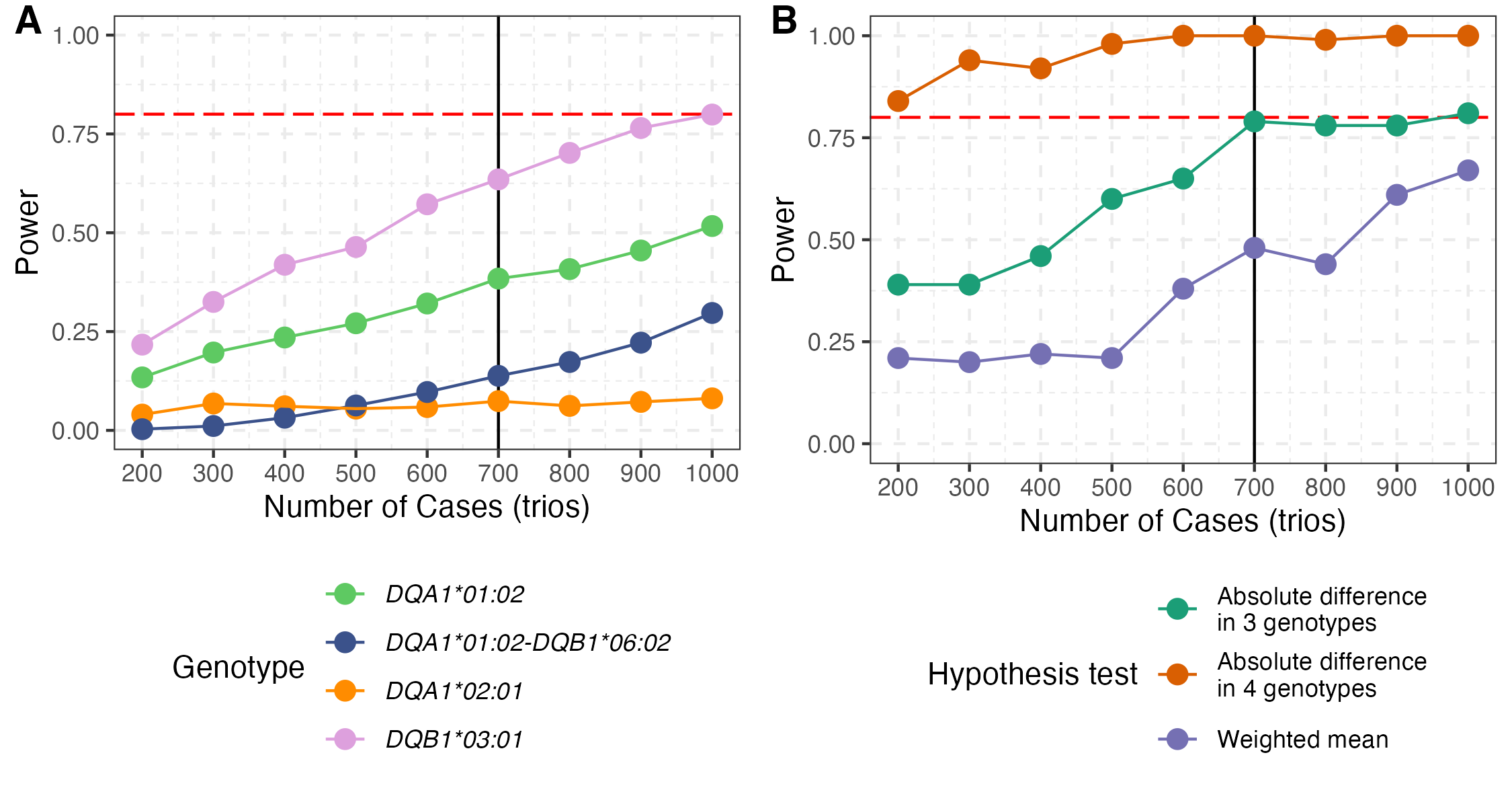

Supplement: S5 Fig — A For each sample size s (Number of cases = Number of controls = s/2), we generated 1000 random subcohorts by resampling with replacement individuals from the UK-GRID cohort. The sample size of the family dataset is 700 trios (indicated by a black line). For each protective genotype and each subcohort we run a logistic regression model with stratified iKIR score (threshold = 1.75) as an interaction term with the protective genotype. Power is estimated as the number of subcohorts where the coefficient of interaction was significant (P<0.05). The dashed red line indicates the power standard of 0.8. Given the sample size of the family dataset, the power to detect a significant iKIR score interaction on individual protective genotypes falls considerably below the recommendation of 0.8. B For each sample size, we estimated the power to detect significant difference between iKIR high and iKIR low strata (assessed by permutation test) across several protective genotypes simultaneously. Power is calculated as the proportion of cohorts with a significant permutation test. Given the sample size of the family dataset, the power of detecting an iKIR difference across 4 frequent protective genotypes i.e., DQA1*01:02, DQB1*03:01, DQA1*02:01 and DQA1*01:02-DQB1*06:02 is higher than the recommended standard of 0.8. Sample size family dataset (vertical black line), recommended power (horizontal dashed red line). (TIFF) [file pgen.1011456.s010.tiff]

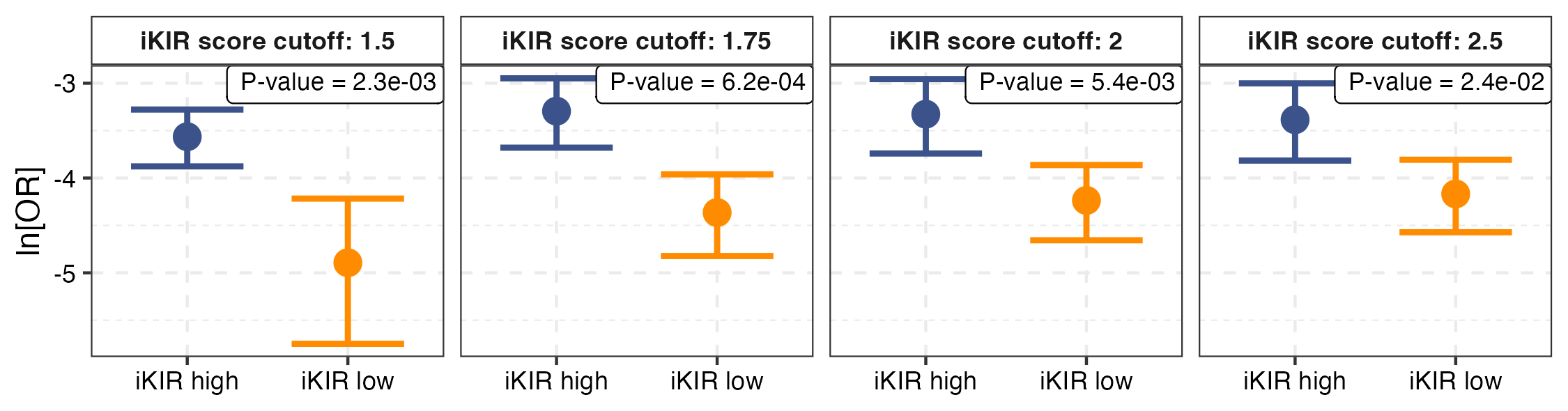

Supplement: S6 Fig — Stratification analysis was repeated for different iKIR thresholds but this time including ligands as covariates in the model (OUTCOME∼DRB1*15:01−DQB1*06:02+GENDER+Bw4+C1+C2). The results are remarkably similar to our previous analysis on DQ6 (see main text Fig 1). Estimates, p-values and cohort sizes are reported in S8 Table. (TIFF) [file pgen.1011456.s011.tiff]

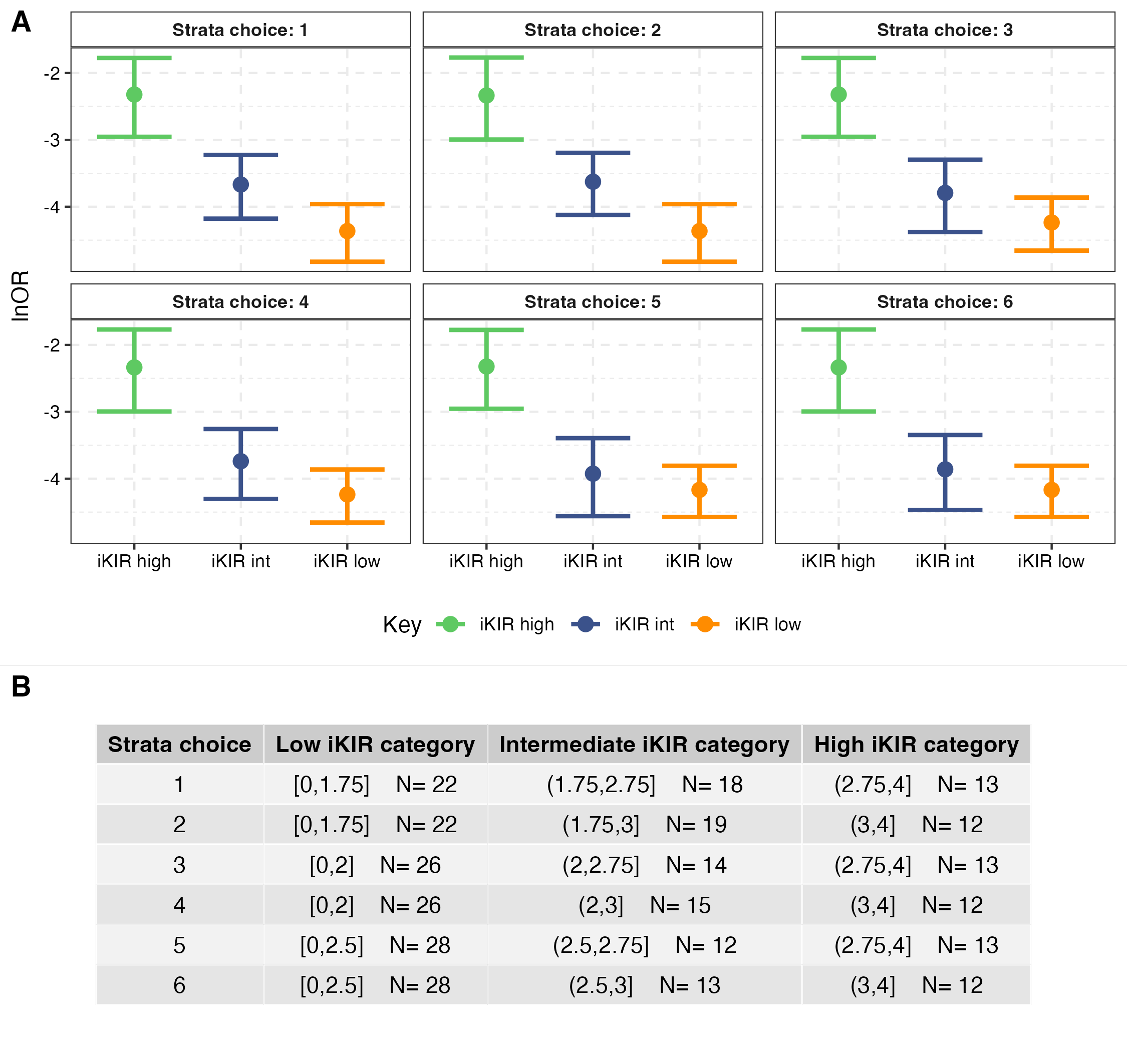

Supplement: S7 Fig — A The ln[OR] of DRB1*15:01-DQB1*06:02 decreases (i.e. becomes more protective) as the iKIR score decreases. B This was true for all strata choices (i.e. definitions of high, intermediate, low) considered as shown in the table (see also S9 Table). Subjects were categorized as having low, intermediate (int) and high iKIR score using different thresholds that ensured enough number of individuals in each group (at least N = 12). Here, ligands (Bw4, C1 and C2) were included in the model as covariates. These results for DRB1*15:01-DQB1*06:02 are very similar to the results for DQA1*01:02-DQB1*06:02 (see S3 Fig). (TIFF) [file pgen.1011456.s012.tiff]

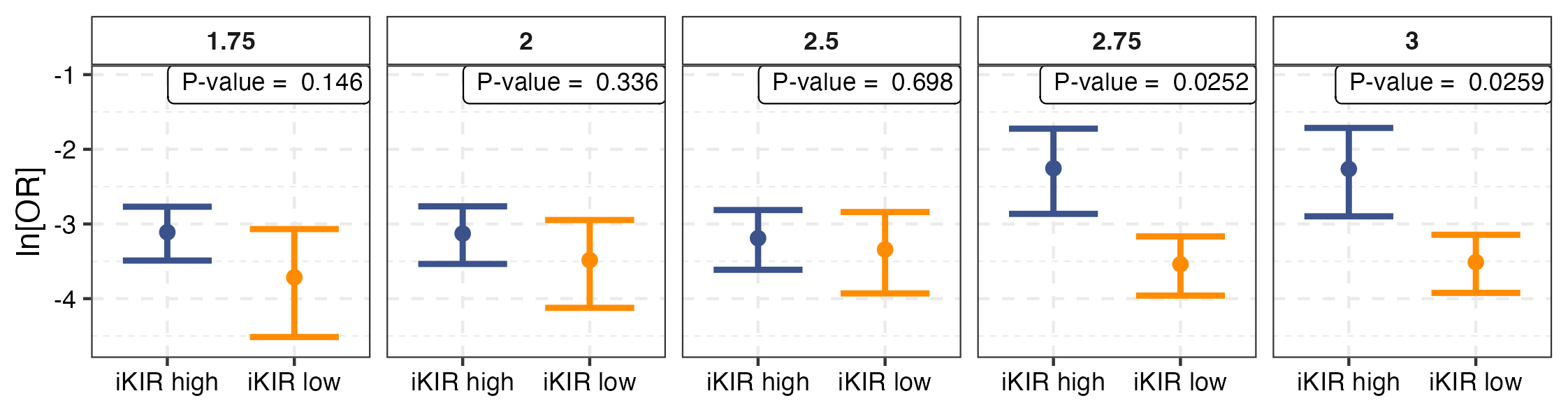

Supplement: S8 Fig — In a subcohort in which all individuals carry functional KIR3DL1 gene we still observe an enhanced protection of DQ6 in individuals with low iKIR score. The number on the top right box corresponds to the odds of seeing this difference by chance (108 permutations). (TIFF) [file pgen.1011456.s013.tiff]

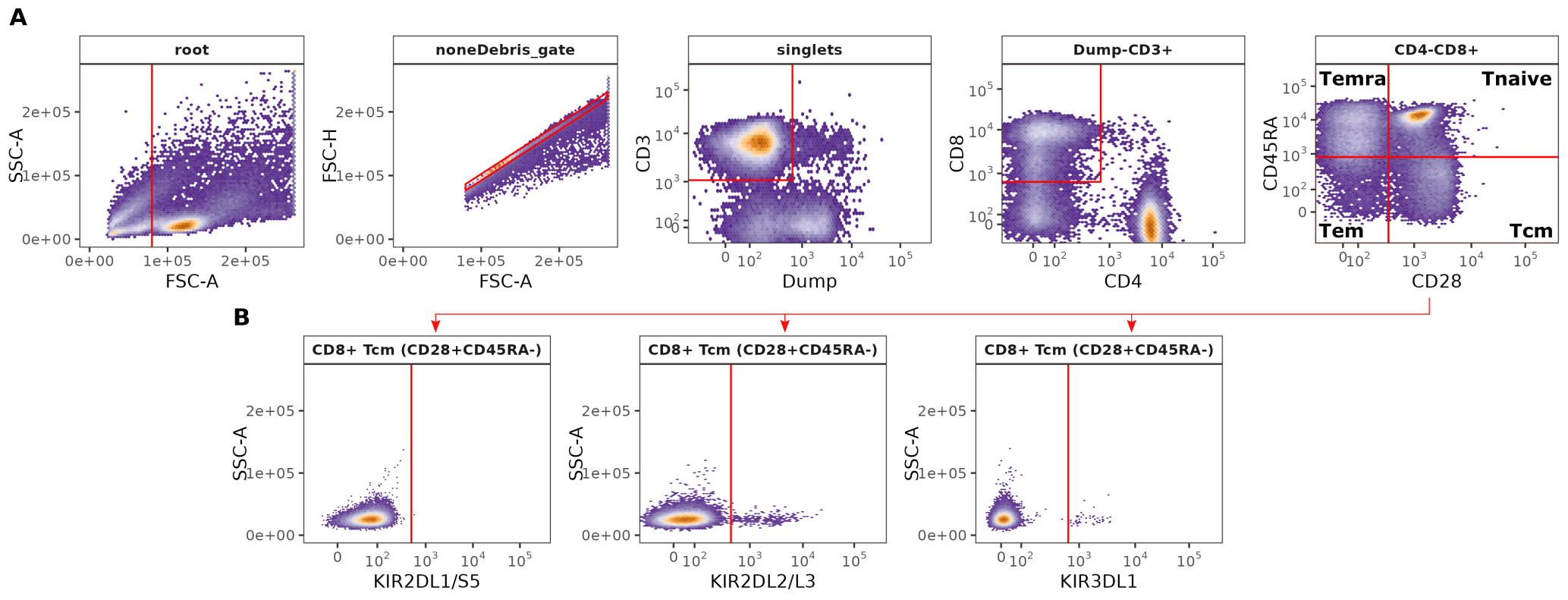

Supplement: S9 Fig — Analysis of PBMCs for a healthy donor (LD1). Each subplot shows events in the parent population, where strip names indicate parent population (root = all events). A From left to right, serial gating is used to identify (1) lymphocytes and discard debris (root, all events), (2) single cells in the nonDebris gate, (3) CD3+ and Dump−cells in the singlet gate (excluding unwanted lineages like CD14, CD19 and also necrotic cells), (4) CD8+ T cells in the Dump–CD3+ gate and (5) naive (Tnaive), central memory (Tcm), effector memory (Tem) and effector memory RA+ (Temra) populations within the CD8+ gate using CD45RA and CD28 staining. B Each of the 4 subsets defined in the CD8+ gate (naive and memory subsets) was gated to determine events positive for KIR2DL1 (left), KIR2DL2/L3 (middle) and KIR3DL1 (left). In this case, only KIR gates within the Tcm population are shown but the same strategy is followed for Tem, Temra and Tnaive subsets. All boundaries are determined with the 1D mindensity function (except for singlets) using collapsed data across all individuals. (PNG) [file pgen.1011456.s014.png]

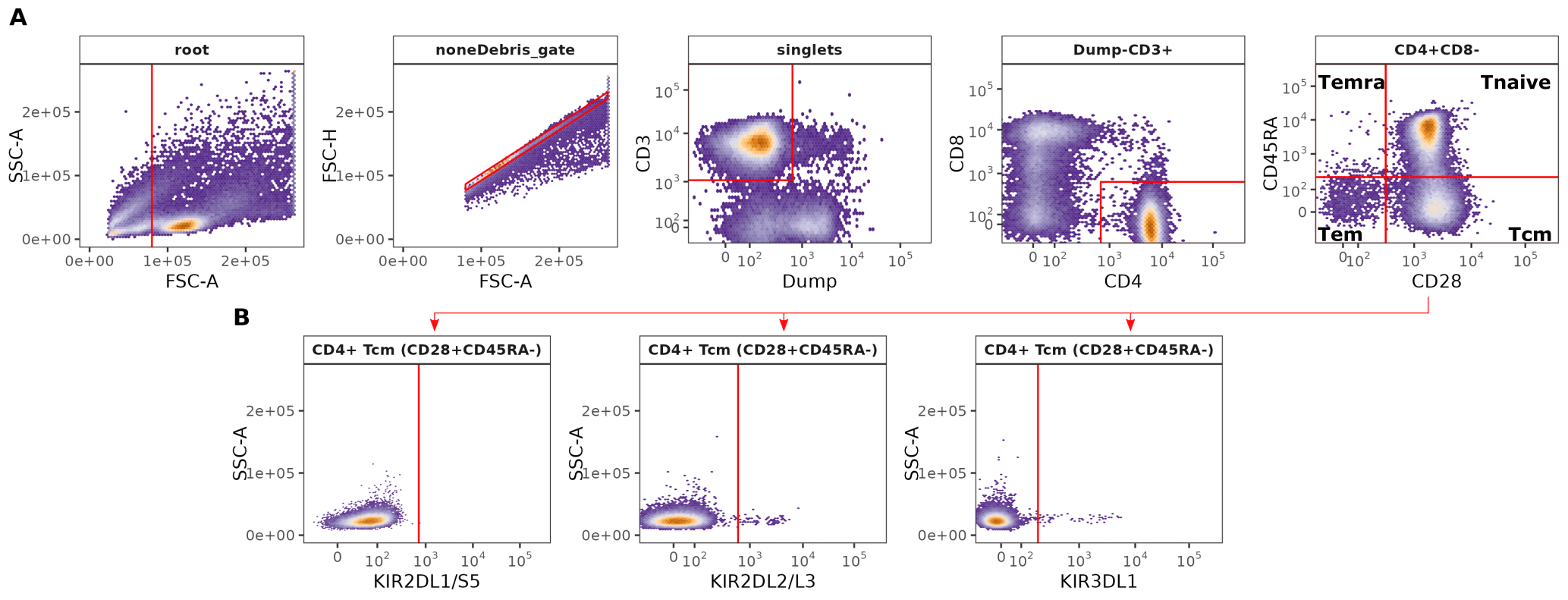

Supplement: S10 Fig — Analysis of PBMCs for a healthy donor (LD1). Each subplot shows events in the parent population, where strip names indicate parent population (root = all events). A From left to right, serial gating is used to identify (1) lymphocytes and discard debris (root, all events), (2) single cells in the nonDebris gate, (3) CD3+ and Dump−cells in the singlet gate (excluding unwanted lineages like CD14, CD19 and also necrotic cells), (4) CD4+ T cells in the Dump–CD3+ gate and (5) naive (Tnaive), central memory (Tcm), effector memory (Tem) and effector memory RA+ (Temra) populations within the CD4+ gate using CD45RA and CD28 staining. B Each of the 4 subsets defined in the CD4+ gate (naive and memory subsets) was gated to determine events positive for KIR2DL1 (left), KIR2DL2/L3 (middle) and KIR3DL1 (left). In this case, only KIR gates within the Tcm population are shown but the same strategy is followed for Tem, Temra and Tnaive subsets. All boundaries are determined with the 1D mindensity function (except for singlets) using collapsed data across all individuals. (PNG) [file pgen.1011456.s015.png]

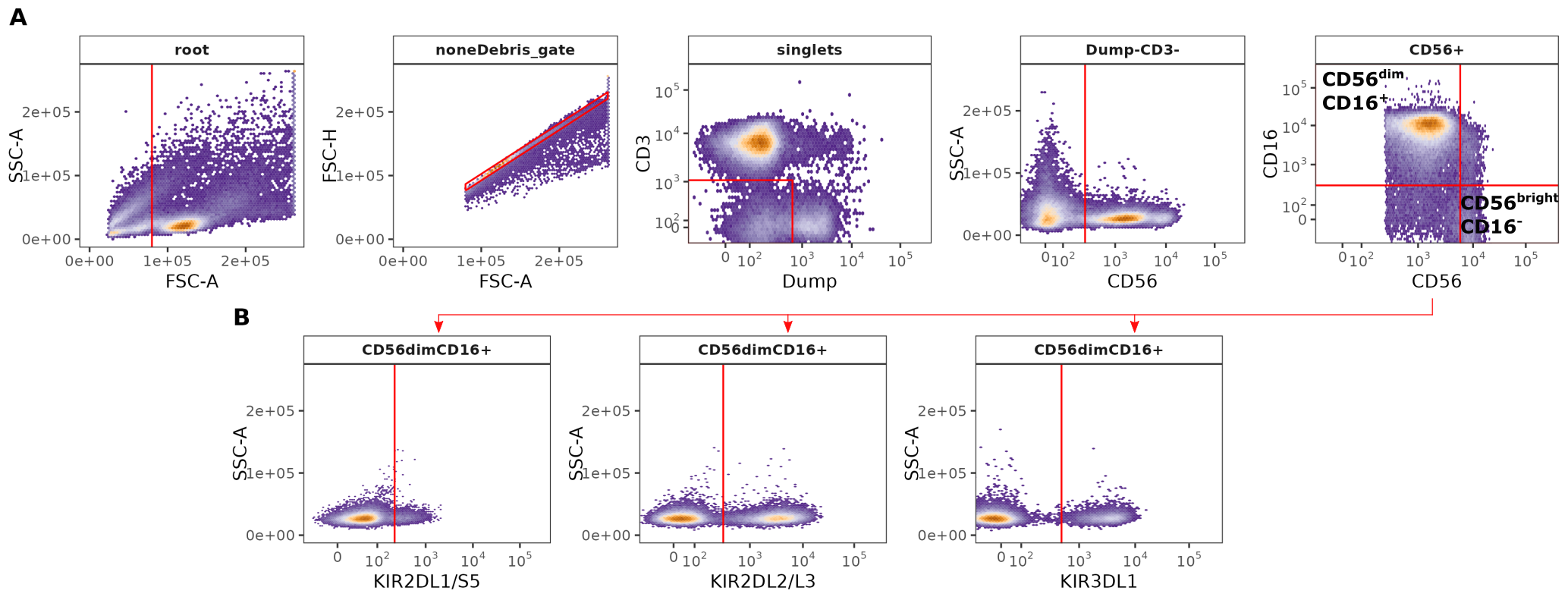

Supplement: S11 Fig — Analysis of PBMCs for a healthy donor (LD1). Each subplot shows events in the parent population, where strip names indicate parent population (root = all events). A From left to right, serial gating is used to identify (1) lymphocytes, (2) single cells, (3) CD3- cells (CD3-Dump–), (4) NK cells using CD56 staining and (5) CD56dimCD16+ and CD56brightCD16– populations using CD56 and CD16 staining. B CD56dimCD16+ and CD56brightCD16– populations were gated to determine events positive for KIR2DL1 (left), KIR2DL2/L3 (middle) and KIR3DL1 (left). In this case, only KIR gates within the CD56dimCD16+ population are shown. All boundaries are determined with the 1D mindensity function (except for singlets) using collapsed data across all individuals. (PNG) [file pgen.1011456.s016.png]

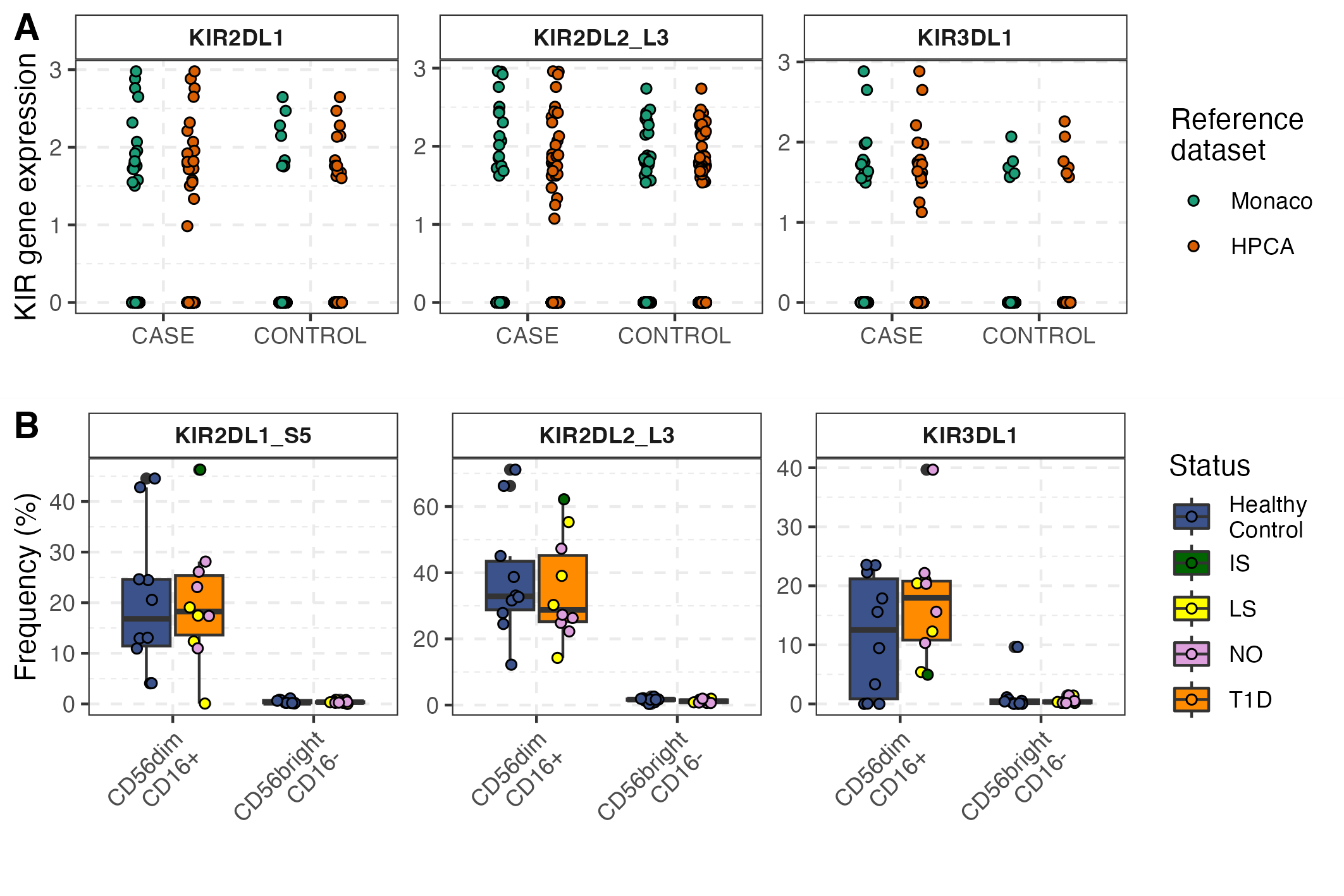

Supplement: S12 Fig — A KIR gene expression in NK cells split by disease status (CASE = seropositive individuals, CONTROL = matched healthy individuals) and by reference dataset used for cell annotation (green = Monaco reference, orange = Human Primary Cell Atlas reference). Each dot indicates a single cell barcode. Cells labelled as Natural killer cells (Monaco reference) or NK_cells, NK_cell:CD56hiCD62L+, NK_cell:IL2 (Human Primary Cell Atlas reference) are shown. B Percentage of NK cells expressing different iKIR in T1D patients and healthy controls. The percentage of KIR+ cells in each NK cell population (CD56dimCD16+ and CD56brightCD16–) was quantified by flow cytometry. Dots represent cell frequencies from parent population for each individual. T1D samples are colour coded according to disease duration at time of collection (NO = new onset, IS = intermediate standing disease, LS = long standing disease). Boxes show medians and interquartile ranges within T1D individuals (N = 10, orange, irrespective of disease duration) and healthy individuals (N = 10, blue). (TIFF) [file pgen.1011456.s017.tiff]
