# Supplementary material for: Inhibitory KIRs decrease HLA class II-mediated protection in Type 1 Diabetes
Source: PLoS Genet. 2024 Dec 26;20(12):e1011456. doi: 10.1371/journal.pgen.1011456 (PMC11741628; doi:10.1371/journal.pgen.1011456)
Supplement: S1 Table — The cohort was stratified into individuals with high or low iKIR score using different iKIR score thresholds (1.5, 1.75, 2.0 and 2.5). The protective effect of DQ6 was evaluated independently in each stratum using multivariate logistic regression with gender included as a covariate. DQ6 is significantly protective (ln[OR] = -3.74, P = 1.05×10−157) in the UK-GRID cohort (Group = Whole cohort, unstratified analysis). Regression coefficients, p-values and cohort sizes are reported for the different strata. P-value for the unstratified analysis calculated using Wald-test, all other p-values calculated using a permutation test. (PDF) [file pgen.1011456.s018.pdf]

	Group	ln[OR]	2.50%	97.50%	P-value	N genotype +		N genotype-	
						Cases	Controls	Cases	Controls
	Whole cohort	-3.741	-4.026	-3.477	1.05×10^{-157}	54	1545	6165	4197
Threshold=1.5	iKIR high	-3.49	-3.79	-3.20	1.82×10^{-3}	47	1165	4692	3573
	iKIR low	-4.85	-5.71	-4.18		7	380	1473	624
Threshold=1.75	iKIR high	-3.23	-3.61	-2.89	5.6×10^{-4}	32	721	2771	2463
	iKIR low	-4.30	-4.76	-3.90		22	824	3394	1734
Threshold=2.0	iKIR high	-3.26	-3.67	-2.90	4.62×10^{-3}	28	629	2400	2067
	iKIR low	-4.13	-4.55	-3.76		26	916	3765	2130
Threshold=2.5	iKIR high	-3.33	-3.75	-2.95	1.87×10^{-2}	26	611	2267	1911
	iKIR low	-4.04	-4.45	-3.68		28	934	3898	2286

S1 Table. *DQ6* protection in T1D is enhanced amongst individuals with a low iKIR score. The cohort was stratified into individuals with high or low iKIR score using different iKIR score thresholds (1.5, 1.75, 2.0 and 2.5). The protective effect of DQ6 was evaluated independently in each stratum using multivariate logistic regression with gender included as a covariate. DQ6 is significantly protective (ln[OR]= -3.74, P=1.05x10-157) in the GRID cohort (Group=Whole cohort, unstratified analysis). Regression coefficients, p-values and cohort sizes are reported for the different strata. P-value for the unstratified analysis calculated using Wald-test, all other p-values calculated using a permutation test.
