# Supplementary material for: Inhibitory KIRs decrease HLA class II-mediated protection in Type 1 Diabetes
Source: PLoS Genet. 2024 Dec 26;20(12):e1011456. doi: 10.1371/journal.pgen.1011456 (PMC11741628; doi:10.1371/journal.pgen.1011456)
Supplement: S2 Table — Individuals were stratified into High, Intermediate and Low iKIR score categories using 6 different definitions of high, intermediate and low (i.e. 6 different strata choices). For all strata choices we observe the same picture: DQ6 protection increases as iKIR score decreases. (PDF) [file pgen.1011456.s019.pdf]

Strata choice	Group		ln[OR]	2.50%	97.50%	N genotype+		N genotype-	
						Cases	Controls	Cases	Controls
1	High	(2.75,4]	-2.26	-2.86	-1.72	14	126	513	480
	Int	(1.75,2.75]	-3.63	-4.14	-3.19	18	595	2258	1983
	Low	[0,1.75]	-4.30	-4.76	-3.90	22	824	3394	1734
2	High	(3,4]	-2.26	-2.90	-1.71	13	120	469	446
	Int	(1.75,3]	-3.59	-4.08	-3.16	19	601	2302	2017
	Low	[0,1.75]	-4.30	-4.76	-3.90	22	824	3394	1734
3	High	(2.75,4]	-2.26	-2.86	-1.72	14	126	513	480
	Int	(2,2.75]	-3.76	-4.34	-3.26	14	503	1887	1587
	Low	[0,2]	-4.13	-4.55	-3.76	26	916	3765	2130
4	High	(3,4]	-2.26	-2.90	-1.71	13	120	469	446
	Int	(2,3]	-3.70	-4.27	-3.22	15	509	1931	1621
	Low	[0,2]	-4.13	-4.55	-3.76	26	916	3765	2130
5	High	(2.75,4]	-2.26	-2.86	-1.72	14	126	513	480
	Int	(2.5,2.75]	-3.90	-4.54	-3.37	12	485	1754	1431
	Low	[0,2.5]	-4.04	-4.45	-3.68	28	934	3898	2286
6	High	(3,4]	-2.26	-2.90	-1.71	13	120	469	446
	Int	(2.5,3]	-3.84	-4.45	-3.33	13	491	1798	1465
	Low	[0,2.5]	-4.04	-4.45	-3.68	28	934	3898	2286

S2 Table. iKIR score impacts *DQ6* associated protection in a dose-dependent manner. Individuals were stratified into High, Intermediate and Low iKIR score categories using 6 different definitions of high, intermediate and low (i.e. 6 different strata choices). For all strata choices we observe the same picture: *DQ6* protection increases as iKIR score decreases.
