# Supplementary material for: Inhibitory KIRs decrease HLA class II-mediated protection in Type 1 Diabetes
Source: PLoS Genet. 2024 Dec 26;20(12):e1011456. doi: 10.1371/journal.pgen.1011456 (PMC11741628; doi:10.1371/journal.pgen.1011456)
Supplement: S3 Table — iKIR score effect on HLA class II mediated protection was assessed for 17 significantly protective phased haplotypes in our cohort by stratifying the cohort into individuals with a high iKIR score and individuals with a low iKIR score and the protective effect of the haplotype calculated separately in the two strata. Regression coefficients, 95% confidence intervals, p-values and counts in each stratum are reported for each haplotype. The protective effect of class II haplotypes is enhanced in the group of individuals with a low iKIR score (an iKIR score equal to 1.75 or lower) with the exception of DRB1*07:01-DQB1*03:03. The odds of seeing this difference by chance were assessed by permutation test for each haplotype (3×10^7^ permutations). Wald test p-values are reported for the unstratified analysis (Group = Whole cohort). (PDF) [file pgen.1011456.s020.pdf]

Haplotype	Group	lnOR	2.50%	97.50%	P-value	N haplotype +		N haplotype -	
						Cases	Controls	Cases	Controls
DRB1*01:01-DQB1*05:01	Whole cohort	-0.24	-0.34	-0.15	3.07E-07	1021	1150	5198	4592
	iKIR high	-0.05	-0.18	0.08	9.88E-05	545	644	2258	2540
	iKIR low	-0.42	-0.56	-0.28		476	506	2940	2052
DRB1*01:02-DQB1*05:01	Whole cohort	-0.34	-0.65	-0.03	3.18E-02	73	94	6146	5648
	iKIR high	-0.01	-0.47	0.45	4.20E-02	35	40	2768	3144
	iKIR low	-0.65	-1.08	-0.24		38	54	3378	2504
DRB1*01:03-DQB1*03:01	Whole cohort	-1.83	-2.56	-1.20	9.77E-08	10	57	6209	5685
	iKIR high	-1.45	-2.44	-0.64	2.73E-01	6	29	2797	3155
	iKIR low	-2.26	-3.48	-1.32		4	28	3412	2530
DRB1*04:01-DQB1*03:01	Whole cohort	-0.49	-0.61	-0.38	1.42E-16	538	772	5681	4970
	iKIR high	-0.21	-0.35	-0.08	1.02E-07	429	583	2374	2601
	iKIR low	-0.88	-1.12	-0.64		109	189	3307	2369
DRB1*04:07-DQB1*03:01	Whole cohort	-1.95	-2.47	-1.49	3.22E-15	19	121	6200	5621
	iKIR high	-1.59	-2.26	-1.01	1.26E-01	12	66	2791	3118
	iKIR low	-2.38	-3.26	-1.66		7	55	3409	2503
DRB1*07:01-DQB1*02:02	Whole cohort	-1.01	-1.12	-0.90	2.48E-73	542	1189	5677	4553
	iKIR high	-0.87	-1.01	-0.73	4.47E-02	321	751	2482	2433
	iKIR low	-1.09	-1.27	-0.92		221	438	3195	2120
DRB1*07:01-DQB1*03:03	Whole cohort	-2.88	-3.26	-2.55	2.54E-57	33	501	6186	5241
	iKIR high	-2.89	-3.33	-2.49	2.14E-01	24	427	2779	2757
	iKIR low	-2.43	-3.19	-1.79		9	74	3407	2484
DRB1*10:01-DQB1*05:01	Whole cohort	-1.67	-2.29	-1.13	1.20E-08	14	69	6205	5673
	iKIR high	-1.55	-2.29	-0.92	8.49E-01	10	53	2793	3131
	iKIR low	-1.66	-2.92	-0.66		4	16	3412	2542
DRB1*11:01-DQB1*03:01	Whole cohort	-1.77	-1.99	-1.55	2.38E-57	101	508	6118	5234
	iKIR high	-1.70	-2.00	-1.42	7.02E-01	55	315	2748	2869
	iKIR low	-1.78	-2.12	-1.47		46	193	3370	2365
DRB1*11:04-DQB1*03:01	Whole cohort	-1.88	-2.51	-1.32	4.20E-10	13	77	6206	5665
	iKIR high	-1.52	-2.36	-0.82	2.25E-01	8	41	2795	3143
	iKIR low	-2.28	-3.35	-1.44		5	36	3411	2522
DRB1*12:01-DQB1*03:01	Whole cohort	-1.08	-1.38	-0.80	9.92E-14	66	176	6153	5566
	iKIR high	-0.84	-1.19	-0.52	7.73E-02	49	126	2754	3058
	iKIR low	-1.37	-1.95	-0.83		17	50	3399	2508
DRB1*13:01-DQB1*06:03	Whole cohort	-1.49	-1.68	-1.31	2.34E-56	149	563	6070	5179

	iKIR high	-1.34	-1.59	-1.10	1.50E-01	86	342	2717	2842
	iKIR low	-1.61	-1.90	-1.33		63	221	3353	2337
DRB1*13:02-DQB1*06:09	Whole cohort	-1.50	-1.91	-1.12	1.04E-13	31	126	6188	5616
	iKIR high	-1.50	-2.20	-0.89	8.52E-01	11	55	2792	3129
	iKIR low	-1.57	-2.10	-1.09		20	71	3396	2487
DRB1*13:03-DQB1*03:01	Whole cohort	-2.15	-2.73	-1.65	5.05E-15	15	118	6204	5624
	iKIR high	-2.04	-2.92	-1.33	6.69E-01	7	61	2796	3123
	iKIR low	-2.28	-3.10	-1.60		8	57	3408	2501
DRB1*14:01-DQB1*05:03	Whole cohort	-2.94	-3.49	-2.47	5.08E-30	16	265	6203	5477
	iKIR high	-2.74	-3.54	-2.09	4.46E-01	8	134	2795	3050
	iKIR low	-3.14	-3.94	-2.49		8	131	3408	2427
DRB1*15:01-DQB1*06:02	Whole cohort	-3.75	-4.04	-3.48	2.11E-155	53	1533	6166	4209
	iKIR high	-3.26	-3.64	-2.91	6.17E-04	31	717	2772	2467
	iKIR low	-4.28	-4.74	-3.88		22	816	3394	1742
DRB1*15:02-DQB1*06:01	Whole cohort	-1.71	-2.42	-1.11	2.24E-07	11	55	6208	5687
	iKIR high	-1.41	-2.31	-0.65	3.33E-01	7	32	2796	3152
	iKIR low	-2.08	-3.31	-1.12		4	23	3412	2535

S3 Table. Impact of functional iKIR on 17 significantly protective *DRB1-DQB1* haplotypes. iKIR score effect on HLA class II mediated protection was assessed for 17 significantly protective phased haplotypes in our cohort by stratifying the cohort into individuals with a high iKIR score and individuals with a low iKIR score and the protective effect of the haplotype calculated separately in the two strata. Regression coefficients, 95% confidence intervals, p-values and counts in each stratum are reported for each haplotype. The protective effect of class II haplotypes is enhanced in the group of individuals with a low iKIR score (an iKIR score equal to 1.75 or lower) with the exception of *DRB1*07:01-DQB1*03:03*. The odds of seeing this difference by chance were assessed by permutation test for each haplotype ($3x10^7$ permutations). Wald test p-values are reported for the unstratified analysis (Group=Whole cohort).
