# Supplementary material for: Inhibitory KIRs decrease HLA class II-mediated protection in Type 1 Diabetes
Source: PLoS Genet. 2024 Dec 26;20(12):e1011456. doi: 10.1371/journal.pgen.1011456 (PMC11741628; doi:10.1371/journal.pgen.1011456)
Supplement: S4 Table — This table shows the “driver” genotypes which were independently associated with outcome (see Fig 3). The natural log of the odds ratio (ln[OR]), associated p-value for each genotype and number of cases and controls carrying the genotype is shown. ln[OR] and p-values were obtained by multiple logistic regression in the whole cohort with gender as an additional covariate is reported (i.e. the coefficients and p-values are derived in a model containing one genotype at a time). (PDF) [file pgen.1011456.s021.pdf]

	genotype	ln OR	p value	N cases	N controls
protective	*DQA1*01:02-DQA1*01:03*	-4.24	2.63E-09	2	125
	*DQA1*01:02-DQB1*06:03*	-3.85	4.30E-11	3	128
	*DQA1*01:02-DQB1*06:02*	-3.74	1.10E-157	54	1545
	*DQA1*01:01-DQA1*05:05-DQB1*03:01-DQB1*05:01*	-3.08	2.00E-13	6	118
	*DRB1*14:01-DQA1*01:04-DQB1*05:03*	-2.94	5.10E-30	16	265
	*DQA1*02:01-DQB1*03:03*	-2.60	3.10E-63	46	524
	*DQA1*01:02-DQB1*05:01*	-2.08	5.20E-37	43	304
	*DRB1*07:01-DQA1*02:01-DQB1*05:01*	-2.06	3.90E-28	33	232
	*DRB1*04:07-DQA1*03:03-DQB1*03:01*	-1.95	3.20E-15	19	121
	*DQA1*01:02*	-1.80	5.60E-249	526	2051
	*DQA1*05:05-DQB1*03:01*	-1.70	9.20E-104	213	933
	*DRB1*10:01-DQA1*01:05-DQB1*05:01*	-1.67	1.20E-08	14	69
	*B*57:01*	-1.67	5.90E-52	103	474
	*DQA1*01:03*	-1.51	8.80E-63	161	618
	*DQA1*02:01*	-1.37	8.50E-149	573	1639
	*DQB1*03:01*	-1.16	4.10E-129	759	1773
	*C*08:02*	-0.94	4.00E-29	215	484
	*A*26:01*	-0.63	9.50E-09	137	232
	*C*04:01*	-0.54	9.20E-22	593	882
	*A*32:01*	-0.53	1.30E-11	283	432
	*A*11:01*	-0.50	9.60E-16	472	687
	**genotype**	**ln OR**	**p value**	**N cases**	**N controls**
detrimental	*A*24:02*	0.45	1.10E-19	1284	820
	*B*18:01*	0.73	9.20E-30	827	397
	*A*30:02*	1.08	3.20E-20	305	98
	*DRB1*04:01-DQA1*01:01*	1.25	2.10E-39	522	146
	*DRB1*03:01-DQA1*05:01-DQB1*02:01*	1.34	7.10E-257	3724	1612
	*B*39:06*	1.48	1.10E-31	356	78
	*DRB1*04:05*	1.86	7.90E-28	270	40
	*DRB1*04:01-DQB1*03:02*	2.01	0.00E+00	2849	583
	*DQB1*03:02*	2.04	0.00E+00	3996	1085
	*DRB1*03:01-DQA1*05:01-DQB1*02:01-DQB1*03:02*	2.94	7.20E-264	2177	159

S4 Table. The HLA class I and class II genotypes most closely identified with outcome in the GRID cohort. This table shows the “driver” genotypes which were independently associated

with outcome (see **Fig 3**). The natural log of the odds ratio (ln[OR]), associated p-value for each genotype and number of cases and controls carrying the genotype is shown. ln[OR] and p-values were obtained by multiple logistic regression in the whole cohort with gender as an additional covariate is reported (i.e. the coefficients and p-values are derived in a model containing one genotype at a time).
