# Supplementary material for: Inhibitory KIRs decrease HLA class II-mediated protection in Type 1 Diabetes
Source: PLoS Genet. 2024 Dec 26;20(12):e1011456. doi: 10.1371/journal.pgen.1011456 (PMC11741628; doi:10.1371/journal.pgen.1011456)
Supplement: S5 Table — The UK-GRID cohort without carriers of HLA class I drivers (N = 5,420) was stratified into individuals with high or low iKIR score at different cutoffs (1.5, 1.75 and 2.0). The protective effect of each protective genotype was evaluated independently in each stratum using multiple logistic regression with gender as covariate. HLA class II protection is enhanced in the iKIR low strata for all genotypes but for the very infrequent protective genotype DQA1*02:01-DQB1*03:03. Regression coefficients, permutation p-values and cohort sizes are reported for the different strata. P-value for the whole cohort (unstratified analysis) calculated using the Wald-test; p-values for the stratification analysis are calculated using the permutation test. (PDF) [file pgen.1011456.s022.pdf]

Haplotype	Threshold	Group	lnOR	2.50%	97.50%	P-value	N Genotype +		N Genotype -	
							Cases	Controls	Cases	Controls
DQA1*01:02	Unstratified	Whole cohort	-1.91	-2.07	-1.76	8.14×10^{-133}	252	922	2758	1488
	1.5	iKIR high	-1.75	-1.94	-1.56	5.94×10^{-5}	155	619	1725	1202
		iKIR low	-2.42	-2.69	-2.16		97	303	1033	286
	1.75	iKIR high	-1.63	-1.88	-1.38	7.14×10^{-4}	88	359	999	802
		iKIR low	-2.18	-2.38	-1.98		164	563	1759	686
	2	iKIR high	-1.58	-1.85	-1.33	5.13×10^{-4}	82	318	887	706
		iKIR low	-2.14	-2.33	-1.95		170	604	1871	782
DQA1*01:02-DQB1*05:01	Unstratified	Whole cohort	-2.32	-2.92	-1.80	4.14×10^{-16}	14	109	2996	2301
	1.5	iKIR high	-2.00	-2.70	-1.41	0.12	11	76	1869	1745
		iKIR low	-3.10	-4.53	-2.07		3	33	1127	556
	1.75	iKIR high	-1.80	-2.69	-1.06	0.14	7	44	1080	1117
		iKIR low	-2.71	-3.58	-1.99		7	65	1916	1184
	2	iKIR high	-1.74	-2.64	-1.00	0.13	7	41	962	983
		iKIR low	-2.71	-3.58	-1.99		7	68	2034	1318
DQA1*01:02-DQB1*06:02	Unstratified	Whole cohort	-3.91	-4.33	-3.53	1.76×10^{-83}	26	729	2984	1681
	1.5	iKIR high	-3.45	-3.92	-3.03	3.64×10^{-3}	21	477	1859	1344
		iKIR low	-5.12	-6.16	-4.33		5	252	1125	337
	1.75	iKIR high	-3.12	-3.69	-2.63	1.81×10^{-3}	15	279	1072	882
		iKIR low	-4.58	-5.25	-4.03		11	450	1912	799
	2	iKIR high	-3.09	-3.68	-2.58	4.06×10^{-3}	14	249	955	775
		iKIR low	-4.49	-5.13	-3.96		12	480	2029	906
DQA1*01:03	Unstratified	Whole cohort	-1.75	-2.03	-1.49	2.12×10^{-36}	67	279	2943	2131
	1.5	iKIR high	-1.58	-1.90	-1.28	0.061	52	221	1828	1600
		iKIR low	-2.08	-2.70	-1.53		15	58	1115	531
	1.75	iKIR high	-1.54	-1.93	-1.18	0.26	35	156	1052	1005
		iKIR low	-1.86	-2.27	-1.48		32	123	1891	1126
	2	iKIR high	-1.38	-1.78	-1.01	0.036	35	133	934	891
		iKIR low	-2.00	-2.41	-1.63		32	146	2009	1240
DQA1*02:01	Unstratified	Whole cohort	-1.15	-1.31	-0.99	2.02×10^{-45}	256	548	2754	1862
	1.5	iKIR high	-0.97	-1.15	-0.80	5.14×10^{-3}	235	500	1645	1321
		iKIR low	-1.54	-2.08	-1.03		21	48	1109	541
	1.75	iKIR high	-0.88	-1.10	-0.67	6.15×10^{-3}	149	322	938	839
		iKIR low	-1.32	-1.57	-1.08		107	226	1816	1023
	2	iKIR high	-0.93	-1.16	-0.70	0.044	130	288	839	736

		iKIR low	-1.25	-1.48	-1.03		126	260	1915	1126
DQA1*02:01-DQB1*03:03	Unstratified	Whole cohort	-2.21	-2.87	-1.65	9.05x10^{-13}	12	85	2998	2325
	1.5	iKIR high	-2.12	-2.89	-1.48	0.92	9	70	1871	1751
		iKIR low	-2.26	-3.73	-1.15		3	15	1127	574
	1.75	iKIR high	-2.25	-3.31	-1.43	0.8	5	49	1082	1112
		iKIR low	-2.09	-2.99	-1.34		7	36	1916	1213
	2	iKIR high	-2.11	-3.18	-1.27	0.86	5	42	964	982
		iKIR low	-2.23	-3.12	-1.49		7	43	2034	1343
DQA1*05:05-DQB1*03:01	Unstratified	Whole cohort	-1.74	-1.99	-1.51	6.01x10^{-46}	88	355	2922	2055
	1.5	iKIR high	-1.63	-1.90	-1.37	2.96x10^{-3}	70	300	1810	1521
		iKIR low	-1.85	-2.42	-1.33		18	55	1112	534
	1.75	iKIR high	-1.39	-1.72	-1.08	7.49x10^{-3}	53	198	1034	963
		iKIR low	-2.05	-2.44	-1.69		35	157	1888	1092
	2	iKIR high	-1.40	-1.75	-1.07	1.90x10^{-2}	46	171	923	853
		iKIR low	-1.99	-2.34	-1.65		42	184	1999	1202
DQB1*03:01	Unstratified	Whole cohort	-1.25	-1.39	-1.11	5.59x10^{-69}	361	777	2649	1633
	1.5	iKIR high	-1.05	-1.20	-0.90	5.09x10^{-6}	319	671	1561	1150
		iKIR low	-1.73	-2.12	-1.37		42	106	1088	483
	1.75	iKIR high	-0.85	-1.03	-0.67	<1x10^{-8}	269	505	818	656
		iKIR low	-1.71	-1.96	-1.46		92	272	1831	977
	2	iKIR high	-0.95	-1.15	-0.75	7.09x10^{-4}	213	431	756	593
		iKIR low	-1.44	-1.65	-1.24		148	346	1893	1040
DRB1*07:01-DQA1*02:01-DQB1*05:01	Unstratified	Whole cohort	-1.69	-2.37	-1.10	1.40x10^{-7}	12	52	2998	2358
	1.5	iKIR high	-1.48	-2.16	-0.88	1.14x10^{-2}	12	50	1868	1771
		iKIR low	-14.22	-	45.10		0	2	1130	587
	1.75	iKIR high	-1.13	-1.94	-0.41	6.51x10^{-2}	9	29	1078	1132
		iKIR low	-2.45	-3.89	-1.39		3	23	1920	1226
	2	iKIR high	-1.11	-1.97	-0.35	1.05x10^{-1}	8	25	961	999
		iKIR low	-2.29	-3.51	-1.34		4	27	2037	1359

S5 Table. iKIR score decreases protection associated with protective class II genotypes in T1D. The GRID cohort without carriers of HLA class I drivers (N=5,420) was stratified into individuals with high or low iKIR score at different cutoffs (1.5, 1.75 and 2.0). The

protective effect of each protective genotype was evaluated independently in each stratum using multivariate logistic regression with gender as covariate. HLA class II protection is enhanced in the iKIR low strata for all genotypes but for the very infrequent protective genotype *DQA1*02:01-DQB1*03:03*. Regression coefficients, permutation p-values and cohort sizes are reported for the different strata. P-value for the whole cohort (unstratified analysis) calculated using the Wald-test; p-values for the stratification analysis are calculated using the permutation test.
