# Supplementary material for: Inhibitory KIRs decrease HLA class II-mediated protection in Type 1 Diabetes
Source: PLoS Genet. 2024 Dec 26;20(12):e1011456. doi: 10.1371/journal.pgen.1011456 (PMC11741628; doi:10.1371/journal.pgen.1011456)
Supplement: S6 Table — After removal of class I drivers from the cohort (remaining cohort: N = 5,420), we modelled risk of T1D for each protective genotype and included iKIR score in the model as a continuous variable interacting with the HLA class II genotype (OUTCOME∼HLAclassIIgenotype×iKIR_score). To account for relatedness between individuals, we used a generalized mixed model including a genetic relatedness matrix as a random factor. The coefficient for the interaction term was in the expected direction for all genotypes and significant for 5 frequent genotypes. By comparison with Table 2 (same analysis but without the genetic relatedness matrix) it can be seen that adjusting for cryptic relatedness had little impact (all coefficients and p values very similar, Fig 4). (PDF) [file pgen.1011456.s023.pdf]

Protective genotype	Coefficient of interaction	P-value of interaction	N cases	N controls	N total
*DQA1*01:02-DQB1*06:02*	1	7.03E-06	26	729	755
*DQB1*03:01*	0.42	1.46E-05	361	777	1138
*DQA1*01:02*	0.4	5.09E-05	252	922	1174
*DQA1*05:05-DQB1*03:01*	0.34	3.13E-02	88	355	443
*DQA1*01:02-DQB1*05:01*	0.72	4.94E-02	256	548	804
*DQA1*02:01*	0.21	7.38E-02	14	109	123
*DQA1*01:03*	0.3	8.54E-02	67	279	346
*DRB1*07:01-DQA1*02:01-DQB1*05:01*	0.71	2.03E-01	12	52	64
*DQA1*02:01-DQB1*03:03*	0.16	6.65E-01	12	85	97

S6 Table. iKIR score interaction terms with protective HLA class II drivers adjusted by relatedness in the cohort after exclusion of HLA class I drivers. After removal of class I drivers from the cohort (remaining cohort: N=5,420), we modelled risk of T1D for each protective genotype and included iKIR score in the model as a continuous variable interacting with the HLA class II genotype ($\boldsymbol{OUTCOME} \sim \boldsymbol{HLA\ class\ II\ genotype} \times \boldsymbol{iKIR\_score}$). To account for relatedness between individuals, we used a generalized mixed model including a genetic relatedness matrix as a random factor. The coefficient for the interaction term was in the expected direction for all genotypes and significant for 5 frequent genotypes. By comparison with **Table 2** (same analysis but without the genetic relatedness matrix) it can be seen that adjusting for cryptic relatedness had little impact (all coefficients and p values very similar, **Fig 4**).
