# Supplementary material for: Inhibitory KIRs decrease HLA class II-mediated protection in Type 1 Diabetes
Source: PLoS Genet. 2024 Dec 26;20(12):e1011456. doi: 10.1371/journal.pgen.1011456 (PMC11741628; doi:10.1371/journal.pgen.1011456)
Supplement: S7 Table — The iKIR effect observed in the case-control cohort was validated in an independent family dataset. Trios were stratified into high (>threshold) and low (≤ threshold) iKIR score according to the iKIR score of the child in each trio. For each threshold and each genotype, we calculated the ratio of transmitted to non-transmitted genes in each stratum. The odds of observing an equal or greater difference between log ratios across 4 frequent protective genotypes (DQA1*01:02, DQB1*03:01, DQA1*02:01 and DQA1*01:02-DQB1*06:02) were assessed by permutation test. (PDF) [file pgen.1011456.s024.pdf]

iKIR score threshold	P-value
1	$2.9x10^{-03}$
1.5	$8.8x10^{-03}$
1.75	$1x10^{-05}$
2	$2.1x10^{-04}$
2.5	$2.2x10^{-03}$

S7 Table. Odds of observing an iKIR modification across *DQA1*01:02*, *DQB1*03:01*, *DQA1*02:01* and *DQA1*01:02-DQB1*06:02* in an independent cohort. The iKIR effect observed in the case-control cohort was validated in an independent family dataset. Trios were stratified into high (>threshold) and low (≤ threshold) iKIR score according to the iKIR score of the child in each trio. For each threshold and each genotype, we calculated the ratio of transmitted to non-transmitted genes in each stratum. The odds of observing an equal or greater difference between log ratios across 4 frequent protective genotypes (*DQA1*01:02*, *DQB1*03:01*, *DQA1*02:01* and *DQA1*01:02-DQB1*06:02*) were assessed by permutation test.
