# Supplementary material for: Inhibitory KIRs decrease HLA class II-mediated protection in Type 1 Diabetes
Source: PLoS Genet. 2024 Dec 26;20(12):e1011456. doi: 10.1371/journal.pgen.1011456 (PMC11741628; doi:10.1371/journal.pgen.1011456)
Supplement: S8 Table — The cohort was stratified into individuals with high or low iKIR score using different iKIR score thresholds (1.5, 1.75, 2.0 and 2.5). The protective effect of DRB1*15:01-DQB1*06:02 was evaluated independently in each stratum using multivariate logistic regression with gender included as a covariate. DRB1*15:01-DQB1*06:02 is significantly protective (ln[OR] = –3.9, P = 1.14×10−165) in the cohort (Group = Whole cohort, unstratified analysis). Regression coefficients, p-values and cohort sizes are reported for the different strata. P-value for the unstratified analysis calculated using Wald-test; all other p-values calculated using a permutation test. (PDF) [file pgen.1011456.s025.pdf]

	Group	ln[OR]	2.50%	97.50%	P-value	N haplotype +		N haplotype-	
						Cases	Controls	Cases	Controls
	Whole cohort	-3.87	-4.16	-3.6	2.79E-163	53	1533	6166	4209
Threshold=1.5	iKIR high	-3.56	-3.88	-3.28	2.29E-03	46	1156	4693	3582
	iKIR low	-4.89	-5.75	-4.22		7	377	1473	627
Threshold=1.75	iKIR high	-3.29	-3.68	-2.95	6.17E-04	31	717	2772	2467
	iKIR low	-4.36	-4.82	-3.96		22	816	3394	1742
Threshold=2	iKIR high	-3.33	-3.74	-2.96	5.45E-03	27	625	2401	2071
	iKIR low	-4.24	-4.66	-3.86		26	908	3765	2138
Threshold=2.5	iKIR high	-3.38	-3.82	-3.00	2.40E-02	25	607	2268	1915
	iKIR low	-4.17	-4.57	-3.81		28	926	3898	2294

S8 Table. *DRB1*15:01-DQB1*06:02* protection in T1D is enhanced amongst individuals with a low iKIR score. The cohort was stratified into individuals with high or low iKIR score using different iKIR score thresholds (1.5, 1.75, 2.0 and 2.5). The protective effect of *DRB1*15:01-DQB1*06:02* was evaluated independently in each stratum using multivariate logistic regression with gender included as a covariate. *DRB1*15:01-DQB1*06:02* is significantly protective (ln[OR]= -3.9, $P=1.14x10^{-165}$) in the cohort (Group=Whole cohort, unstratified analysis). Regression coefficients, p-values and cohort sizes are reported for the different strata. P-value for the unstratified analysis calculated using Wald-test; all other p-values calculated using a permutation test.
