# Supplementary material for: Inhibitory KIRs decrease HLA class II-mediated protection in Type 1 Diabetes
Source: PLoS Genet. 2024 Dec 26;20(12):e1011456. doi: 10.1371/journal.pgen.1011456 (PMC11741628; doi:10.1371/journal.pgen.1011456)
Supplement: S9 Table — Individuals were stratified into High, Intermediate and Low iKIR score categories using 6 different definitions of high, intermediate, and low (i.e. 6 different strata choices). For all strata choices we observe the same picture, that DRB1*15:01-DQB1*06:02 protection increases as iKIR score decreases as we observed for DQA1*01:02-DQB1*06:02 (see S3 Fig). (PDF) [file pgen.1011456.s026.pdf]

Strata choice	Group		ln[OR]	2.50%	97.50%	N genotype+		N genotype-	
						Cases	Controls	Cases	Controls
1	High	(2.75,4]	-2.32	-2.95	-1.78	13	125	514	481
	Int	(1.75,2.75]	-3.62	-4.13	-3.18	18	592	2258	1986
	Low	[0,1.75]	-4.28	-4.74	-3.88	22	816	3394	1742
2	High	(3,4]	-2.34	-3.00	-1.77	12	119	470	447
	Int	(1.75,3]	-3.58	-4.08	-3.15	19	598	2302	2020
	Low	[0,1.75]	-4.28	-4.74	-3.88	22	816	3394	1742
3	High	(2.75,4]	-2.32	-2.95	-1.78	13	125	514	481
	Int	(2,2.75]	-3.75	-4.33	-3.25	14	500	1887	1590
	Low	[0,2]	-4.12	-4.54	-3.75	26	908	3765	2138
4	High	(3,4]	-2.34	-3.00	-1.77	12	119	470	447
	Int	(2,3]	-3.70	-4.26	-3.21	15	506	1931	1624
	Low	[0,2]	-4.12	-4.54	-3.75	26	908	3765	2138
5	High	(2.75,4]	-2.32	-2.95	-1.78	13	125	514	481
	Int	(2.5,2.75]	-3.90	-4.53	-3.37	12	482	1754	1434
	Low	[0,2.5]	-4.03	-4.43	-3.67	28	926	3898	2294
6	High	(3,4]	-2.34	-3.00	-1.77	12	119	470	447
	Int	(2.5,3]	-3.83	-4.44	-3.32	13	488	1798	1468
	Low	[0,2.5]	-4.03	-4.43	-3.67	28	926	3898	2294

S9 Table. iKIR score impacts *DRB1*15:01-DQB1*06:02* protection in a dose-dependent manner. Individuals were stratified into High, Intermediate and Low iKIR score categories using 6 different definitions of high, intermediate, and low (i.e. 6 different strata choices). For all strata choices we observe the same picture, that *DRB1*15:01-DQB1*06:02* protection increases as iKIR score decreases as we observed for *DQA1*01:02-DQB1*06:02* (see **S3 Fig**).
