# Supplementary material for: Inhibitory KIRs decrease HLA class II-mediated protection in Type 1 Diabetes
Source: PLoS Genet. 2024 Dec 26;20(12):e1011456. doi: 10.1371/journal.pgen.1011456 (PMC11741628; doi:10.1371/journal.pgen.1011456)
Supplement: S10 Table — DR3 and DR4 haplotypes were included as covariates and standardised iKIR score was included as an interaction term for comparison. We denote the strata DR3 or DR4 for ease of reference, but we are considering genes both in cis or in trans and only considering DRB1 and DQB1 genes. (PDF) [file pgen.1011456.s027.pdf]

Covariates	Coefficient of interaction	P-value of interaction
GENDER + *DR3*	+0.67	$1.9x10^{-6}$
GENDER + *DR4*	+0.70	$1x10^{-6}$
GENDER + *DR3* + *DR4*	+0.67	$3.4x10^{-6}$

S10 Table. iKIR score effect on *DR*15:01-DQ*06:02* is independent of *DR3* and *DR4* haplotypes. *DR3* and *DR4* haplotypes were included as covariates and standardised iKIR score was included as an interaction term for comparison. We denote the strata *DR3* or *DR4* for ease of reference, but we are considering genes both in cis or in trans and only considering *DRB1* and *DQB1* genes.
