# Supplementary material for: Inhibitory KIRs decrease HLA class II-mediated protection in Type 1 Diabetes
Source: PLoS Genet. 2024 Dec 26;20(12):e1011456. doi: 10.1371/journal.pgen.1011456 (PMC11741628; doi:10.1371/journal.pgen.1011456)
Supplement: S11 Table — Individuals carrying a given HLA class I allele (Allele) are removed from the UK-GRID cohort and then the subcohort is modeled with iKIR as an interaction term with DRB1*15:01-DQB1*06:02. Coefficients (ln[OR]) and p-values for the interaction term are reported. (PDF) [file pgen.1011456.s028.pdf]

Allele	ln[OR]	P-value		Allele	ln[OR]	P-value		Allele	ln[OR]	P-value		Allele	ln[OR]	P-value
A0101	0.54	2.80E-03		*B0801*	0.66	2.16E-04		*B5501*	0.74	4.11E-06		*C0304*	0.77	1.42E-05
A0201	0.77	2.31E-03		*B1501*	0.72	3.10E-05		*B4901*	0.79	9.39E-07		*C0303*	0.79	2.45E-06
A2402	0.86	3.21E-06		*B4402*	0.81	2.64E-06		*B4002*	0.76	1.62E-06		*C0501*	0.88	3.79E-07
A3201	0.79	9.36E-07		*B3501*	0.73	5.55E-06		*B4101*	0.75	2.08E-06		*C0701*	0.70	1.48E-04
A1101	0.76	3.62E-06		*B1401*	0.79	1.12E-06		*B1503*	0.75	2.35E-06		*C0401*	0.74	3.46E-06
A2301	0.75	2.16E-06		*B4403*	0.74	4.64E-06		*B2702*	0.75	2.29E-06		*C0802*	0.84	3.86E-07
A0301	0.92	6.42E-05		*B0702*	1.00	6.02E-04		*B4501*	0.75	2.22E-06		*C0602*	0.61	3.03E-04
A2902	0.73	6.21E-06		*B3901*	0.74	2.93E-06		*B4102*	0.75	2.56E-06		*C1203*	0.71	1.52E-05
A2501	0.72	1.33E-05		*B1801*	0.76	6.65E-06		*B4405*	0.75	2.09E-06		*C0202*	0.74	5.35E-06
A0205	0.77	1.94E-06		*B3801*	0.75	2.44E-06		*B1510*	0.75	2.29E-06		*C0702*	1.17	5.29E-04
A3002	0.76	1.73E-06		*B4001*	0.84	9.62E-07		*B5701*	0.67	2.91E-05		*C0302*	0.75	2.29E-06
A0206	0.75	2.23E-06		*B3906*	0.78	4.48E-06		*B5301*	0.75	2.33E-06		*C0102*	0.72	6.13E-06
A2601	0.74	2.73E-06		*B1302*	0.77	2.16E-06		*B5108*	0.75	2.31E-06		*C1601*	0.72	6.84E-06
A3101	0.77	4.44E-06		*B2705*	0.71	1.15E-05		*B1516*	0.75	2.29E-06		*C0704*	0.75	2.40E-06
A3001	0.74	3.43E-06		*B1402*	0.80	8.50E-07		*B1508*	0.75	2.30E-06		*C1502*	0.71	1.26E-05
A6801	0.74	3.01E-06		*B5201*	0.72	7.70E-06		*B4006*	0.75	2.26E-06		*C1202*	0.72	7.72E-06
A0202	0.75	2.25E-06		*B5101*	0.72	8.95E-06		*B4801*	0.75	2.29E-06		*C1402*	0.74	2.71E-06
A3301	0.75	2.20E-06		*B1517*	0.74	3.66E-06		*B5601*	0.75	2.25E-06		*C1505*	0.75	2.13E-06
A3004	0.75	2.33E-06		*B4701*	0.77	1.47E-06		*B3924*	0.75	2.24E-06		*C0210*	0.75	2.29E-06
A2901	0.75	2.38E-06		*B5001*	0.76	1.67E-06		*B5801*	0.75	2.20E-06		*C1701*	0.75	2.40E-06
A6601	0.75	2.25E-06		*B3701*	0.66	4.66E-05		*B7301*	0.75	2.26E-06		*C1602*	0.75	2.06E-06
A3303	0.75	2.41E-06		*B3503*	0.75	2.24E-06						*C1604*	0.75	2.24E-06
A3402	0.75	2.34E-06		*B1518*	0.75	2.57E-06						*C1403*	0.75	2.29E-06
A6802	0.75	2.13E-06		*B0705*	0.75	2.17E-06						*C0310*	0.75	2.32E-06
A6901	0.75	2.30E-06		*B3502*	0.75	2.04E-06						*C0803*	0.75	2.29E-06
A7403	0.75	2.29E-06		*B3508*	0.76	1.92E-06								

S11 Table. iKIR interaction remains significant in all HLA class I allele negative subcohorts. Individuals carrying a given HLA class I allele (Allele) are removed from the GRID cohort and then the subcohort is modeled with iKIR as an interaction term with *DRB1*15:01-DQB1*06:02*. Coefficients (ln[OR]) and p-values for the interaction term are reported.
