# Supplementary material for: Inhibitory KIRs decrease HLA class II-mediated protection in Type 1 Diabetes
Source: PLoS Genet. 2024 Dec 26;20(12):e1011456. doi: 10.1371/journal.pgen.1011456 (PMC11741628; doi:10.1371/journal.pgen.1011456)
Supplement: S12 Table — The UK-GRID cohort was stratified into individuals with high or low iKIR score using different cutoffs (1.5, 1.75, 2.0 and 2.5). The protective effect of DQ6 was evaluated independently in each stratum using multivariate logistic regression with gender, Bw4, C1 and C2 ligands included in the model as covariates. Overall conclusions were remarkably similar to our previous analysis (not including the ligands as covariates). Regression coefficients, permutation p-values and cohort sizes are reported for the different strata. P-value for the whole cohort (unstratified analysis) calculated using the Wald-test; p-values for the stratification analysis are calculated using the permutation test. (PDF) [file pgen.1011456.s029.pdf]

	Group	ln[OR]	2.50%	97.50%	P-value	N haplotype +		N haplotype-	
						Cases	Controls	Cases	Controls
	Whole cohort	-3.86	-4.15	-3.59	$1.3x10^{-165}$	54	1545	6165	4197
Threshold=1.5	iKIR high	-3.55	-3.86	-3.27	$2.0x10^{-3}$	47	1165	4692	3573
	iKIR low	-4.27	-4.80	-3.82		7	380	1473	624
Threshold=1.75	iKIR high	-3.27	-3.65	-2.93	$2.8x10^{-4}$	32	721	2771	2463
	iKIR low	-4.38	-4.84	-3.98		22	824	3394	1734
Threshold=2	iKIR high	-3.30	-3.70	-2.93	$2.6x10^{-3}$	28	629	2400	2067
	iKIR low	-4.25	-4.67	-3.88		26	916	3765	2130
Threshold=2.5	iKIR high	-3.35	-3.78	-2.98	$9.2x10^{-3}$	26	611	2267	1911
	iKIR low	-4.18	-4.59	-3.82		28	934	3898	2286

S12 Table. iKIR score negatively impacts protection associated with *DQ6* in T1D even when iKIR ligands are included as covariates. The GRID cohort was stratified into individuals with high or low iKIR score using different cutoffs (1.5, 1.75, 2.0 and 2.5). The protective effect of *DQ6* was evaluated independently in each stratum using multivariate logistic regression with gender, Bw4, C1 and C2 ligands included in the model as covariates. Overall conclusions were remarkably similar to our previous analysis (not including the ligands as covariates). Regression coefficients, permutation p-values and cohort sizes are reported for the different strata. P-value for the whole cohort (unstratified analysis) calculated using the Wald-test; p-values for the stratification analysis are calculated using the permutation test.
