# Supplementary material for: Inhibitory KIRs decrease HLA class II-mediated protection in Type 1 Diabetes
Source: PLoS Genet. 2024 Dec 26;20(12):e1011456. doi: 10.1371/journal.pgen.1011456 (PMC11741628; doi:10.1371/journal.pgen.1011456)
Supplement: S13 Table — Individuals carrying a given HLA class I allele (Allele) are removed from the UK-GRID cohort and then the risk of T1D is modeled with iKIR as an interaction term with DQ6 in the allele-negative subcohort. Coefficients (ln[OR]) and p-values for the interaction term are reported. (PDF) [file pgen.1011456.s030.pdf]

Allele	ln[OR]	P-value	Allele	ln[OR]	P-value	Allele	ln[OR]	P-value	Allele	ln[OR]	P-value
A0101	0.54	2.86E-03	*B0801*	0.66	2.24E-04	*B5501*	0.77	1.19E-06	*C0304*	0.81	4.24E-06
A0201	0.86	5.78E-04	*B1501*	0.75	9.79E-06	*B4901*	0.82	2.60E-07	*C0303*	0.83	6.72E-07
A2402	0.91	6.95E-07	*B4402*	0.85	6.18E-07	*B4002*	0.80	4.57E-07	*C0501*	0.92	7.82E-08
A3201	0.83	2.54E-07	*B3501*	0.76	1.61E-06	*B4101*	0.79	5.92E-07	*C0701*	0.70	1.53E-04
A1101	0.79	1.09E-06	*B1401*	0.82	3.09E-07	*B1503*	0.78	6.75E-07	*C0401*	0.78	9.90E-07
A2301	0.78	6.19E-07	*B4403*	0.77	1.39E-06	*B2702*	0.79	6.55E-07	*C0802*	0.88	1.06E-07
A0301	0.98	1.81E-05	*B0702*	1.09	1.47E-04	*B4501*	0.78	6.35E-07	*C0602*	0.66	8.85E-05
A2902	0.76	1.86E-06	*B3901*	0.78	8.48E-07	*B4102*	0.78	7.39E-07	*C1203*	0.75	4.46E-06
A2501	0.76	3.87E-06	*B1801*	0.80	1.89E-06	*B4405*	0.79	5.97E-07	*C0202*	0.78	1.48E-06
A0205	0.81	5.45E-07	*B3801*	0.78	7.05E-07	*B1510*	0.79	6.56E-07	*C0702*	1.28	1.16E-04
A3002	0.79	4.89E-07	*B4001*	0.87	2.57E-07	*B5701*	0.71	8.42E-06	*C0302*	0.79	6.55E-07
A0206	0.79	6.37E-07	*B3906*	0.82	1.18E-06	*B5301*	0.78	6.68E-07	*C0102*	0.76	1.83E-06
A2601	0.78	7.90E-07	*B1302*	0.81	5.98E-07	*B5108*	0.78	6.63E-07	*C1601*	0.76	2.07E-06
A3101	0.77	4.09E-06	*B2705*	0.75	3.33E-06	*B1516*	0.78	6.57E-07	*C0704*	0.78	7.03E-07
A3001	0.78	9.89E-07	*B1402*	0.83	2.43E-07	*B1508*	0.79	6.59E-07	*C1502*	0.71	1.17E-05
A6801	0.78	8.75E-07	*B5201*	0.75	2.28E-06	*B4006*	0.79	6.45E-07	*C1202*	0.75	2.28E-06
A0202	0.79	6.43E-07	*B5101*	0.72	8.36E-06	*B4801*	0.79	6.55E-07	*C1402*	0.78	7.86E-07
A3301	0.79	6.27E-07	*B1517*	0.78	1.05E-06	*B5601*	0.79	6.43E-07	*C1505*	0.79	6.08E-07
A3004	0.78	6.68E-07	*B4701*	0.80	4.11E-07	*B3924*	0.79	6.41E-07	*C0210*	0.79	6.56E-07
A2901	0.78	6.83E-07	*B5001*	0.79	4.71E-07	*B5801*	0.79	6.30E-07	*C1701*	0.78	6.90E-07
A6601	0.79	6.45E-07	*B3701*	0.70	1.43E-05	*B7301*	0.79	6.46E-07	*C1602*	0.79	5.86E-07
A3303	0.78	6.90E-07	*B3503*	0.78	6.42E-07				*C1604*	0.79	6.42E-07
A3402	0.78	6.69E-07	*B1518*	0.78	7.50E-07				*C1403*	0.79	6.56E-07
A6802	0.79	6.06E-07	*B0705*	0.79	6.20E-07				*C0310*	0.78	6.66E-07
A6901	0.79	6.59E-07	*B3502*	0.79	5.81E-07				*C0803*	0.79	6.55E-07
A7403	0.79	6.55E-07	*B3508*	0.80	5.36E-07						

S13 Table. iKIR interaction remains significant in all HLA class I allele negative subcohorts. Individuals carrying a given HLA class I allele (Allele) are removed from the GRID cohort and then the risk of T1D is modeled with iKIR as an interaction term with *DQ6* in the allele-negative subcohort. Coefficients (ln[OR]) and p-values for the interaction term are reported.
