# Supplementary material for: Inhibitory KIRs decrease HLA class II-mediated protection in Type 1 Diabetes
Source: PLoS Genet. 2024 Dec 26;20(12):e1011456. doi: 10.1371/journal.pgen.1011456 (PMC11741628; doi:10.1371/journal.pgen.1011456)
Supplement: S14 Table — DR3 (defined here to be DRB1*03:01-DQB1*02:01 in cis or in trans) and DR4 (defined to be DRB1*04:01/02/04/05-DQB1*03:02 in cis or in trans) detrimental genotypes were included as covariates and standardised iKIR score was included as an interaction term for comparison. (PDF) [file pgen.1011456.s031.pdf]

Covariates	Coefficient of interaction	P-value of interaction
GENDER + *DR3*	+0.69	$5.3x10^{-7}$
GENDER + *DR4*	+0.73	$3.4x10^{-7}$
GENDER + *DR3* + *DR4*	+0.69	$1.3X10^{-6}$

S14 Table. iKIR score effect on *DQ6* is independent of *DR3* and *DR4* detrimental genotypes.

DR3 (defined here to be *DRB1*03:01-DQB1*02:01 in cis* or *in trans*) and *DR4* (defined to be *DRB1*04:01/02/04/05-DQB1*03:02 in cis* or *in trans*) detrimental genotypes were included as covariates and standardised iKIR score was included as an interaction term for comparison.
