# Supplementary material for: Inhibitory KIRs decrease HLA class II-mediated protection in Type 1 Diabetes
Source: PLoS Genet. 2024 Dec 26;20(12):e1011456. doi: 10.1371/journal.pgen.1011456 (PMC11741628; doi:10.1371/journal.pgen.1011456)
Supplement: S15 Table — (PDF) [file pgen.1011456.s032.pdf]

Equation	Parameter	Model	Definition	Units	Value	Reference
β-cell (B)	δ_β	1&2	β cell killing per effector T cell	$cell^{-1}day^{-1}$	$1x10^{-5}$ – $9x10^{-5}$	Estimated
Islet antigen (A)	α_A	1&2	Rate of islet antigen release (or beta cell damage) per Tconv	$cell^{-1}day^{-1}$	$1x10^{-5}$	[1]
	δ_A	1&2	Clearance rate of the antigen	day^{-1}	1	[1]
T_{regs} (R)	α_R	1&2	Proliferation rate of Tregs upon antigen encounter	day^{-1}/ $cell^{-1}day^{-1}$	1 – 10	[2]
	δ_T	1&2	Death rate of Tregs and Tconvs	day^{-1}	0.1 – 0.2	[3]
	K_R	2	Density control on Treg proliferation rate	cells	γ_R – 10	[2]
T_{convs} (C)	α_C	1&2	Proliferation rate of Tconvs dependent of antigen activation of Tnaive and memory cells	day^{-1}	1 – 100	[2]
	δ_i	1	T conv suppression by Tregs	$cell^{-1}day^{-1}$	1	Estimated
	k	2	Threshold of Tconv inhibition by Treg	cells	1	Estimated
	K_C	2	Density control on Tconv proliferation rate	cells	γ_C – 100	[2]

S15 Table. Parameters used in the mathematical model of Beta cell destruction.

References

1. Mahaffy JM, Edelstein-Keshet L. Modeling Cyclic Waves of Circulating T Cells in Autoimmune Diabetes. SIAM Journal on Applied Mathematics. 2007;67(4):915-37. doi: 10.1137/060661144.
2. Babon JA, DeNicola ME, Blodgett DM, Crevecoeur I, Buttrick TS, Maehr R, et al. Analysis of self-antigen specificity of islet-infiltrating T cells from human donors with type 1 diabetes. Nat Med. 2016;22(12):1482-7. doi: 10.1038/nm.4203.
3. Murphy KM, Weaver C, Berg L, Barton G. Janeway's Immunobiology: W.W. Norton; 2022. doi.
