# Supplementary material for: Inhibitory KIRs decrease HLA class II-mediated protection in Type 1 Diabetes
Source: PLoS Genet. 2024 Dec 26;20(12):e1011456. doi: 10.1371/journal.pgen.1011456 (PMC11741628; doi:10.1371/journal.pgen.1011456)
Supplement: S16 Table — (PDF) [file pgen.1011456.s033.pdf]

Population	Antibody	Supplier	Product code
Dump	CD14 V500	BD Horizon	561391
	CD19 V500	BD Horizon	561121
	Live/dead fixable Aqua	Thermo Fisher	L34966
KIR2DL1/S5	KIR2DL1/S5 FITC	R&D Systems	FAB1844F
KIR3DL1	KIR3DL1 PE	Biolegend	312708
CD45RA	CD45RA ECD	Beckman Coulter	B49193
CD4	CD4 PECy5.5	Thermo Fisher	MHCD0418
CD56	CD56 PECy7	Biolegend	318318
KIR2DL2/L3	KIR2DL2/DL3 APC	Biolegend	312612
CD3	CD3 APC Fire750	Biolegend	344840
CD28	CD28 BV421	BD Horizon	562613
CD8	CD8a BV711	Biolegend	301044
CD16	CD16 BV785	Biolegend	302046

S16 Table. Flow cytometry panel.
